# Biological activities of meroterpenoids isolated from different sources

**DOI:** 10.3389/fphar.2022.830103

**Published:** 2022-09-19

**Authors:** Neeraj Kumar Fuloria, Radhika K. Raheja, Kaushal H. Shah, Manisha J. Oza, Yogesh A. Kulkarni, Vetriselvan Subramaniyan, Mahendran Sekar, Shivkanya Fuloria

**Affiliations:** ^1^ Faculty of Pharmacy, AIMST University, Bedong, Malaysia; ^2^ SVKM’s Dr. Bhanuben Nanavati College of Pharmacy, Mumbai, India; ^3^ Shobhaben Pratapbhai Patel School of Pharmacy & Technology Management, SVKM’s NMIMS, Mumbai, India; ^4^ Faculty of Medicine, Bioscience and Nursing, MAHSA University, Selangor, Malaysia; ^5^ Department of Pharmaceutical Chemistry, Faculty of Pharmacy and Health Sciences, Royal College of Medicine Perak, Universiti Kuala Lumpur, Ipoh, Malaysia

**Keywords:** cytotoxicity, anti-inflammatory, anti-proliferative, anti-microbial, anti-fungal, anti-viral, anti-oxidant, meroterpenoids

## Abstract

Meroterpenoids are natural products synthesized by unicellular organisms such as bacteria and multicellular organisms such as fungi, plants, and animals, including those of marine origin. Structurally, these compounds exhibit a wide diversity depending upon the origin and the biosynthetic pathway they emerge from. This diversity in structural features imparts a wide spectrum of biological activity to meroterpenoids. Based on the biosynthetic pathway of origin, these compounds are either polyketide-terpenoids or non-polyketide terpenoids. The recent surge of interest in meroterpenoids has led to a systematic screening of these compounds for many biological actions. Different meroterpenoids have been recorded for a broad range of operations, such as anti-cholinesterase, COX-2 inhibitory, anti-leishmanial, anti-diabetic, anti-oxidative, anti-inflammatory, anti-neoplastic, anti-bacterial, antimalarial, anti-viral, anti-obesity, and insecticidal activity. Meroterpenoids also possess inhibitory activity against the expression of nitric oxide, TNF- α, and other inflammatory mediators. These compounds also show renal protective, cardioprotective, and neuroprotective activities. The present review includes literature from 1999 to date and discusses 590 biologically active meroterpenoids, of which 231 are from fungal sources, 212 are from various species of plants, and 147 are from marine sources such as algae and sponges.

## Introduction

The name “meroterpenoid” was conceived by Cornforth for a group of secondary metabolites, which are partially derived from the terpenoid biosynthetic pathway (Matsuda and Abe, 2016). Meroterpenoids have wide structural diversity consisting of a prenyl unit connected to a phenolic derivative from basic compounds to the more complex meroterpenoids consisting of functionalized carbon chains (Geris and Simpson, 2009a). The diversity is observed not only in the non-terpenoid component of the structure but also in the chain length of the terpenoid and the mode in which the terpenoid portion of the molecule undergoes cyclization. These compounds are derived from various natural sources, such as animals, fungi, marine organisms, and plants (Matsuda and Abe, 2016). However, fungi and aquatic organisms are the richest sources of meroterpenoids (El-Demerdash et al., 2020a). Higher plants from genera such as *Psidium*, *Eucalyptus*, *Arnebia*, and *Eugenia* show the presence of biologically active meroterpenoids.

The classification of meroterpenoids was based on the biosynthetic pathway of origin of these compounds: the initial classification focused on the chemical composition of the polyketide-terpenoid and non-polyketide-terpenoid components (Geris and Simpson, 2009b). Some researchers relied on the same terpene component, whereas a few others realized that the immense diversity and complexity of the structures of the non-terpenoid component should help define the meroterpenoids chemically. Broadly, the meroterpenoids of fungal origin fall under three major categories: those possessing triketide-terpenoid scaffold, those with tetraketide-terpenoid scaffold, and those containing indole-3-glycerolphosphate moiety. This rigid classification fits in a wide variety of aromatic and non-aromatic polar molecules, possessing groups such as the carboxylic acid, hydroxy group, and lactone/ester moieties in the non-terpenoid component. Subtle changes in the stereochemistry of the attached substituents bring these groups in close spatial vicinity, which aids the formation of unique groups such as epoxide, imparting such isomers’ modified biological potency. Non-polyketide terpenoids are derived from the shikimic acid pathway and include quinine derivatives, dehydroquinic acid, protocatechuic acid derivatives, or subunits attached to terpenoid moiety with one C-C bond. On the contrary, polyketides are a large family of natural compounds synthesized by fungi, plants, or bacteria by condensing carboxylic acid compounds. The polyketide moiety is predominant in meroterpenoids derived from fungi (Birch, 1967). Meroterpenoids with the 5/6/6/6 or the 6/6/6/6 tetracyclic rings seemed to be formed through the mevalonate pathway. Jiang et al. reported a comprehensive analysis of the chemical scaffolds seen in meroterpenoids and a distribution of the meroterpenoids discovered in the last decade within these classes (Jiang et al., 2021). Similarly, the focus on the chemical diversity of meroterpenoids from fungi of marine origin by El-Demerdash et al. proves useful in comprehending the structural features of the meroterpenoids (El-Demerdash et al., 2020a).

Meroterpenoid compounds have been studied in the recent decade for a wide spectrum of biological activity. These compounds possess many activities such as anti-cholinesterase, alpha-glucosidase, COX-2 inhibitory, anti-bacterial, anti-viral, anti-leishmanial, anti-obesity, anti-diabetic, anti-oxidative, anti-neoplastic, insecticidal, and cardioprotective. This diverse but promising spectrum of biological activities has also surged a simultaneous interest in the study of total synthesis of meroterpenoids; to name a few, berkeleyone A, from a fungal origin, merochlorins A and B, from marine origin, lingzhiol, from various species of mushrooms, and tomentosenol A and (±)-guajadial B from a plant origin have been explored for total synthesis (Liu et al.; Gao et al., 2012; Teufel et al., 2014; Gautam, 2016; Yu et al., 2016; Elkin et al., 2017). Semisynthetic analogs from isocupressic acid (strongylophorines), (+)-bicyclogermacrene ((+)-ledene, (+)-viridiflorol, (-)-patrol, (+)-spathulenol, and psiguadials A, C, and D) and many others have also been structurally explored (Tran and Cramer, 2014; Yu et al., 2016). Even several workers have scrutinized the structure-activity relationships of meroterpenoids to improve the observed biological activity. Limited review articles are published on meroterpenoids. The first review of meroterpenoid obtained from fungi was published by Shiomi et al. (1999). Later. Geris and Simpson (2009a) published one more review of meroterpenoids obtained from fungi, and the review was mainly focused on the phytochemistry aspects of meroterpenoids. Then, Matsuda and Abe (2016) published a review of the biosynthesis of meroterpenoids from fungi. Recently, two reviews have been published on the chemistry and biology of meroterpenoids derived only from fungi (El-Demerdash et al., 2020b; Jiang et al., 2021). However, a comprehensive review of meroterpenoids derived from different sources such as plants, fungi, and marine sources is unavailable. Thus, the present review mainly focuses on meroterpenoids from these sources with respect to chemistry, biological activity, and the synthesis approach of biologically active meroterpenoids.

## Methods

The data have been collected from various sources such as PubMed, ScienceDirect, Scopus, ProQuest, EBSCO, and google scholar. Research and review articles from the year 1999 onward were thoroughly reviewed. Meroterpenoids, fungi, algae, and plants in combination with meroterpenoids have been used as keywords to collect the data.

### Strategies for total or partial synthesis of meroterpenoids

The natural biosynthesis of meroterpenoids involves the pathways of terpenoids and polyketide synthesis, which makes the overall process intriguing. Considering the complex stereochemistry existing within the meroterpenoids makes synthesizing pure enantiomers synthetically a challenging and humongous task. Several researchers have reported the total synthesis of meroterpenoids or precursor molecules leading to the synthesis of meroterpenoids. Strongylophorines; gujadial; psidial A; (+) yahazunol; guadials B and C; guapsidial A and psiguajadial D; drimane meroterpenoids; naphthoquinone-based meroterpenoids; ganocins B and C; (+) ledene; (+)-viridiflorol; (-)-palustrol; (+)-spathulenol; psiguadials A, C, and D; (±) berkeleyone A; and biscognienyne B have been attempted (Laube et al., 2002; Lawrence et al., 2010; Tran and Cramer, 2014; Liu Y. et al., 2016; Yu et al., 2016; Elkin et al., 2017; Miles et al., 2017; Dethe et al., 2018; Wang et al., 2020). Petrovčič et al. have critically reviewed the synthesis protocols adopted by various studies that have attempted the total synthesis of meroterpenoids since 2015. Cycloadditions, Suzuki reaction, Diels Alder reaction using dienophiles such as caryophyllene and α-humulene, and groups leading to innovative polyene cyclization termination have been thoroughly exploited for the total synthetic procedures. Similarly, chemoenzymatic methods have been exploited for oxidation reactions in several methods (Petrovčič et al., 2021).

## Biological activities of meroterpenoids

### Cytotoxic activity of meroterpenoids

#### Cytotoxicity studies of meroterpenoids isolated from the fungus

Meroterpenoids of different types isolated from various fungal species such as *Phoma* sp., *Pseudocosmospora* sp., *Ascochyta viciae* Lib., *Neosetophoma*, *Ganoderma cochlear* (Blume & T. Nees) Bres., *Stachybotrys chartarum* (Ehrenb.), *Antrodia cinnamomea* (Chang & Chou), *Streptomyces* sp., *Neosartorya spinosa* (Raper & Fennell) Kozak., *Emericella nidulans*, *Gliomastix* sp., *Xylaria humosa*, *Penicillium* sp., *Eurotium chevalieri*, *Guignardia mangiferae* A.J. Roy, *Peyronellaea coffeae-arabicae* FT238, *Aspergillus terreus* Thom, *Aspergillus insuetus* (Bainier) Thom & Church, *Stachybotrys bisbyi* G.L. Barron, and *Pestalotiopsis fici* have been reported for their moderate-to-potent cytotoxic effect in various cancer cell lines.

**FIGURE 1:**
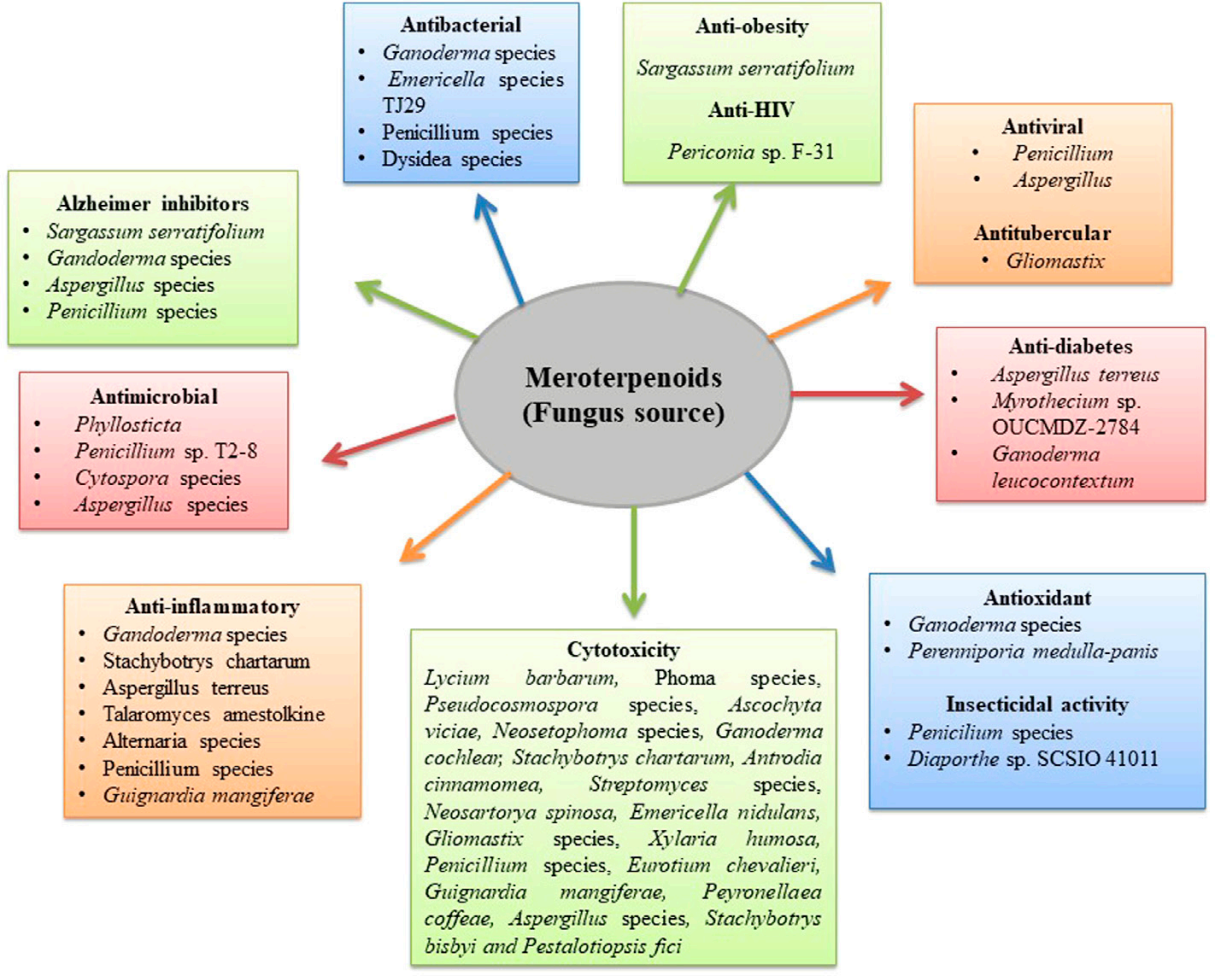
Biological activity of fungus meroterpenoids.

Nakamura et al. reported the cytotoxic effect of two isolated meroterpenoids, namely, rel-(6′S, 10′R)-decarboxy-Δ^9^-tetrahydrocannabinolic acid B and rel-(6′S, 10′R)-Δ^9^-tetrahydrocannabinolic acid B, against promyelocytic leukemia (HL60) with IC_50_ of 1.6 and 24.1 μM, respectively (Nakamura et al., 2019). Qin et al. isolated dimeric meroterpenoid compounds from *Ganoderma cochlear* (Blume & T. Nees) Bres. fruiting bodies, namely, (+) and (-)-gancochlearols A and B, and cochlearoids N–P. The study demonstrated that (+) and (-)-gancochlearols A and B were cytotoxic against erythroleukemic and hepatocarcinoma cells and also inhibited COX-2 expression (Qin et al., 2018b). Cochlearoids N and P showed a potent cytotoxic effect against erythroleukemia-type cells (Qin F.-Y. et al., 2019). Two more meroterpenoids, gancochlearol D and ganomycin F, have been reported for their cytotoxic effect against lung cancer cells of various types, with ganomycin F being more potent than gancochlearol D (Cheng et al., 2018). Spirocochlealactones A–C also have a potential cytotoxic effect against A549, Huh-7, and K562 cancer cell lines (Qin F.-Y. et al., 2018). Zhang et al. isolated two tropolonic meroterpenoids, phomanolides D and F, which exhibited a cytotoxic effect against glioma, breast cancer, and cervical cancer cells (Zhang et al., 2019c). Ascochlorin isolated from *Ascochyta viciae* also showed a potent cytotoxic effect on breast cancer cells (Quan et al., 2019). Eupenifeldin and dehydroxyeupenifeldin isolated from *Neosetophoma* reported a cytotoxic effect against a board cancer cell lines (i.e., ovarian, breast, lung cancer, and mesothelioma cells) (El-Elimat et al., 2019). Jagels et al. isolated moderately cytotoxic meroterpenoids, stachybotrychromenes A and B, from *Stachybotrys chartarum* (Ehrenb.) (Jagels et al., 2018). Antroquinonol A biosynthesized by the fungus *Antrodia cinnamomea* (Chang & Chou) has been reported as a potent tumor growth inhibitor against lung and prostate cancer with GI_50_ values of 13.5 ± 0.2 and 5.7 ± 0.2 μM. Furthermore, antroquinonol V reported growth inhibitory activity with GI_50_ values of 8.2 ± 0.8 μM against lung cells (Chen M. C. et al., 2017). Quinadoline A, 1-hydroxychevalone C, 1,11-dihydroxychevalone C, and 1-acetoxychevalone C, isolated from the fungus *Neosartorya spinosa* (Raper & Fennell) Kozak., displayed cytotoxicity against lung and breast cancer cells (Rajachan et al., 2016). Emeriphenolicins E, which is an isoindolone containing meroterpenoid isolated from *Emericella nidulans*, has been reported with a potent cytotoxic effect in hepatic cancer cells (Zhou et al., 2016). Purpurogemutantin, macrophorin A, 4′-oxomacrophorin, 2,3-hydrodeacetoxyyanuthone A, 22-deacetylyanuthone A, and anicequol isolated from fungus *Gliomastix* sp. exhibited potent-to-moderate cytotoxic effect in various cell lines (He W. J. et al., 2017). Arisugacin B and arisugacin F isolated from the fungus *Penicillium* sp. exhibited weak cytotoxicity with IC_50_ values in the range of 24–60 µM against cervical cancer and leukemia cells (Sun et al., 2014). Sodngama et al. isolated chevalones B and C and reported their cytotoxicity activity against the human lung cancer cell line, NCI-H187, with IC_50_ values of 21.4 and 17.7 μg/ml (Sodngam et al., 2014). An unprecedented terpenoid-polyketide meroterpenoid (isopenicin A) isolated from the culture of *Penicillium* sp. sh18 exhibited stronger growth inhibitory effects on colon cancer cells. Isopenicin A selectively suppresses the Wnt signaling pathway-induced ST-Luc transcription with an IC_50_ value of 9.80 μM. Moreover, elevated ST-Luc activity was significantly decreased by isopenicin A in both SW620 and HCT116 cells (Tang et al., 2019). Kanokmedhakul et al. reported the potent cytotoxic meroterpenoid (chevalone B) with IC_50_ values of 3.9 and 2.9 μg/ml against lung and epidermal carcinoma cells. Chevalones C and D also showed cytotoxic effects with IC_50_ values of 8.7 and 7.8 μg/ml against the BC1 cell line (Kanokmedhakul et al., 2011). Guignardones Q and S isolated from the fungal strain *Guignardia mangiferae* A.J. Roy were reported for their cytotoxic effects against breast cancer cells. However, these compounds showed a weak inhibitory effect on tumor growth (Sun et al., 2015). Terretonin C and rubrolide S, 5-[(3,4-dihydro-2,2-dimethyl-2H-1-benzopyran-6-yl)-methyl]-3-hydroxy-4(4-hydroxyphenyl)-2(5H)-furanone isolated from *Aspergillus terreus* Thom demonstrated potent cytotoxic effects against breast cancer and leukemia cells (Sun et al., 2018). Meroterpenoid periconones E isolated from the fungus *Periconia* reported a cytotoxic effect against breast cancer cells with an IC_50_ value of 4.2 μmol/L (Liu J. M. et al., 2017). Meroterpenoid insuetolides C, (E)-6-(40- hydroxy-20-butenoyl)-strobilactone A, and (E,E)-6-(60,70-dihydroxy-20,40-octadienoyl)-strobilactone A isolated from the ethyl acetate extract of the fungus *Aspergillus insuetus* (Bainier) Thom and Church (1929) inhibited the MOLT-4 cell line proliferation at 50 μg/ml by 51%, 55%, and 72%, respectively (Cohen et al., 2011). Wang et al. also isolated meroterpenoid pestalofones J and reported a weak cytotoxic activity from the fungus *Pestalotiopsos fici* (Wang B. et al., 2016). Recently, two more meroterpenoids (phomeroids A and B) isolated from the fungus *Phomopsis tersa* FS441 reported their cytotoxic effect in various cell lines (SF-268, HepG-2, A549, and MCF-7) (Chen et al., 2020). Andrastin-type meroterpenoids, namely, penimeroterpenoid A, recently isolated from *Penicillium* species, showed a moderate cytotoxic effect against A549, HCT116, and SW480 cell lines (Ren et al., 2021). Tropolactones A, B, and C isolated from the fungus *Aspergillus* reported a cytotoxic potential against human colon carcinoma (HCT-116) with IC_50_ values of 13.2, 10.9, and 13.9 μg/ml (Table 1 and Figure 1).

**TABLE 1:** Sources and biological activity of fungus meroterpenoids.

Source of meroterpenoid	Name of meroterpenoids	Biological activity	References
*Pseudocosmospora* sp. Bm-1-1	Rel-(6′S, 10′R)-Δ^9^ -tetrahydrocannabinolic acid B; rel-(6′S, 10′R)-decarboxy-Δ^9^-tetrahydro cannabinolic acid B	Cytotoxicity	Nakamura et al. (2019)
*Ganoderma cochlear* (Blume & T. Nees) Bres.	(±) Gancochlearols A and B	Cytotoxicity; COX-2 inhibitory	Qin et al. (2018b)
*Ganoderma cochlear* (Blume & T. Nees) Bres.	(±) Cochlearoids N–P	Cytotoxicity, anti-bacterial, BRD4 inhibitors	Qin et al. (2019a)
*Ganoderma cochlear* (Blume & T. Nees) Bres.	Gancochlearols D and C; ganomycin F	Cytotoxicity, N-acetyltransferase	Cheng et al. (2018)
*Ganoderma cochlear* (Blume & T. Nees) Bres.	(+)- and (−)-Spirocochlealactones A–C; ganodilactone	Cytotoxicity, COX2 inhibitors	Qin et al. (2018a)
*Phoma* species	Phomanolides D (2); phomanolide F (4)	Cytotoxicity	Zhang et al. (2019c)
*Ascochyta viciae*	Ascochlorin; 5, 6, 7a, 7b	Cytotoxicity	Quan et al. (2019)
*Neosetophoma* species	Eupenifeldin; dehydroxyeupenifeldin	Cytotoxicity	El-Elimat et al. (2019)
*Stachybotrys chartarum* (Ehrenb.) DSMZ 12880 (chemotype S)	Stachybotrychromens A and B	Cytotoxicity	Jagels et al. (2018)
*Antrodia cinnamomea*	Antroquinonols A, V, W	Cytotoxicity	Chen et al. (2017b)
*Neosartorya spinosa*	1-hydroxychevalone C; 1-acetoxychevalone C; 1,11-dihydroxychevalone C; Quinadoline A	Cytotoxicity	Rajachan et al. (2016)
*Emericella nidulans* HDN12-249	Emeriphenolicins E	Cytotoxicity	Zhou et al. (2016)
*Gliomastix* sp. ZSDS1-F7	Purpurogemutantin, macrophorin A, 4′-oxomacrophorin, 2,3-hydro-deacetoxyyanuthone A, 22-deacetylyanuthone A anicequol	Cytotoxicity; anti-tubercular activity	He et al. (2017a)
*Penicillium* sp. SXH-65	Arisugacins B and F	Cytotoxicity	Sun et al. (2014)
*Xylaria humosa*	Chevalones B and C	Cytotoxicity	Sodngam et al. (2014)
*Penicillium* sp. Sh18	Isopenicin A	Cytotoxicity	Tang et al. (2019)
*Eurotium chevalieri*	Chevalones B, C, and D	Cytotoxicity	Kanokmedhakul et al. (2011)
*Ignardia mangiferae* A348	Guignardones Q and S	Cytotoxicity	Sun et al. (2015)
*Aspergillus terreus Thom* OUCMDZ-2739	Rubrolide S; 5-[(3,4-dihydro-2,2-dimethyl-2H-1-benzopyran-6-yl)-methyl]-3-hydroxy-4(4-hydroxyphenyl)-2(5H)-furanone; terretonin C	Cytotoxicity	Sun et al. (2018)
*Periconia* sp. F-31	Periconones B and E	Cytotoxicity, anti-HIV	Liu et al. (2017a)
*Aspergillus insuetus* (Bainier) Thom & Church (OY-207)	Insuetolides A and C, (E)-6-(40-hydroxy-20-butenoyl)-strobilactone A; strobilactone A, (E,E)-6-(60,70-dihydroxy-20,40-octadienoyl)-strobilactone A	Cytotoxicity, anti-fungal	Cohen et al. (2011)
*Pestalotiopsis fici*	Pestalofones J	Cytotoxicity	Wang et al. (2016a)
*Phoma* sp.	Phomanolide A, eupenifeldin	Anti-proliferative	Zhang et al. (2015)
*Peyronellaea coffeae-arabicae* FT238	11-Dehydroxy epoxyphomalin A	Anti-proliferative	Li et al. (2016b)
*Ganoderma cochlear* (Blume & T. Nees) Bres.	(±)-Cochlearins A–I	Anti-proliferative, anti-oxidant	Peng et al. (2018b)
*Aspergillus terreus*	Terreustoxin C, terretonin	Anti-proliferative	Feng et al. (2019)
*Ganoderma cochlear* (Blume & T. Nees) Bres*.*	(±)-Cochlactones A and B	Anti-inflammation	Peng et al. (2018a)
*Stachybotrys chartarum* (Ehrenb.) 952	Stachybonoids A and F, stachybotrysin C, Stachybotrylactone	Anti-inflammation, anti-viral	Zhang et al. (2017)
*Aspergillus terreus* Thom	Austinoid, 1,2-dehydroterredehydroaustin	Anti-inflammation	Liu et al. (2018b)
*Aspergillus terreus* Thom	Yaminterritrem B	Anti-inflammation	Liaw et al. (2015)
*Talaromyces amestolkine* YX1	Amestolkolide B	Anti-inflammation	Chen et al. (2018)
*Alternaria* sp. JJY-32	Tricycloalternarenes A, B, and C; bicycloalternarenes A, B, C, D, and F; monocycloalternarenes A, B, Cm and D	Anti-inflammation	Zhang et al. (2013)
*Penicillium purpurogenum* MHz 111	Purpurogenolides B, C, and D; berkeleyacetal C	Anti-inflammation	Sun et al. (2016)
*Penicillium brasilianum* WZXY-m122-9	Brasilianoids A–E	Anti-inflammation, dermatological diseases	Zhang et al. (2018a)
*Guignardia mangiferae* A.J. Roy	Mangiterpene C; 2′,3′-seco-manginoid C	Anti-inflammation	Chen et al. (2019)
*Ganoderma theaecolum*	Ganotheaecoloid J	COX-2 inhibitory	Luo et al. (2018b)
*Ganoderma theaecolum*	(±)-Ganotheaecolumols C, D, I, and K; iso-ganotheaecolumol I	COX-2 inhibitory	Luo et al. (2018a)
*H. caput-medusae*	Caputmedusins A, B, and C	α-Glucosidase inhibitors	Chen et al. (2017a)
*Aspergillus terreus* Thom 3.05358	Amauromine B, austalides N	α-Glucosidase inhibitors	Shan et al. (2015)
*Myrothecium* sp. OUCMDZ-2784	Myrothecisins A–D, myrothelactone A, myrothelactone C, tubakialactone B, acremonone G	α-Glucosidase inhibitors	Xu et al. (2018)
*Ganoderma leucocontextum*	Ganoleucins A and C; ganomycins I, B, and C; fornicins C and B	α-Glucosidase inhibitors, HMG-CoA inhibitors	Wang et al. (2017)
*Ganoderma sinense*	Applanatumol I	Anti-oxidant	Gao et al. (2018)
*Ganoderma capensa*	Ganocapensins A and B; ganomycins E, F, I, and C; fornicins E and B	Anti-oxidant	Peng et al. (2016b)
*Perenniporia medulla-panis*	Perennipins A–C, (+)-fornicin A	Anti-oxidant	Kim et al. (2019)
*Phyllosticta* sp. J13-2–12Y	(*S,Z*)-Phenguignardic acid methyl ester	Anti-microbial	Yang et al. (2017)
*Penicillium* sp. T2-8	Preaustinoid D, dihydroxyneogrifolic acid; preaustinoid A1, austin, (S)-18,19-dihydroxyneogrifolin	Antimicrobial, anti-bacterial	Duan et al. (2016)
*Cytospora* spieces	Cytosporolides A–C	Antimicrobial	Li et al. (2010)
Aspergillus sp. TJ23	Spiroaspertrione A, andiconin B	Anti-microbial	He et al. (2017c)
*Ganoderma orbiforme*	Ganoboninone G, ganomycin I	Anti-bacterial	Li et al. (2018d)
*Emericella* sp. TJ29	Emervaridone A	Anti-bacterial	He et al. (2017b)
*Penicillium* sp. SCS-KFD09	Chrodrimanins K and N, verruculides B2, 3-hydroxypentcecilide A	Anti-bacterial, anti-viral	Kong et al. (2017)
*Penicillium citrinum*	Penicimarins G and H, dehydroaustin, 11β-acetoxyisoaustinone, austinol	Anti-bacterial	Huang et al. (2016)
*Dysidea* sp.	Dysidphenols A and C, smenospongimine, smenospongine, smenospongorine, smenospongiarine, smenospongidine	Anti-bacterial	Zhang et al. (2016)
*Aspergillus terreus*	Terreusterpenes A, B, and D	BACE1 inhibitory, AchE inhibitors	Qi et al. (2018b)
*Aspergillus terreus*	Asperterpenes E, F, and J	BACE1 inhibitory	Qi et al. (2018a)
*Aspergillus terreus*	Asperterpenes A and B	BACE1 inhibitory	Qi et al. (2016)
*Aspergillus terreus* Thom	Spiroterreusnoids A–F	BACE1 inhibitory, AchE inhibitory	Qi et al. (2019)
*Ganoderma applanatum*	Applanatumols A and (+) B	Renal fibrosis	Luo et al. (2016)
*Aspergillus* sp. 16-5c	Isoaustinol, dehydroaustin, dehydroaustinol	AchE inhibitors	Long et al. (2017)
*Ganoderma cochlear* (Blume & T. Nees) Bres.	Ganocin D	AchE inhibitors	Peng et al. (2014)
*Ganoderma* species	(+)-Zizhines G, (−)-zizhines G, (−)-ganosinensols A, (+) zizhines P, (−) zizhines P, (+)-zizhines Q, (−) zizhines Q	AchE inhibitors	Luo et al. (2019a)
*Ganoderma capense*	Ganocapenoids C, ganocalidin E, cochlearin I, patchiene A	AchE inhibitors	Liao et al. (2019)
*Penicillium* spices	Arisugacins D, M, O, P, and Q	AchE inhibitors	Dai et al. (2019)
*Verticillium albo-atrum*	Acetoxydehydroaustin A, austin	Activation of sodium channel	Wu et al. (2018)
*Aspergillus aureolatus* HDN14-107	Austalides U and I, merochlorin D, austalide P acid	Anti-viral	Peng et al. (2016a)
*Penicillium funiculosum* GWT2-24	Chrodrimanins A, E, and F	Anti-viral	Zhou et al. (2015)
*Talaromyces* sp. CX11	Talaromyolide D (4)	Anti-viral	Cao et al. (2019)
*Ganoderma lingzhi*	Lingzhilactone B	Renal protective activity	Yan et al. (2015b)
*Ganoderma lingzhi*	Spirolingzhines A, B, C, and D; lingzhines B, D, E, and F; 4-(2,5-dihydroxyphenyl)-4-oxobutanoic acid	Neural stem cell (NSC) proliferation	Yan et al. (2015a)
*Penicilium purpurogenum*	Dhilirolide L	Insecticidal	Centko et al. (2014)
*Penicillium* lividum KMM 4663 and *Penicillium thomii* KMM 4645	Austalide H acid, austalide H acid butyl ester, 13-O-deacetylaustalide I, 13-deacetoxyaustalide I	Inhibition of AP-1	Zhuravleva et al. (2014)
*Endophytic Penicillium brasilianum* found in the *Melia azedarach* root bark	Brasiliamide A	Antimicrobial	Fill et al. (2009)
*Ganoderma lucidum*	Dayaolingzhiols D–E	AchE inhibitors	Luo et al. (2019b)
*Ganoderma austral*	Ganomycin C, (−)-ganoresinain A, ganotheaecoloid G	Neuroprotective activity	Zhang et al. (2019b)
*Ganoderma applanatum*	Spiroapplanatumines G and H	Inhibitors of JAK3	Luo et al. (2017)
*Ganoderma petchii*	Petchiethers A and B	Renal protective activity	Li et al. (2016a)
*Ganoderma petchii*	Petchienes B and (-) D	Increase intracellular free calcium	Gao et al. (2015)
*Ganoderma cochlear* (Blume & T. Nees) Bres.	Cochlearoids F –I, cochlearoid K	Renal protective activity	Wang et al. (2016b)
*Ganoderma cochlear* (Blume & T. Nees) Bres*.*	Cochlearols S, U, X, and Y	Renal protective activity	Wang et al. (2019b)
*Ganoderma cochlear* (Blume & T. Nees) Bres.	Cochlearol K, cochlearin E	Renal protective activity	Wang et al. (2019a)
*Ganoderma cochlear* (Blume & T. Nees) Bres*.*	(+)- and (−)-cochlearols A and B	Renal protective activity	Dou et al. (2014)
*Ganoderma lucidum*	Chizhine F, fornicin B, ganomycin I	Renal protective activity	Luo et al. (2015)
*Ganoderma lucidum*	Lingzhifuran A, lingzhilactone D	Anti-fibrotic activity	Ding et al. (2016b)
*Mangrove endophytic fungus Diaporthe* sp. SCSIO 41011	Chrodrimanins A, B, E, H¸ G, and F	Insecticidal	Luo et al. (2019c)
*Boletinus asiaticus*	Asiaticusinol C, asiachromenic acid, asiaticusin A	BACE1 inhibitory	Yatsu et al. (2019)
*Phyllosticta capitalensis*	Guignardianone C	Phytotoxic activity (plant toxicity)	Ma et al. (2019)

### Cytotoxicity studies of meroterpenoids isolated from marine source

**FIGURE 2:**
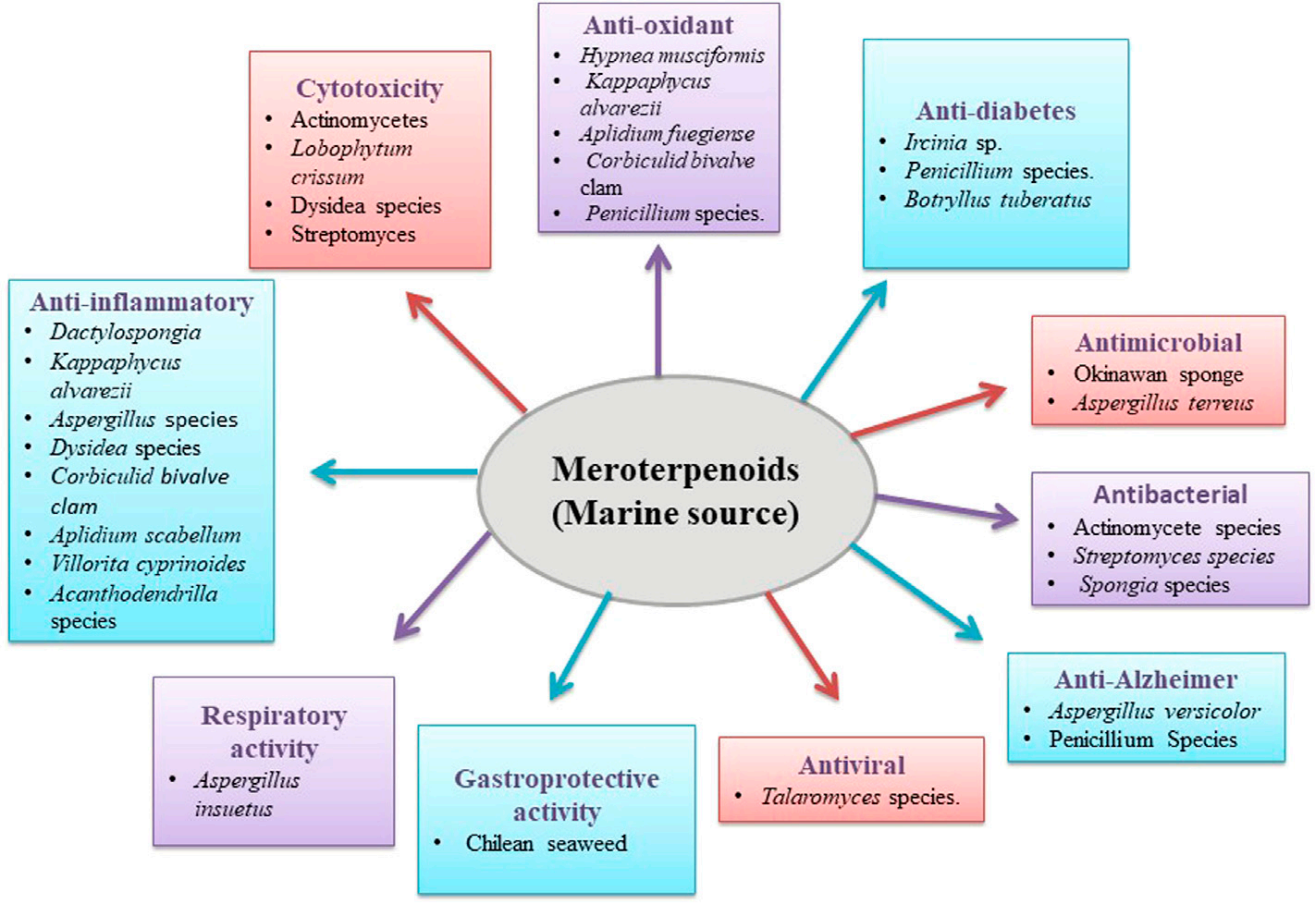
Biological activity of marine meroterpenoids.

Meroterpenoids isolated from marine sources such as *Dactylospongia*, the marine strain of actinomycetes, *Lobophytum crissum* von Marenzeller*,* Dysidea, and *streptomyces* have also been reported for their potential cytotoxic effects. Sesquiterpene and drimane meroterpenoids isolated from *Dactylospongia elegans* (Thiele, 1899) and other species of *Dactylospongia* have been reported as potential cytotoxic agents in various cancer cell lines. Reports show that 19-O-methylpelorol demonstrated a potential cytotoxic effect with an IC_50_ value of 9.2 μM in lung cancer cell lines (PC-9) (Li J. et al., 2018). Yu et al. evaluated the cytotoxic potential of 19-methoxydictyoceratin-A, smenospongiarine, smenospongorine, smenospongimine, and dictyoceratin-C meroterpenoids isolated from *Dactylospongia elegans* (Thiele, 1899) against prostate, pancreatic, and liver cancer cells. They reported that 19-methoxydictyoceratin-A exhibited a moderate activity, whereas smenospongiarine, smenospongorine, smenospongimine, and dictyoceratin-C demonstrated a potent effect with IC_50_ values in the range of 2–37.85 µM in all cancer cell types (Yu et al., 2019). Ebada et al. isolated drimane meroterpenoid metabolites, 5-epi-ilimaquinone, 5-epi-smenospongine, isospongiaquinone, isosmenospongine, and nakijiquinones A and G, from marine sponge *Dactylospongia elegans* (Thiele, 1899), which were assessed for *in vitro* cytotoxicity in mouse lymphoma cells. Results displayed that among the isolated compounds, 5-epi-smenospongidine and isospongiaquinone were the most active with similar IC_50_ values of 1.34 μM in addition to 5-epi-ilimaquinone, isosmenospongine, and nakijiquinones A and G, which showed potent activity (Ebada et al., 2017). A marine strain of actinomycetes has also been reported to contain meroterpenoids with a potent cytotoxic effect. Marinocyanins A and B demonstrated a potent cytotoxic effect against colon cancer cells (Asolkar et al., 2017). Additionally, napyradiomycins 1 to 4 isolated from actinomycete also confirmed a cytotoxic effect *via* cell apoptosis in colon adenocarcinoma cells with an IC_50_ value of around 1 and 2 μM (Farnaes et al., 2014). Cheng et al. also reported the cytotoxic potential of napyradiomycins A and B4 isolated from *Streptomyces* strain with an IC_50_ value between 1 and 5 μg/ml against colon cancer cells (Cheng et al., 2013). The soft coral *Lobophytum crissum* von Marenzeller has also been reported for the presence of potential cytotoxic meroterpenoid, namely, pseuboydone C, cyclo-(Phe-Phe), speradine C, 24,25-dehydro-10,11-dihydro-20-hydroxyaflavinin, and aflavinine, with the IC_50_ mean values of 0.7, 0.8, 0.9, 0.5, and 0.4 μM, respectively, against insect cell line SF9 (Lan et al., 2016). Kim et al. isolated six new drimane sesquiterpene hydroquinone meroterpenoids along with arenarol from *Dysidea* sp. Sponge. The cytotoxic investigations on K562 and A549 cell lines showed that aureol B; melemeleones C and D; cycloaurenones A, B, and C; and arenarol showed cytotoxic activity comparable to doxorubicin and showed an IC_50_ value below 10 μM. It was reported that aureol B and arenarol were the most potent meroterpenoids with a potent cytotoxic effect (Kim et al., 2015). Dysideanones A and B, two meroterpenoids isolated from *Dysidea avara* (Schmidt, 1862), also showed moderate cytotoxic activity against colon cancer cells (Haque et al., 2018). (+)-5-Epi-ethylsmenoquinone isolated from *Smenospongia* was reported as cytotoxic meroterpenoid against two different colon cancer cell lines with IC_50_ values of 3.24 and 2.95 μM (Hwang et al., 2015). Fiorini et al. reported that paniceins B2, B3, and C and particularly panicein A hydroquinone, which is a natural meroterpenoid formed by the mucosa of the Mediterranean sponge *Haliclona (Soestella)*, could inhibit the function of the patched model doxorubicin efflux built from AcrB structure, and *in vitro* melanoma cells cytotoxicity was enhanced by the doxorubicin. Four meroterpenoids, panicein B2, B3, and C and panicein A hydroquinone were tested for cytotoxicity. These meroterpenoids exhibited moderate cytotoxicity above the micromolar range with panicein A hydroquinone inhibiting CCRF-CEM leukemia cells most selectively with a cytostatic effect (TGI) of 25 μM (Fiorini et al., 2015) (Table 2 and Figure 2).

**TABLE 2:** Sources and biological activity of marine meroterpenoids.

Source of meroterpenoid	Name of meroterpenoids	Biological activity	References
*Dactylospongia* sp**.**	Dactylospongins A, B, and D, Ent-melemeleone B, dysidaminone N, 19-O-methylpelorol	Cytotoxicity, Anti-inflammation	Li et al. (2018c)
*Dactylospongia elegans*	19-Methoxy-dictyoceratin-A, smenospongiarine, smenospongorine, smenospongimine, dictyoceratin-C	Cytotoxicity	Yu et al. (2019)
*Dactylospongia elegans*	5-Epi-ilimaquinone, 5-epi-smenospongidine, isospongiaquinone, isosmenospongine, nakijiquinones A and G	Cytotoxicity	Ebada et al. (2017)
*Dysidea* species	Aureol B; melemeleones C and D, cycloaurenones A, B, and C; Arenarol	Cytotoxicity	Kim et al. (2015)
*Dysidea* avara	Dysideanones A and B	Cytotoxicity	Haque et al. (2018)
*Smenospongia aurea* (08FL-20-B), *Smenospongia cerebriformis* (08FL-20)	(+)-5-Epi-ethylsmenoquinone	Cytotoxicity	Hwang et al. (2015)
*Haliclona (Soestella) mucosa*	Panicein A hydroquinone, paniceins B2, B3, and C	Cytotoxicity	Fiorini et al. (2015)
*Dysidea villosa*	Dysivillosins A–D	Anti-inflammation	Jiao et al. (2017)
*Dysidea septosa*	Septosones A and C	Anti-inflammation	Gui et al. (2019)
Okinawan marine sponge (SS-1202)	Nakijiquinone S, nakijinol C	Anti-microbial	Suzuki et al. (2014)
*Spongia* species	Langcoquinpne C, smenospongorine	Anti-bacterial	Nguyen et al. (2017)
*Spongia* spieces	Langcoquinones A and B, dictyoceratin A, ilimaquinone, smenospongine, smenospongidine, nakijiquinone L	Anti-bacterial	Li et al. (2018b)
*Callyspongia* spices	Isoakaterpin	Anti-leishmanial	Gray et al. (2007)
*Dysidea* species	Avinosol, avarone, avarol, avinosone	Anti-invasion activity	Diaz-Marrero et al. (2006)
*Acanthodendrilla* species	(+)-Makassaric acid, (+)-subersic acid	Inhibitors of protein kinase MK2	Williams et al. (2004)
*Actinomycete* strains CNS-284 and CNY-960	Marinocyanins A and B	Cytotoxicity	Asolkar et al. (2017)
*Actinomycete* species	Napyradiomycins 1–4	Cytotoxicity	Farnaes et al. (2014)
*Streptomyces* strains	Napyradiomycins A and B4	Cytotoxicity	Cheng et al. (2013)
MAR 4 *Streptomyces* Strains	Napyradiomycins A and B3	Anti-microbial	Cheng et al. (2013)
*Streptomyces* sp.	Merochlorins E and F	Anti-bacterial	Ryu et al. (2019)
*Streptomyces* sp. strain CNQ-525	A80915A, A80915B	Anti-bacterial	Haste et al. (2011)
*Kappaphycus alvarezii* (Doty) Doty ex Silva (family Solieriaceae)	2-Ethyl-6-(4-methoxy-2-((2-oxotetrahydro-2Hpyran-4-yl) methyl) butoxy)-6-oxohexyl 5-ethyloct-4-enoate (C29)	Anti-inflammation Antioxidant	Makkar and Chakraborty, (2018)
*Stypopodium flabelliforme*	Sargaol, epitaondiol, stypodiol, isoepitaondiol	Gastroprotective	Areche et al. (2015)
*Aspergillus* sp. ZL0-1b14	Aspertetranones A–D	Anti-inflammation	Wang et al. (2015b)
*Penicillium* sp. YPGA11	Conidiogenone C	Anti-oxidant	Cheng et al. (2019)
*Aspergillus terreus* Thom EN-539	Aperterpenes N, terretonin G	Anti-microbial	Li et al. (2019b)
*Aspergillus terreus*	(22E,24R)-Stigmasta-5,7,22-trien-3-b-ol, stigmast-4-ene-3-one, aspernolides F	Anti-microbial, anti-leishmanial	Ibrahim et al. (2015)
*Aspergillus versicolor*	Asperversins G	AchE inhibitors	Li et al. (2018b)
*Penicillium* sp. SK5GW1L	3-Epiarigsugacin E, arisugacin B, territrem C, terreulactone C	AchE inhibitors	Ding et al. (2016a)
*Penicillium* sp. SF-5497	Preaustinoid A6, berkeleyone C	PTP1B inhibitors	Park et al. (2019)
*Aspergillus insuetus*	Terretonins E and F, aurantiamine	Mammalian mitochondrial respiratory chain Inhibitors	López-Gresa et al. (2009)
*Corbiculid bivalve* clam and *Villorita cyprinoides*	Dihydro-5-(8-(9,12-dihydro-8-methyl-11-propyl-2H-pyran-8-yl)-ethyl) furan-2(3H)-one; tetrahydro-3-methoxy-5-((E)-8,12-dimethyloct-8 enyl)-pyran-2-one; (12E)-(3,4,6,7,8,8a-hexahydro-1H-isochromen-3-yl)-methyl-hept-12-enoate; (10E)-butyl-9-(6-ethyl-3,4,6,7,8,8a-hexahydro-1H-isochromen-3-yl)-pent-10-enoate	Anti-inflammation; COX2 inhibition; Anti-oxidant	Joy and Chakraborty (2018)
Ascidian *Aplidium scabellum*, 322	2-Geranyl-6-methoxy-1,4- hydroquinone-4-sulfate, scabellone B, 8-methoxy-2-methyl-2-(4-methyl-3-pentenyl)-2H-1-benzo-pyran- 6-ol, 2-geranyl-6-methoxy-1,4-hydro- quinone	Anti-inflammatory, anti-plasmoid activity	Chan et al. (2011)
Antarctic Ascidian, *Aplidium* species	Rossinones A and B	Anti-oxidant	Appleton et al. (2009)
*Botryllus tuberatus*	Tuberatolides A and B, 2′-*epi*-tuberatolide B, yezoquinolide (*R*)-sargachromenol, (*S*)-sargachromenol	Human farnesoid X receptor (Hfxr), activated chenodeoxycholic acid (CDCA)	Choi et al. (2011)
*Dysidea* species	(+)-Yahazunone, (+)-chromazonarol	Anti-fungal	Zhang et al. (2018b)
*Cystoseira baccata*	(3R)- and (3S)-tetraprenyltoluquinol; (3R)- and (3S)-tetraprenyltoluquinone	Anti-leishmanial	Bruno de Sousa et al. (2017)
*Lobophytum crissum*, 200	Pseuboydone C; cyclo-(Phe-Phe), speradine C; aflavinine; 24,25-dehydro-10,11-dihydro-20-hydro-xyaflavinin	Cytotoxicity	Lan et al. (2016)

### Cytotoxicity studies of meroterpenoids isolated from plants

Herbal plants are also one of the major sources of different types of meroterpenoids with cytotoxic activity. Plants from approximately 12–13 different genera, such as *Lycium barbarum L.*, *Psidium*, *Eucalyptus*, *Arnebia*, *Baeckea*, *Pogostemon*, *Eugenia*, *Euphorbia*, *Rhododendron*, *Belamcanda*, *Myrtus*, *Rhodomyrtus*, *Calocedrus*, and *Callistemon*, have been reported to date to possess cytotoxic meroterpenoids in their different parts.

**FIGURE 3:**
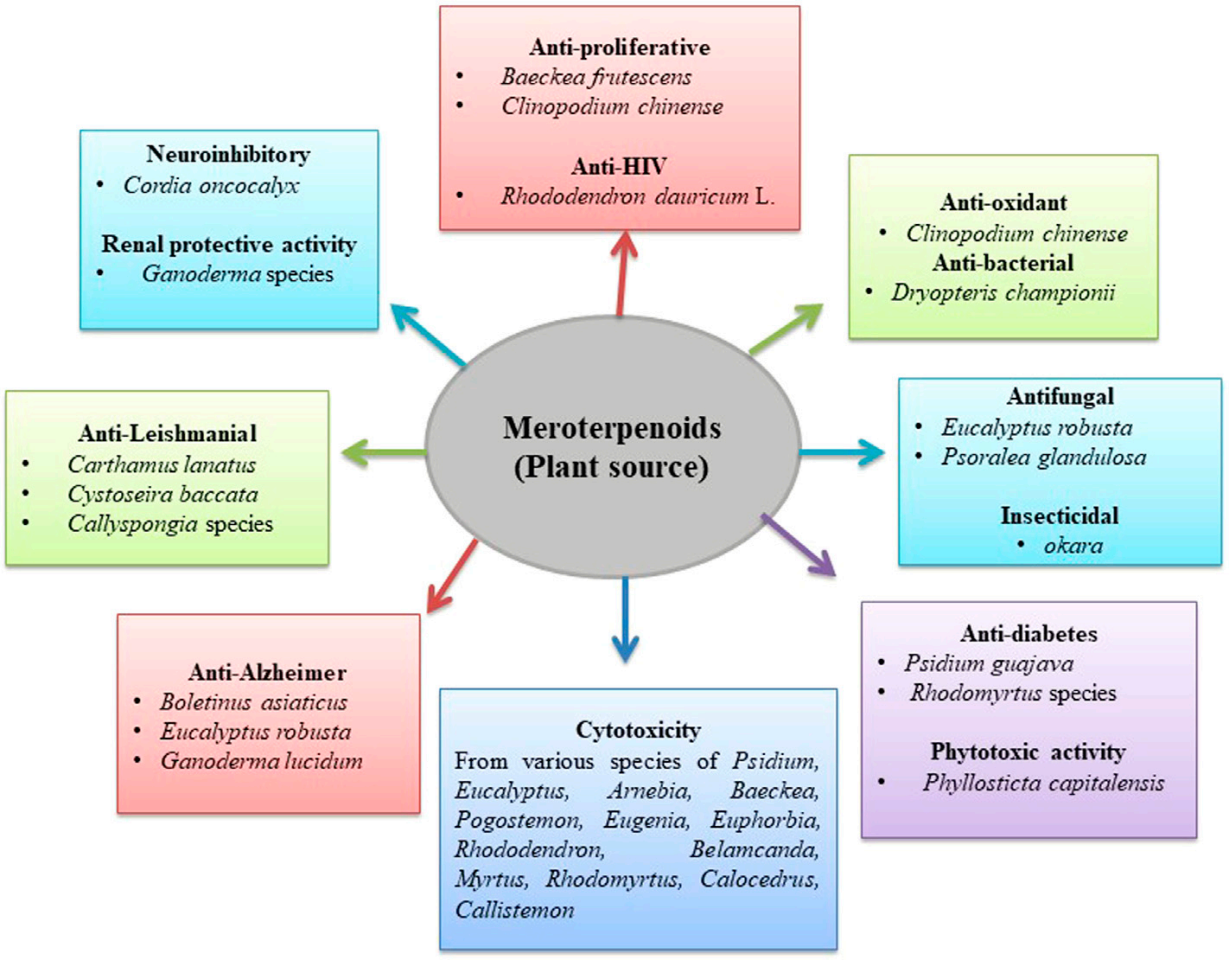
Biological activity of plant meroterpenoids.

The tetracyclic meroterpenoid, namely, bipolahydroquinones C, cochlioquinones I-M, and cochlioquinones D, isolated from the fungus *Lycium barbarum* L*.* demonstrated a cytotoxic effect against breast cancer (MDA-MB-231) cell line and squamous cell carcinoma (NCI-H226). The results suggested that meroterpenoids from this species showed a cytotoxic effect in both cell lines. Bipolahydroquinones C and cochlioquinone D showed significant effects with IC_50_ values of 5.5 and 6.9 μM against squamous cell carcinoma cells, respectively. Cochlioquinones I-M were reported to have an IC_50_ value of more than 10 μM against squamous cell carcinoma cells. Similarly, significant inhibition was shown against breast cancer cells by cochlioquinone K (IC_50_ 9.5 μM), bipolahydroquinone C (IC_50_ 6.7 μM), cochlioquinone I (IC_50_ 8.5 μM), cochlioquinone L (IC_50_ 7.5 μM), and cochlioquinone M (IC_50_ 5.6 μM) (Long et al., 2019). Two species of *Psidium* were reported to have cytotoxic meroterpenoids in their leaves. Four sesquiterpene-based meroterpenoid (i.e., psiguadials A, B, C, and D) and monoterpene-based meroterpenoid (guadials C) isolated from *Psidium guajava* L. demonstrated a cytotoxic effect against two hepatic cancer cell line. Psiguadials A, B, C, and D confirmed a potent effect with IC_50_ values below 1 μM against HepG2. However, guadial C and psiguadials A and B showed moderate cytotoxic effects against HepG2/ADM cells (Shao et al., 2010, 2012; Jian et al., 2015). Guajadial, a dialdehyde meroterpenoid, demonstrated a potent cytotoxic effect with an IC_50_ value less than that of the standard drug cisplatin against A549 and H1650 cell lines (Wang et al., 2018a). Other meroterpenoids, namely, guajavadials A–C isolated from *Psidium guajava* L. showed moderate activity against five human cell lines (HL-60, A-549, SMMC-7721, MCF-7s, and SW480), with guajavadial C being the most effective with an IC_50_ value of 3.54 μM toward SMMC-7721 cell lines (Qin et al., 2016). Additionally, meroterpenoids, such as 4,5-diepipsidial A and guajadial B, were also isolated from *Psidium guajava* L*.* with a weak cytotoxic potential (Qin et al., 2017c). Littordials B, C, and E, formyl phloroglucinol-β-caryophyllene meroterpenoids isolated from *Psidium littorale* Raddi, were active against the MDA-MB-321 cell line, whereas littordials C and E were reported as active compounds against the murine model for human melanoma cells and human lung cancer cells, respectively (Xu et al., 2019). Qin et al. isolated cytotoxic formyl phloroglucinol-terpene meroterpenoid eucalypglobulusal F from *Eucalyptus globulus* Labill. fruits, which demonstrated a potent action with an IC_50_ value of 3.3 μM against T lymphoblastoid cells (Qin et al., 2018e). Three more formyl phloroglucinol meroterpenoids (eucalteretials C, euglobal IX, and euglobal Ib) isolated from the twigs and leaves of *Eucalyptus tereticorni* Sm. by Liu et al. exhibited cytotoxic potential in different cancer cells. Eucalteretial C and euglobal IX were significantly toxic with IC_50_ values of 4.8 and 9.5 μM against HCT116 cells, whereas euglobal Ib was active against DU145 cells with an IC_50_ value of 7.8 μM (Liu H. et al., 2018). *Eucalyptus robusta* Sm. leaves also showed the presence of formyl phloroglucinol meroterpenoid eucalrobusone C with a cytotoxic effect against liver, breast, and bone cancer cells (Shang et al., 2016a). In a similar study, eucalrobusone C demonstrated a cytotoxic effect against liver cancer cells through p38 MAPK pathway-induced apoptosis (Jian et al., 2017). From the roots of *Arnebia euchrome* (Royle) Johnston, thirteen meroterpenoids have been isolated with cytotoxic potential. Arnebinone B and 6S,11Z-2-methoxy-arnebinone B demonstrated a cytotoxic effect against different liver cancer cells. 6S,11Z-2-Methoxy-arnebinone B exhibited the most potent activity against SMMC-7721, HepG2, QGY-7703, and HepG2/ADM human liver cancer cell lines, whereas arnebinone B exhibited moderate growth inhibitory effects against HepG2/ADM (Wang et al., 2018b). Furthermore, arnebinols A and C, 8-O-dimethyl-11-deoxyalkannin, arnebinone B, clavilactone A, and shikonofurans A, B, and C isolated from the roots of the same species confirmed potent cytotoxic effect against osteosarcoma. However, deoxyalkannin, arnebinone, and shikonofuran A demonstrated strong inhibition against human liver cancer cells (Wang L. et al., 2015). Xu-Jie Qin isolated polymethylated phloroglucinol meroterpenoids (baeckfrutones (-)-B, F, and K) from the leaves and twigs of *Baeckea frutescens* Linnaeus, which exhibited a remarkable activity with IC_50_ values of 1.33, 15.61, and 12.89 μM against human prostate, lung, and colon cancer cells, respectively (Qin et al., 2018f). Nguyen et al. isolated pyrone-sesquiterpenoid meroterpenoids pogostemins A, B, and C from the aerial parts of *Pogostemon auricularius* (L.) Hassk., reporting cytotoxicity against the lung cancer cells, keratin forming tumor cell line, liver, gastric cancer, and colorectal adenocarcinoma cells. The study concluded that pogostemins A showed a potent cytotoxic effect, and pogostemins B and C exhibited a moderate effect against the tested cell lines (Nguyen et al., 2018). Eugenials C, D, and E isolated from the fruit extract of *Eugenia umbelliflora* O. Berg showed cytotoxic potential against myelogenous leukemia and murine melanoma cell (Farias et al., 2018). Rubiginosins A, D, and G and anthopogochromene B, isolated from the flowers of *Rhododendron rubiginosum* Franch. var. *rubiginosum* showed a moderate cytotoxic effect against hepatic and leukemia cells (Yang et al., 2018). Similarly, four meroterpenoids (belamcanoxide A, iridobelamal A, isoiridogermanal, and iridal) isolated from rhizomes of *Belamcanda chinensis* (L.) DC. showed a moderate cytotoxic effect against liver and stomach cancer cells (Ni et al., 2017). Liu et al. isolated meroterpenoids rhodomentones A and B from the *Rhodomyrtus tomentosa* (Aiton) Hassk. leaves, showing a moderate cytotoxic effect (Liu H. X. et al., 2016). Saleh et al. isolated the xanthomonic acid from the mango pathogenic organism *Xanthomonas citri* (Hasse, 1915)*,* which has been reported to show a cytotoxic effect *via* the induction of autophagy. Furthermore, it showed potential effect against embryonic kidney, cervical, and breast cancer cell lines, with higher selectivity toward estrogen-independent breast cancer cells (MDA-MB-231) compared to the estrogen-dependent type (MCF-7) (Saleh et al., 2016). Hsieh et al. isolated secoabietane-type diterpenoid meroterpenoid ferrugimenthenol from the bark of *Calocedrus macrolepis* Kurz var. *formosana*. Results of the study indicated that ferrugimenthenol displayed potent activity against human oral epidermoid carcinoma cells (Hsieh et al., 2011). Qin et al. isolated myrtucommulone D, isomyrtucommulone B, and callisalignenes G–I from the *Callistemon salignus* leaves and twigs. Myrtucommulone D, isomyrtucommulone B, callisalignene G, and H were reported to have potent inhibitory activity. However, callisalignenes I showed a cytotoxic effect against human colon cancer cells. Additionally, callisalignenes G and I displayed cytotoxicity against lung cancer cells, which was more potent than the standard drug VP-16 (Qin et al., 2017a; 2017b). Zhang et al. isolated fischernolides B and D from *Euphorbia fischeriana* Steud. with cytotoxic activity against hepatic, colon, lung, breast, and cervical cancer cell lines. It has been reported that fischernolide B demonstrates a cytotoxic effect by the induction of apoptosis through caspase activation (Zhang et al., 2019a) (Table 3 and Figure 3).

**TABLE 3:** Sources and biological activity of plant meroterpenoids.

Source of meroterpenoid	Name of meroterpenoids	Biological activity	References
*Lycium Barbarum*	Bipolahydroquinone C, cochlioquinone I, cochlioquinone J, cochlioquinone K, cochlioquinone L, cochlioquinone M, cochlioquinone D	Cytotoxicity	Long et al. (2019)
*Psidium guajava* L.	Psiguadials A and B, guajadial	Cytotoxicity, anti-proliferative	Shao et al. (2010)
*Psidium guajava* L.	Guadial C	Cytotoxicity	Jian et al. (2015)
*Psidium guajava* L.	Guajadial	Cytotoxicity	Wang et al. (2018a)
*Psidium guajava* L.	Guajavadials A–C	Cytotoxicity	Qin et al. (2016)
*Psidium guajava* L*.*	4,5-Diepipsidial A, guajadial B	Cytotoxicity, anti-tumor	Qin et al. (2017c)
*Psidium littorale*	Littordials B, C, and E	Cytotoxicity	Xu et al. (2019)
*Eucalyptus globulus*	Eucalypglobulusal F	Cytotoxicity	(Qin et al., 2018e)
*Eucalyptus tereticorni*	Eucalteretial C, euglobals IX and Ib	Cytotoxicity	Liu et al. (2018a)
*Eucalyptus robusta*	Eucalrobusone C	Cytotoxicity	Shang et al. (2016a)
*Arnebia euchroma*	Arnebinone B, 6S,11Z-2-methoxy-arnebinone B	Cytotoxicity	Wang et al. (2018b)
*Arnebia euchroma*	Arnebinols A and C, 8-odimethyl-11-deoxyalkannin, arnebinone B, clavilactone A, shikonofurans A, B, and C	Cytotoxicity	Wang et al. (2015a)
*Baeckea frutescent*	Baeckfrutones (-)-B, F, G, (+) I, J, and K	Cytotoxicity, anti-inflammation	Qin et al. (2018f)
*Pogostemon auricularius*	Pogostemins A–C	Cytotoxicity	Nguyen et al. (2018)
*Eugenia umbelliflora* fruits	Eugenials C, D, and E	Cytotoxicity	Farias et al. (2018)
*Rhododendron rubiginosum* Franch*.*	Rubiginosins A, D, and G, anthopogochromene B	Cytotoxicity	Yang et al. (2018)
*Rhododendron dauricum* L.	Daurichromenic acid (DCA)	Anti-HIV	Saeki et al. (2018)
*Belamcanda chinensis*	Belamcanoxide A, iridobelamal A, isoiridogermanal, iridal	Cytotoxicity	Ni et al. (2017)
*Rhodomyrtus tomentosa*	Rhodomentones A and B	Cytotoxicity	Liu et al. (2016a)
*Calocedrus macrolepis* var. *Formosana*	Ferrugimenthenol	Cytotoxicity	Hsieh et al. (2011)
*Callistemon salignus*	Isomyrtucommulone B, callisalignones A, 2,6-dihydroxy-4-methoxy-3-methylisopropiophenone, 2,6-dihydroxy-4-methoxyisovalerophenone, myrtucommulone	Cytotoxicity; anti-microbial	Qin et al. (2017a)
*Callistemon salignus*	Callisalignenes G, H, and I	Cytotoxicity	Qin et al. (2017b)
*Euphorbia fischeriana*	Fischernolides B and D	Cytotoxicity	Zhang et al. (2019c)
*Baeckea frutescens*	Baefrutones A–D	Anti-inflammation	Hou et al. (2018)
*Baeckea frutescens*	Baeckfrutones (+) N, baeckfrutones S	Anti-inflammation	Zhi et al. (2018)
*Baeckea frutescens*	Baeckfrutones F, G, (+) I, and J	Anti-inflammation	(Qin et al., 2018f)
*Clinopodium chinense* (Benth.) O. Kuntze	Clinoposides G and H	Anti-inflammation, Aanti-oxidant	Zhu et al. (2018)
*Baeckea frutescens*	Frutescones O	Anti-inflammation	Hou et al. (2017)
*Hypericum yojiroanum*	Yojironin A	Anti-microbial	Mamemura et al. (2011)
*Dryopteris championii*	Aspidin BB, desaspidin BB, Ddesaspidin PB	Anti-bacterial	Chen et al. (2016)
*Eugenia umbelliflora* O. Berg	Eugenials C and D	Anti-bacterial	Li et al. (2018b)
*Eucalyptus robusta*	Eucalrobusones T, U, and (+) X	Anti-fungal	Shang et al. (2019)
*Eucalyptus robusta*	Eucalrobusones J and O	Anti-fungal	Shang et al. (2016b)
*Psoralea glandulosa*	Bakuchiol, 3-hydroxy-bakuchiol	Anti-fungal	Madrid et al. (2012)
*Eucalyptus robusta*	Eucalyptus dimer A, eucalyprobusone A	AchE inhibitors	Qin et al. (2018d)
*Rhodomyrtus tomentosa*	Rhodomyrtusials A and B, tomentodiones Q	AchE inhibitors	Qin et al. (2019b)
*Magnolia officinalis* var. *biloba*	Magterpenoids A and C	PTP1B inhibitors	Li et al. (2018a)
*Rhododendron capitatum*	(−)- and (+)-Rhodonoid B	PTP1B inhibitors	Liao et al. (2015)
*Rhododendron nyingchiense*	Nyingchinoids (+)A, (+)B, (-)C, (-)D and (+/-)H, grifolin	PTP1B inhibitors	Huang et al. (2018)
*Magnolia officinalis* var.* biloba*	Magmenthanes E and H	PTP1B inhibitors	Li et al. (2019a)
*Hypericum japonicum*	Japonicols E and H	Anti-KSHS activities	Hu et al. (2018)
*Rhododendron capitatum*	(+)-Rhodonoid C	Anti-viral	Liao et al. (2017)
*Hypericum japonicum*	Hyperjaponols B and D	Anti-viral	Hu et al. (2016)
*Cordia oncocalyx*	rel-1,4,8α-Trihydroxy-5-furanyl-2-methoxy-8aβ-methyl-6,7,8, 8a,9,10-hexahydro-10-anthracenone; 6- formyl-2-methoxy-9-methyl-1,4-phenanthrendione, rel-10β,11β- epoxy-11β-ethoxy-8α-hydroxy-2-methoxy-8aβ-methyl- 5α,6,7,8,8a,9,10aβ-octahydro-1,4-anthracendione	Neuroinhibitory	Matos et al. (2017)
*Melaleuca Leucadendron* L.	Melaleucadines A and B	Neuroprotective activity	Xie et al. (2019)
*Clinopodium chinense*	Clinoposides B, D, and F	Cardioprotective activity	Zhu et al. (2016)
*Okara* fermented with *Talaromyces* sp. strain YO-2.	Chondrimanins D–F	Insecticidal	Hayashi et al. (2012)
*Psoralea corylifolia* L*.*	Bakuchiols, acetylbakuchiol, O-methyl, and O-ethyl bakuchiols	Hypoxia-inducible factor-1 (HIF-1) inhibitory	Wu et al. (2008)
*P. corylifolia*	(S)-Bakuchiol	Hypoxia-inducible factor-1 (HIF-1) inhibitory	Wu et al. (2007)
*Eucalyptus robusta*	Eucarobustol E (EE)	Anti-biofilm activity	Liu et al. (2017b)
*Psidium guajava* L*.*	Psiguajadials A–L, guajavadials A and C, psiguadials A and D, guapsidial A, psidial A, guajadial, guajadials C–F, guadial A	Phosphodiesterase-4 inhibitors	Tang et al. (2017)

### Cytotoxicity studies of meroterpenoids isolated from algae

Meroterpenoids of different types isolated from various algal species such as *Sargassum* and *Cystoseira* were tested against various cancer cell lines and reported cytotoxic activity.

**FIGURE 4:**
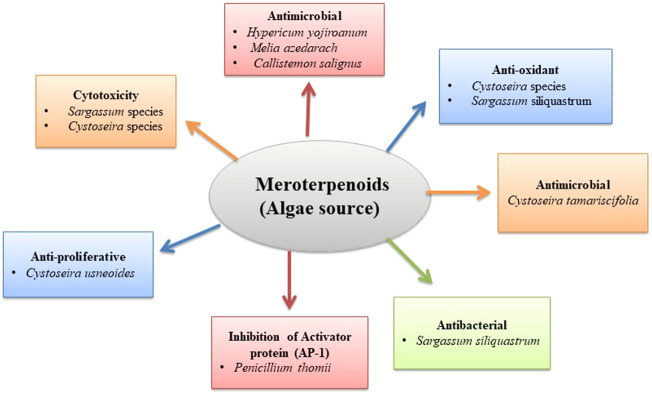
Biological activity of algae meroterpenoids.

Meroterpenoids isolated from two genera of brown algae have been reported for their cytotoxic effects in various cancer cell lines. Lee et al. isolated sargachromanols J, Q, and R, from *Sargassum* algae, which reported potential cytotoxic effects against human gastric, colon, and fibrosarcoma cancer cell lines with IC_50_ values of 6.5 μg/ml (sargachromanol J), 3.4 μg/ml (sargachromanol Q), and 13.9 μg/ml (sargachromanol R), respectively (Lee et al., 2014). They also isolated sargachromanols E, D, and P meroterpenoids from *Sargassum siliquastrum* (Mertens ex Turner) C. Agardh, 1820. All compounds were tested for their cytotoxic potency against human gastric, colon, fibrosarcoma, and breast cancer cell lines. The results indicated that sargachromanols E, D, and P displayed potent cytotoxicity in AGS cell lines (IC_50_ values of 0.7, 6.1, and 0.7 μg/ml), HT-29 (IC_50_ values of 0.5, 1.0, and 3.3 μg/ml), and HT-1080 cell lines (IC_50_ values of 5.7, 0.8, and 1.8 μg/ml), respectively (Lee et al., 2013). Six new tetraprenyltoluquinol derivatives, two triprenyltoluquinol derivatives, and two new tetraprenyltoluquinone derivatives, 2-[(2′E,6′Z,10′E, 14′Z)-5′-Oxo-15′-hydroxymethyl-3′,7′,11′-trimethylhexadeca-2′,6′,10′,14′-tetraenyl]-6-methylhydroquinone, 2-[(2′E,6′E,10′E, 14′Z)-5′-Oxo-15′-hydroxymethyl-3′,7′,11′-trimethylhexadeca-2′,6′,10′,14′-tetraenyl]-6-methylhydroquinone, 5-oxoisocystofuranoquinol 2-[(2′E,6′E,10′E, 14′Z)-5′-hydroxy-15′-hydroxym-ethyl-3′,7′,11′-trimethylhexadeca-2′,6′,10′,14′-tetraenyl]-6-methylhydroquinone and 5-oxocystofuranoquinol, were isolated from the brown algae *Cystoseira crinite* Duby, 1830, with moderate cytotoxic activity toward gastric, hepatic, and breast cancer cells (Fisch et al., 2003) (Table 4 and Figure 4).

**TABLE 4:** Sources and biological activity of algae meroterpenoids.

Source of meroterpenoid	Name of meroterpenoids	Biological activity	References
*Sargassum*	Sargachromanols J, Q, and Ra	Cytotoxicity	Lee et al. (2014)
*Sargassum siliquastrum*	Sargachromanols E, D, and P	Cytotoxicity	Lee et al. (2013)
*Cystoseira crinita* Duby	2-[(2′E,6′E,10′E,14′Z)-5′-Oxo-15′-hydroxymethyl-3′,7′,11′- trimethylhexadeca-2′,6′,10′,14′-tetraenyl]-6-methylhyd- roquinone	Cytotoxicity, anti-oxidant	Fisch et al. (2003)
	2-[(2′E,6′Z,10′E,14′Z)-5′-Oxo-15′-hydroxymethyl-3′,7′,11′- trimethylhexadeca-2′,6′,10′,14′-tetraenyl]-6-methylhyd- roquinone		
	2-[(2′E,6′E,10′E)-5′-Oxo-13′-hydroxy-3′,7′,11′,15′-tetra- methylhexadeca-2′,6′,10′,14′-tetraenyl]-6-methyl hydroquinone		
	2-[(2′E,6′Z,10′E)-5′-Oxo-13′-hydroxy-3′,7′,11′,15′-tetra- methylhexadeca-2′,6′,10′,14′-tetraenyl]-6-methyl hydroquinone		
	2-[(2′E,6′E,10′E)-5′-Oxo-3′,7′,11′,15′-tetramethyl hexadeca- 2,6,10′,14′-tetraenyl]-6-methyl hydroquinone		
	2-[(2′E,6′Z,10′E)-5′-Oxo-3′,7′,11′,15′-tetramethyl hexadeca-2′,6′,10′,14′-tetraenyl]-6-methyl hydroquinone,		
	2-[(2′E,6′E)-5′-Oxo-3′,7′,11′-trimethyldodeca-2′,6′10′-trie-nyl]-6-methyl hydroquinone		
	2-[(2′E,6′Z)-5′-Oxo-3′,7′,11′-trimethyldodeca-2′,6′,10′-trie- nyl]-6-methyl hydroquinone		
	5-Oxo-cystofuranoquinol		
	5-Oxo-isocysto furanoquinol		
	2-[(2′E,6′E,10′E)-5′,13′-dioxo-3′,7′,11′,15′-tetrameth- ylhexadeca-2′,6′,10′,14′-tetraenyl]-6-methyl hydroquinone		
	2[(2′E,6′E,10′E, 14′Z)-5′-Hydroxy-15′-hydroxym- ethyl-3′,7′,11′-trimethylhexadeca-2′,6′,10′,14′-tetraenyl]-6- methyl hydro quinone		
*Cystoseira usneoids*	11-Hydroxy-11-O-methylamentadione (AMT-E)	Anti-inflammation	Zbakh et al. (2016)
*Cystoseira usneoides*	Cystodione A and B, Amentadione-1′-methyl ether, 6-cis-Amentadione-1′-methyl ether, Usneoidone Z, 11-Hydroxyamentadione-1′-methyl ether	Anti-inflammation, anti-oxidant	De Los Reyes et al. (2013)
*Sargassum siliquastrum*	Sargachromanols S and T	Anti-oxidant	Kang and Kim (2017)
*Sargassum siliquastrum*	Sargachromanols A–P	Anti-oxidant	Jang et al. (2005)
*Cystoseira tamariscifolia*	Cystophloroketals A–D	Anti-microbial	El Hattab et al. (2015)
*Sargassum siliquastrum* and *C. albicans*	Sargachromanols D, F, H, L, M, and P	Anti-bacterial inhibitors of Na+/K + ATPase, isocitrate lyase (ICL) inhibitors	Chung et al. (2011)
*Sargassum serratifolium*	Sargahydroquinoic acid, sargachromanol, sargaquinoic acid	BACE1 inhibitory, AchE inhibitory	Seong et al. (2017)

### Anti-proliferative activity of meroterpenoids

#### Anti-proliferative activity of meroterpenoids isolated from the fungus

Meroterpenoids isolated from various fungus species, such as *Phoma, Peyronellaea coffeae-arabicae FT238*, and *Aspergillus terreus* Thom, have been studied for their anti-proliferative activity against various cancer cells. Reports reveal that phomanolide A and eupenifeldin isolated from the fermentation cultures of solid substrate fungus *Phoma* sp. eupenifeldin effectively inhibited the proliferation of neuroblastoma, glioblastoma, and neuroglioma cells. Similarly, phomanolide A reported an inhibitory effect with an IC_50_ value of 81.1 μM against the neuroblastoma cells. In addition, phomanolide A demonstrated an anti-proliferative effect with an IC_50_ value of 14.3 μM only on cervical cancer cells (HeLa), comparable to that of cisplatin (Zhang et al., 2015). Li et al. isolated meroterpenoid 11-dehydroxy epoxyphomalin A from fungus *Peyronellaea coffeae-arabicae* FT238, showing inhibitory activity against OVCAR3 (mt-p53R248) with an IC_50_ value of 0.5 μM. Furthermore, Stat3 strongly at 5 μM (Li C. S. et al., 2016) (±)-cochlearin D isolated from *Ganoderma cochlear* (Blume & T. Nees) Bres*.* demonstrated anti-proliferative activity when tested on HSC-T6 cells through inhibition of TGF-β1-induced HSCs proliferation. However, the non-toxic, effective concentration of (±)-cochlearin D has a weak inhibitory effect on TGF-β1 and thus demonstrates a weak anti-proliferative effect (Peng X. et al., 2018). Feng et al. isolated highly oxygenated meroterpenoids from *Aspergillus terreus* Thom (the Antarctic fungus), namely, terreustoxin C and terretonin. The isolated compounds were tested for concanavalin A- (Con A-) induced T-cell proliferation for *in vitro* immunomodulation. It was found that compounds significantly inhibited murine Con A-induced T-cell proliferation at the concentration of 10 μM (Feng et al., 2019). Novel sesquiterpenoid diphenylmethane meroterpenoids (psiguadials A and B) along with a pair of known epimer guajadial isolated from the leaves of *Psidium guajava* L. also showed moderate inhibitory activity against hepatocellular carcinoma cells (Shao et al., 2010) (Table 1 and Figure 1).

### Anti-inflammatory activity of meroterpenoids

#### Anti-inflammatory activity of meroterpenoid isolated from the fungus

Meroterpenoids isolated from different natural sources have been extensively studied as anti-inflammatory agents. In order to study the anti-inflammatory effect of meroterpenoids, these compounds were tested on RAW 264.7-induced lipopolysaccharide (LPS) macrophage cells. These cells exhibited increased production of NO, TNF-alpha and other inflammatory parameters. If meroterpenoids could decrease the production of these parameters, it meant that they have the potential to be used for anti-inflammatory effects.

Polycyclic-meroterpenoid (±)-cochlactones A and B and their isomers isolated from *Ganoderma cochlear* (Blume & T. Nees) Bres. reported a stronger inhibitory effect on NO production (Peng X.-R. et al., 2018). Polyketide-terpenoid hybrid meroterpenoids, stachybonoids C and F and stachybrotrylactone, isolated from the fungus *Stachybotrys chartarum* (Ehrenb.), displayed moderate inhibitory activity on NO production (Zhang et al., 2017). Meroterpenoids austinoid and 1,2-dehydroterredehydroaustin isolated by Liu et al. from the *Aspergillus terreus* Thom mangrove endophytic fungus showed weak inhibitory action toward the NO production (Liu Z. et al., 2018). Yaminterritrems B, isolated by Liaw et al. from *Aspergillus terreus* Thom with the EC_50_ value at 18.3 μM, demonstrated a reduction in the expression of COX-2-induced LPS at the protein and RNA levels (Liaw et al., 2015). Meroterpenoid amestolkolide B isolated from mangrove endophytic fungus *Talaromyces amestolkiae* Yilmaz, Houbraken, Frisvad & Samson 2012 displayed potent inhibitory activity by inhibiting RAW264.7 cells activated lipopolysaccharide NO production (Chen et al., 2018). The NF-κB inhibitory activity of tricycloalternarene A; bicycloalternarenes A, B, C, D, and F; tricycloalternarenes B and C; monocycloalternarenes A, B, C, and D; and hydrogenated cyclopenta[b]chromans isolated from the *Alternaria* sp. JJY-32 sponge-associated fungus was tested, and all compounds showed activity in RAW264.7 cells with IC_50_ values between 39 and 85 μM (Zhang et al., 2013). Jing Sun et al*.* isolated purpurogenolides B–D and berkeleyacetal C from *Penicillium purpurogenum* Stoll. (1923) MHz 111. These exhibited inhibition activity with IC_50_ values of 30.0, 15.5, and 0.8 μM against NO production (Sun et al., 2016). A study on fungus *Penicillium brasilianum* Bat. by Zhang et al. led to the isolation of 3,5-dimethylorsellinic acid- (DMOA-) based meroterpenoids, brasilianoids A, B, and C. Brasilianoids A exhibited stimulation of filaggrin and caspase-14 expression in a dose-dependent manner in HaCaT cells, whereas brasilianoids B and C caused moderate inhibition of RAW 264.7 macrophages LPS-induced NO production (Zhang J. et al., 2018). Mangiterpenes C and 2′,3′-secomanginoid C isolated from *Guignardia mangiferae* A.J. Roy markedly decreased NO production-induced LPS with observed IC_50_ values of 5.97 and 6.82 μM, respectively (Chen et al., 2019) (Table 1 and Figure 1).

### Anti-inflammatory activity of meroterpenoids isolated from marine sources

Meroterpenoids isolated from multiple marine sources, such as *Dactylospongia, Kappaphycus alvarezii* (Doty) Doty ex Silva, *Aspergillus*, *Dysidea villosa* (Lendenfeld, 1886), *Dysidea septosa* (Lamarck, 1814), *Corbiculid*, and *Aplidium scabellum* (Michaelsen, 1924), have reported significant anti-inflammatory activity. The report shows that sesquiterpene hydroquinone meroterpenoid dactylospongins A, B, and D, ent-melemeleone B, dysidaminone N, and 19-O-methylpelorol were isolated from the *Dactylospongia* sp. by Jing li et al*.* These compounds exhibited inhibitory activity with IC_50_ values ranging from 5.1 to 9.2 μM on PEG2, IL-6, IL-1β, and IL-8, respectively (Li J. et al., 2018). From *Kappaphycus alvarezii* (Doty) Doty ex Silva, red seaweed ethyl acetate fraction isolated 2-ethyl-6-(4-methoxy-2-((2-oxotetrahydro-2H-pyran-4-yl)methyl)butoxy)-6-oxohexyl-5-ethyloct-4-enoate (C29) reported *in vitro* potential inhibitory activity with IC_50_ 1.04 μg/ml toward 5-lipoxidase pro-inflammatory mediators (Makkar and Chakraborty, 2018). Wang et al. isolated triketide-sesquiterpenoid meroterpene aspertetranones A–D from the *Aspergillus* sp. ZL0-1b14 marine algal-associated fungus. Aspertetranones A and D suppressed the IL-1β and IL-6 production in a dose-dependent manner, whereas aspertetranones B and C, at 33.3 μM concentration, exhibited weak anti-inflammatory effects. Similarly, aspertetranones A–D exhibited weak TNF-α and NO production (less than 35% inhibition) inhibitory effects (Wang Y. et al., 2015). Terpene-polyketide-pyridine hybrid meroterpenoids dysivillosins A–D, isolated from *Dysidea villosa* (Lendenfeld, 1886) by Jiao et al*.*, reported potent inhibitory effect with IC_50_ values of 8.2, 10.2, 19.9, and 16.2 μM in the release of degranulation marker β-hexosaminidase in a dose-dependent manner. The development of LTB4 and IL-4 in antigen-stimulated RBL-2H3 mast cells at 6 and 12 μM, dose-dependently, may be downregulated by all the four meroterpenoids (Jiao et al., 2017). Septosones A and C were isolated from the *Dysidea septosa* (Lamarck, 1814) marine sponge by Gui et al. The study showed that septosone A could inhibit NF-κB activation-induced TNF-α with an IC_50_ value of 6.8 μM in human HEK-293T cells, whereas septosone C with an IC_50_ value of 27.2 μM reported weak inhibitory activity (Gui et al., 2019). Dihydro-5-(8-(9,12-dihydro-8-methyl-11-propyl-2H-pyran-8-yl)-ethyl)furan-2(3H)-one compound reported potential inhibitory activity against pro-enzymes 5-LOX and COX-2 (IC_50_ 0.84 and 0.76 μg/ml), which were obtained from *Corbiculid* bivalve clam (Joy and Chakraborty, 2018). Chan et al. isolated 2-geranyl-6-methoxy-1,4-hydroquinone-4-sulfate, scabellone B, 8-methoxy-2-methyl-2-(4-methyl-3-pentenyl)-2H-1-benzopyran-6-ol, and 2-geranyl-6-methoxy-1,4-hydroquinone meroterpenoids from an extract of *Aplidium scabellum* (Michaelsen, 1924) and reported inhibitory activity with IC_50_ values of 21, 125, 92, and 0.2 *μ*M; *in vitro* human neutrophils stimulated PMA by superoxide production (Chan et al., 2011) (Table 2 and Figure 2).

### Anti-inflammatory activity of meroterpenoids isolated from plants

Meroterpenoids studied from various parts of plants such as *Baeckea frutescens* Linnaeus and *Clinopodium chinense* (Benth.) have been reported as exerting anti-inflammatory activity *via* regulating the signaling NF-κB pathway and also increasing anti-oxidant enzyme activity, Nrf2 levels, and mitochondrial membrane potential.

A study on rare triketone-phloroglucinol-monoterpene baefrutones A–D isolated by Hou Ji Qin et al. from the *Baeckea frutescens* Linnaeus aerial parts with IC_50_ values 9.15–18.04 μM range reported moderate inhibitory activity as comparable to the positive control L-ΜMMA (Hou et al., 2018). Similarly, methanol extract of leaves and twigs isolated meroterpenoids, baeckfrutones (+) N and S, showed potential inhibitory effects with IC_50_ values of 36.21 ± 1.18 and 20.86 ± 0.60 μM on RAW 264.7 macrophages stimulated LPS NO production (Zhi et al., 2018). At concentrations less than 50 μM, baeckfrutone compounds F, G, (+) I, and J reported significant inhibitory activity with rates of 74.64, 75.37, 55.13, and 75.01%, respectively, compared to positive control L-ΜMMA (54.07%) (Qin et al., 2018f). Kuntze et al. from *Clinopodium chinense* (Benth.) aerial parts isolated clinoposides G and H flavonoid-triterpene saponin meroterpenoids significantly reported apoptosis and cell injury inhibition, improved mitochondrial membrane potential, increased anti-oxidant enzymes activity, and reduced the cytokines inflammatory levels. In addition, the compounds also increased the Nrf2 level and decreased the p65 levels in the cell nucleus (Zhu et al., 2018). Hou et al*.* isolated new monoterpene or sesqui-based meroterpenoid frutescones O from the *Baeckea frutescens* Linnaeus aerial parts. This compound showed potent inhibitory activity that could decrease the pro-inflammatory markers TNF-α and IL-6 and influence p65 suppression of nuclear translocation via the NF-κB signaling pathway (Hou et al., 2017) (Table 3 and Figure 3).

### Anti-inflammatory activity of meroterpenoids isolated from algae

Zbakh et al. examined the 11-hydroxy-11-O-methylamentadione (AMT-E) algae meroterpene inhibitory effects in a colitis induced-dextran sodium sulfate (DSS) murine model. The administration of 10 and 20 mg/kg doses of AMT-E significantly decreases 60% and 67% cytokines levels and also decreases IL-10 concentration (Zbakh et al., 2016). Reyes et al. isolated meroterpenoids, usneoidone Z, and 11-hydroxyamentadione-1′-methyl ether from algae *Cystoseira usneoides* (Linnaeus) M. Roberts, 1968, and reported inhibitory activity of TNF-α production by 73% and 64% in LPS-stimulated THP-1 cells (De Los Reyes et al., 2013) (Table 4 and Figure 4).

### COX-2 inhibitory activity of meroterpenoids

#### COX-2 inhibitory activity of meroterpenoids from fungus

Meroterpenoids isolated from fungus *Ganoderma* species have been majorly reported as anti-COX-2 agents to date. Luo et al. isolated meroterpenoid ganotheaecoloid J from *Ganoderma* species and reported its potent COX-2 inhibitory activity (Luo et al., 2018b). From fruiting bodies of *Ganoderma cochlear* (Blume & T. Nees) Bres., (±)-gancochlearols A and B were isolated and reported to have potent COX-2 inhibitory activity (Qin et al., 2018c). Similarly, (±)-spirocochlealactones A–C, new spiro meroterpenoid podimeric enantiomers, and ganodilactone, with IC_50_ values of 1.29–3.63 μM showed potent COX-2 inhibitory activity against lung, immortalized myelogenous leukemia, and hepatic cell lines (Qin F.-Y. et al., 2018). From *Ganoderma* mushrooms, Luo et al. isolated meroterpenoids, ganotheaecolumols A–K, and iso-ganotheaecolumol I, which were tested against COX-2 and JAK3 kinase for their inhibitory activity. It was reported that (±)-ganotheaecolumols C and D, iso-ganotheaecolumol I, and ganotheaecolumols I and K showed inhibitory activity with IC_50_ values of 1.05, 1.38, 2.61, 3.47, and 4.84 μM (Luo et al., 2018a) (Table 1 and Figure 1).

### COX-2 inhibitory activity of meroterpenoids from marine sources

From *Villorita cyprinoides* (Gray et a, 2007), two irregular pyranoids and isochromenyl meroterpenoids dihydro-5-(8-(9,12-dihydro-8-methyl-11-propyl-2H-pyran-8-yl)-ethyl) furan-2(3H)-one and tetrahydro-3-methoxy-5-((E)-8,12-dimethyloct-8-enyl)-pyran-2-one and two hexahydro-isochromenyl-meroterpenoids were identified by Joy et al. The result showed that isolated compounds tetrahydro-3-methoxy-5-((E)-8,12-dimethyloct-8-enyl)-pyran-2-one, (10E)-butyl-9-(6-ethyl-3,4,6,7,8,8a-hexahydro-1H-isochromen-3-yl)-pent-10-enoate, dihydro-5-(8-(9,12-dihydro-8-methyl-11-propyl-2H-pyran-8-yl)-ethyl)furan-2(3H)-one and (12E)-(3,4,6,7,8,8a-hexahydro-1H-isochromen-3-yl)-methyl-hept-12-enoate exhibited COX2 inhibitory activity with IC_50_ > 1.10 (Joy and Chakraborty, 2018) (Table 2 and Figure 2).

### Anti-HIV activity of meroterpenoids

#### Anti-HIV activity of meroterpenoids from the fungus

The anti-HIV activity reported by Liu et al. from the *Periconia* sp. F-31 endophytic fungus isolated new polyketide-terpenoid hybrid molecule periconones B with an IC_50_ value of 18.0 μmol/L compared with positive control efavirenz (Liu J. M. et al., 2017) (Table 1 and Figure 1).

### Anti-HIV activity of meroterpenoids from plants

Tetsuro et al. isolated meroterpenoid daurichromenic acid (DCA) from *Rhododendron dauricum* L. (Ericaceae), which consists of orsellinic acid (OSA) and sesquiterpene moiety. Daurichromenic acid (DCA) was found to be an anti-HIV meroterpenoid produced *via* oxidative cyclization of the farnesyl group of the grifolic acid (Saeki et al., 2018) (Table 3 and Figure 3).

### Alpha-glucosidase inhibitory activity

#### Alpha-glucosidase inhibitory activity of meroterpenoids from the fungus

Meroterpenoids, studied from different fungal species such as *H. caput-medusae* (Bull.) Pers., *Aspergillus terreus* Thom, *Myrothecium* sp. OUCMDZ-2784, and *Ganoderma leucocontextum*, have been reported to show moderate-to-potent α-glucosidase inhibitory activity.

A detailed investigation by Chen et al. led to the isolation of meroterpene dimers containing isoindolinone and caputmedusins A–C from the *H. caput-medusae* (Bull.) Pers. fermentation broth. When evaluated for their α-glucosidase inhibitory function, all isolates displayed moderate inhibition with IC_50_ values of 39.2, 36.2, and 40.8 μM, respectively (Chen L. et al., 2017). In a study by Shan et al., diketopiperazine alkaloidal meroterpenoids, amauromine B and austalide N, were isolated from the *Aspergillus terreus* Thom fungus culture broth. These compounds showed potent inhibitory effects compared with positive control acarbose (Shan et al., 2015). Xu et al. from the *Myrothecium* sp. OUCMDZ-2784 isolated myrothecisins A–D, myrothelactone A, myrothelactone C, tubakialactone B, acremonone G. recombinant expressed in *Saccharomyces cerevisiae* Meyen ex E.C. Hansen*.* All the compounds demonstrated strong inhibitory action against the recombinant human-sourced recombinant α-glucosidase expressed in *Saccharomyces cerevisiae* Meyen ex E.C. Hansen. compared with that of positive control acarbose (Xu et al., 2018). Triterpenes meroterpenoids; ganoleucins A and C; ganomycins I, B, and C; fornicins C and B were isolated by Wang et al. from *Ganoderma leucocontextum* fruiting bodies. These noncompetitively inhibited alpha-glucosidase isolated from yeast and rat small intestine mucosa (Wang et al., 2017) (Table 1 and Figure 1).

### Anti-oxidant activity of meroterpenoids

#### Anti-oxidant activity of meroterpenoids from the fungus

Meroterpenoids from fungal species, such as *Ganoderma sinense*, *Ganoderma capensa* (Lloyd), *Ganoderma cochlear* (Blume & T. Nees) Bres., and *Perenniporia medulla-panis* (Jacq.) Donk (1967) have been studied for anti-oxidant activity using ABTS and DPPH radical scavenging assay. Gao et al. isolated meroterpenoids applanatumol I, from a 95% ethanolic extract of *Ganoderma sinense* fruiting bodies. The outcome revealed that (+)-applanatumol I treatment effectively shielded LO2 cells from cell loss and apoptosis caused by H_2_O_2_. Increased levels of Nrf2, phosphorylation Akt, upregulation of anti-oxidant enzymes, and heme oxygenase 1 (HO-1) were detected in (+)-applanatumols I treated cells; it indicates that the anti-oxidative effects of (+)-applanatumols I by PI3K/Akt-mediated activation of the Nrf2/HO-1 pathway could defend LO2 cells against oxidative harm (Gao et al., 2018). From *Ganoderma capensa* (Lloyd), Peng et al. isolated aromatic meroterpenoids, ganocapensins A and B, ganomycin E, ganomycin F, fornicin E, ganomycin I, fornicin B, and ganomycin C, and reported strong inhibitory activity with IC_50_ values of 6.00 ± 0.11–8.20 ± 0.30 μg/ml compared with positive control Trolox (Peng X. et al., 2016). Additionally, Peng et al. also isolated (±)-cochlearins A–E and G, and three new analogs from *Ganoderma cochlear* (Blume & T. Nees) Bres. cochlearins F, H–I, compared with positive control Trolox. All of the meroterpenoids exhibited inhibitory activity with IC_50_ values in the range of 3.1 ± 0.1–5.3 ± 0.1 μM (Peng X. et al., 2018). From *Perenniporia medulla-panis* (Jacq.) Donk (1967) culture broth, which is a wood-rotting fungus in the Polyporaceae family, Kim et al. isolated xylopyranosyl meroterpenoid. Compound (+) fornicin A with an IC_50_ value of 106.0 μM significant demonstrated DPPH radical scavenging activity, compared with BHA and Trolox as positive controls. On the contrary, perennipins A–C and (+)-fornicin A with IC_50_ values 12.8–190.3 μM range showed anti-oxidant activity against radical scavenging ABTS activity. However, compound (+)fornicin A showed much higher ABTS radical scavenging activity than other compounds (Kim et al., 2019) (Table 1 and Figure 3).

### Anti-oxidant activity of meroterpenoids from marine sources

Meroterpenoids studied from different marine species such as *Hypnea musciformis* (Wulfen), *Kappaphycus alvarezii* (Doty), *Aplidium fuegiense* (Cunningham, 1871), *Corbiculid bivalve* clam, and *Penicillium* sp. YPGA11 has been reported for anti-oxidant activity using radical scavenging ABTS and DPPH assay. Chakraborty et al. studied *Hypnea musciformis* (Wulfen) red seaweed as a potential anti-oxidant. The ethyl acetate fraction of the seaweed yielded three aryls substituted meroterpenoids, namely, 2-(tetrahydro-5-(4-hydroxyphenyl)-4-pentylfuran-3-yl)-ethyl-4-hydroxy benzoate, 2-2-[(4-hydroxybenzoyl)-oxy]-ethyl-4-methoxy-4-2-[(4-methylpentyl) oxy]-3,4-dihydro-2H-6-pyranylbutanoic acid and 3-((5-Butyl-3-methyl-5,6-dihydro-2H-pyran-2-yl)-methyl)-4-methoxy-4-oxobutyl benzoate. Compound 2-(tetrahydro-5-(4-hydroxyphenyl)-4-pentylfuran-3-yl)-ethyl-4-hydroxy benzoate exhibited DPPH radical inhibiting and Fe^2+^ ion chelating activity with IC_50_ 25.05 and 350.7 μM, respectively, followed by 3-((5-butyl-3-methyl-5,6-dihydro-2H-pyran-2-yl)-methyl)-4-methoxy-4-oxobutyl benzoate with IC_50_ 231.2 and 667.9 μM, and 2-2-[(4-hydroxybenzoyl)-oxy]-ethyl-4-methoxy-4-2-[(4-methylpentyl)oxy]-3,4-dihydro-2H-6-pyranylbutanoic acid with IC_50_ 322.4 and 5,115.3 μM (Chakraborty et al., 2016). Makkar et al. isolated and purified meroterpenoid 2-ethyl-6-(4-methoxy-2-((2-oxotetrahydro-2Hpyran-4-yl) methyl) butoxy)-6-oxohexyl-5-ethyloct-4-enoate (C29) from the *Kappaphycus alvarezii* (Doty), (family Solieriaceae) red seaweed methanol: ethyl acetate fraction. The highly oxygenated meroterpenoid C29 showed potential anti-oxidant activity (IC_50_ < 0.35 μg/ml) (Makkar and Chakraborty, 2018). The biologically active derivatives of meroterpene, rossinones A and B, were isolated from the antarctic ascidian *Aplidium* fuegiense array. The inhibitory function of the compounds was tested by Appleton et al. with active human peripheral blood neutrophils. When either N-formyl methionylleucyl phenylalanine (fMLP) (IC_50_ 1.9 and 2.5 μM) or phorbol myristate acetate (PMA) (IC_50_ 0.8 and 0.7 μM) were used to cause the respiratory blast, rossinones A and B were found to inhibit the production of superoxide (Appleton et al., 2009). Joy et al*.* reported two irregular pyranoids and isochromenyl meroterpenoids from the *Corbiculid bivalve* clam, tetrahydro-3-methoxy-5-((E)-8,12-dimethyloct-8-enyl)-pyran-2-one, and dihydro-5-(8-(9,12-dihydro-8-methyl-11-propyl-2H-pyran-8-yl)-ethyl) furan-2(3H)-one while studying bioactivity-guided ethyl acetate: methanol extract of black clam purification. Compound dihydro-5-(8-(9,12-dihydro-8-methyl-11-propyl-2H-pyran-8-yl)-ethyl) furan-2(3H)-one exhibited significantly greater DPPH radical scavenging ability with IC_50_ value < 0.65 μg/ml. Moreover, tetrahydro-3-methoxy-5-((E)-8,12-dimethyloct-8-enyl)-pyran-2-one and dihydro-5-(8-(9,12-dihydro-8-methyl-11-propyl-2H-pyran-8-yl)-ethyl)furan-2(3H)-one was reported for ferrous ion (Fe^2+^) chelating ability with IC_50_ value ∼0.84 μg/ml (Joy and Chakraborty, 2018). Cheng et al. isolated meroterpenoid from the *Penicillium* sp. YPGA11 deep-sea fungus. The isolated compounds were tested in LPS-activated RAW 264.7 macrophages for an inhibitory effect against NO production, whereas quercetin was selected as a positive control. The result showed that compound conidiogenone C exhibited inhibitory effects with an IC_50_ value of 7.58 μM (Cheng et al., 2019) (Table 2 and Figure 2).

### Anti-oxidant activity of meroterpenoids from algae

Meroterpenoids studied from diverse algae species, such as *Cystoseira usneoides* (Linnaeus) M. Roberts, *Cystoseira crinite* Duby, 1830, and *Sargassum siliquastrum* (Mertens ex Turner) C. Agardh, have been reported to show strong radical scavenging activity.

Reyes et al. studied the *Cystoseira usneoides* (Linnaeus) M. Roberts and isolated tetraprenyltoluquinol meroterpenoids, cystodiones A and B, 6-cis-amentadione-1′-Me ether, and amentadione-1′-Me ether. These compounds showed excellent radical scavenging activity (De Los Reyes et al., 2013). Six new derivatives of tetraprenyltoluquinol, two new derivatives of triprenyltoluquinol, and two new derivatives of tetraprenyltoluquinone were isolated along with four known derivatives of tetraprenyltoluquinol from the brown algae *Cystoseira crinita* Duby. All the isolated compounds were tested for anti-oxidant activity. In the DPPH assay, the hydroquinones-based meroterpenoids showed a strong radical scavenging effect in comparison to alpha-tocopherol. These compounds showed inhibitory activity between 13% and 41% in PCL assay (Fisch et al., 2003). *Sargassum serratifolium* (C. Agardh) contains isoprenoid quinones and chromanol meroterpenoids with anti-oxidant activity. DPPH scavenging activity studies revealed that ethyl acetate extract (IC_50_ 34.6 ± 0.47 μg/ml) displayed the strongest activity and ABTS radical scavenging activity followed by methanol extract (IC_50_ 43.2 ± 0.24 μg/ml) (Lim et al., 2019). Kang et al. isolated sargachromanols S and T, two new meroterpenoids, from *Sargassum siliquastrum* (Mertens ex Turner) C. Agardh, with EC_50_ values of 57.1 and 31.1 μM exhibiting mild scavenging activity against the DPPH radical (28.1 μM) and against ABTS radical (15.8 μM) (Kang and Kim, 2017). Similarly, sargachromanols A–P were isolated from the brown alga *Sargassum siliquastrum* (Mertens ex Turner) C. Agardh, sixteen new meroterpenoids of the chromene class in a study by Jang et al. It was reported that chromene class of compounds show anti-oxidant activity; these meroterpenoids were also tested for anti-oxidant activity using DPPH assay. It was found that sargachromanols A–P possessed significant radical scavenging activity with values ranging from concentration 87–91% of 100 µg/ml (Jang et al., 2005) (Table 4 and Figure 4).

### N-acetyltransferase inhibiting activity of meroterpenoids

From the aqueous ethanolic extract of *Ganoderma cochlear* (Blume & T. Nees) Bres. fruiting bodies, Cheng et al. isolated (+)- and (-)-gancochlearol C and ganomycin F, the compounds were tested for N-acetyltransferase inhibition. The findings indicate that (+)-gancochlearol C with an IC_50_ value of 5.29 μM could inhibit N-acetyltransferase (Cheng et al., 2018).

### Anti-microbial activity of meroterpenoids

#### Anti-microbial activity of meroterpenoids from the fungus

Meroterpenoids studied from different fungal species such as *Phyllosticta*, *Penicillium* sp. T2-8, *Cytospora*, and *Aspergillus* have reported moderate-to-potent anti-bacterial activity.

Yang et al. isolated phyllomeroterpenoids A–C and six biosynthetically related compounds (S, Z)-guignardianone C, (S, Z)-botryosphaerin B, (4S, 6R, 9S, 10R, 14R) −17-hydroxylated guignardone A, (S, Z)-phenguignardic acid methyl ester (4S, 6R, 9, 10, 12S, 14R)−12-hydroxylated guignardone A, and (4S, 6R, 9S, 10R, 14R)-guignardone B from fungus *Phyllosticta* sp. Only compound (S, Z)-phenguignardic acid methyl ester with MIC values of 4 μg/ml showed significant anti-microbial activity against *S. aureus* 209P and *C. albicans* FIM709 (Yang et al., 2017). Duan et al. isolated meroterpenoids preaustinoid D and dihydroxyneogrifolic acid, a neogrifolin derivative, Austin, and (S)-18,19-dihydroxyneogrifolin from *Gastrodia elata* Blume, associated with *Penicillium* sp. T2-8 endophytic fungus. The study showed that preaustinoid D and dihydroxyneogrifolic acid with MIC of 128 μg/ml exhibited moderate inhibitory activity against *C. albicans*. Similarly, dihydroxyneogrifolic acid exhibited inhibitory activity against *Bacillus subtilis* (MICs of 8 μg/ml) and *S. Aureus* (MICs of 32 μg/ml), respectively. In addition, Austin and (S)-18,19-dihydroxyneogrifolin with MICs of 4 μg/ml showed activities pointed out against *S. aureus* (Duan et al., 2016). Yun Li isolated from the fungus Cytospora sp. meroterpenoids cytosporolides A–C, three caryophyllene-derived meroterpenoids with a special peroxylactone skeleton. The outcome shows the behavior displayed by all compounds against *S. aureus* and *S. pneumoniae* Gram-positive bacteria, and cytosporolides C was the most potent compound, with IC_50_ values of 1.98 *μ*g/ml and 1.16 *μ*g/ml (Li et al., 2010). Yan He et al*.* isolated spiro meroterpenoids, spiroaspertrione A, and andiconin B from *Aspergillus* sp. endophytic fungus. Both compounds demonstrated inhibition activity against MRSA with MIC values of 4 and 16 μg/ml, respectively (He et al., 2017c). Meroternoidal alkaloid oxalicine C isolated from endophytic fungus *penicillium chrysogenum* has also been reported to have moderate anti-bacterial activity against *Ralstonia solanacearum* (Xu et al., 2020) (Table 1 and Figure 1).

### Anti-microbial activity of meroterpenoids from marine sources

Meroterpenoids studied from diverse species of Okinawan marine sponge and *Aspergillus terreus* Thom (1918) have reported anti-microbial activity for various strains such as *E. coli*, *M. luteus*, *B. subtilis*, *S. aureus*, *C. albicans, A. niger*, and *C. neoformans.*

New meroterpenoid compounds, namely, nakijinol C and nakijiquinone S, have been isolated from marine sponge Okinawan of Spongiidae family by Suzuki et al. Anti-microbial assay of nakijiquinone S and nakijinol C revealed against several bacteria and fungi (*E. coli*, *A. Niger*, *B. subtilis*, *M. luteus*, *T. mentagrophytes*, *S. aureus*, and *C. neoformans*) showed anti-microbial activity (Suzuki et al., 2014). Lei Li et al. identified and isolated aperterpene N and O meroterpenoids, along with terretonins A and G, structurally two known related derivatives, from the marine fungus *Aspergillus terreus* Thom (1918), EN-539. Aperterpene N with an IC_50_ value of 18.0 μM displayed neuraminidase (NA) inhibitory activity. Furthermore, terretonin G demonstrated activity against *M. luteus* (MIC value 32 μg/ml) and *S. Aureus* (8 μg/ml), compared with that of positive control chloramphenicol (Li H. L. et al., 2019). Similarly, Ibrahim et al. isolated (22E, 24R)-stigmasta-5,7,22-trien-3-b-ol and aspernolides F from *Aspergillus terreus* Thom (1918), reporting good activity against *C. neoformans* and *S. aureus.* The compound exhibited a potent action against MRSA, and *C. neoformans* showed 0.96 μg/ml and 4.38 μg/ml IC_50_ values. In addition, aspernolides F showed activity against *C. neoformans* (IC_50_ 5.19 μg/ml) and mild activity against MRSA (IC_50_ 6.39 μg/ml) (Ibrahim et al., 2015). Cheng et al. isolated napyradiomycins A and B3 from *Streptomyces* strains of the MAR4 group. The result showed that these compounds exhibit the most active analogs against MRSA (16 and 2 μg/ml, respectively) (Cheng et al., 2013) (Table 2 and Figure 2).

### Anti-microbial activity of meroterpenoids from plants

Meroterpenoids isolated from various plants, such as *Hypericum yojiroanum* M. Tatewaki & K. Ito*, Melia azedarach* (Linnaeus) and *Callistemon salignus* Craven, were studied for anti-microbial activity on various strains. Reports showed that yojironin A isolated from the entire *Hypericum yojiroanum* M. Tatewaki & K. Ito, vine, action exhibited activity against *A. niger* (IC_50_ 8 *μ*g/ml), *C. albicans* (IC_50_ 2 *μ*g/ml), *C. neoformans* (IC_50_ 4 *μ*g/ml), *Trichophyton mentagrophytes* (IC_50_ 2 *μ*g/ml), *S. aureus* (MIC 8 *μ*g/ml), and *B. subtilis* (MIC 4 *μ*g/ml) (Mamemura et al., 2011). From *Penicillium brasilianum* Bat. found in the root and bark of *Melia azedarach* (Linnaeus), Fill et al. obtained bisphenylpropanoid N-acetylamides, brasiliamide A showed only a weak bacteriostatic effect against *B. subtilis* (MIC of 250 *μ*g/ml) (Fill et al., 2009). Acylphloroglucinol derivatives, callisalignones A–C, and known meroterpenoids, myrtucommulone D and isomyrtucommulone B, were isolated from *Callistemon salignus* in a study by Qin et al*.* The results reported that isomyrtucommulone B exhibited significant activity against *E. coli* (MIC value of 0.122 μg/ml), and myrtucommulone D exhibited potent activity against *S. aureus* and other drug-resistant *S. aureus* strains. Compounds of callisalignone A, 2,6-dihydroxy-4-methoxy-3-methylisopropiophenone, and 2,6-dihydroxy-4-methoxyisovalerophenone displayed moderate activity against *A. fumigatus* (MIC value of 15.625 μg/ml) (Qin et al., 2017a) (Table 3 and Figure 3).

### Anti-microbial activity of meroterpenoids from algae

**FIGURE 5:**
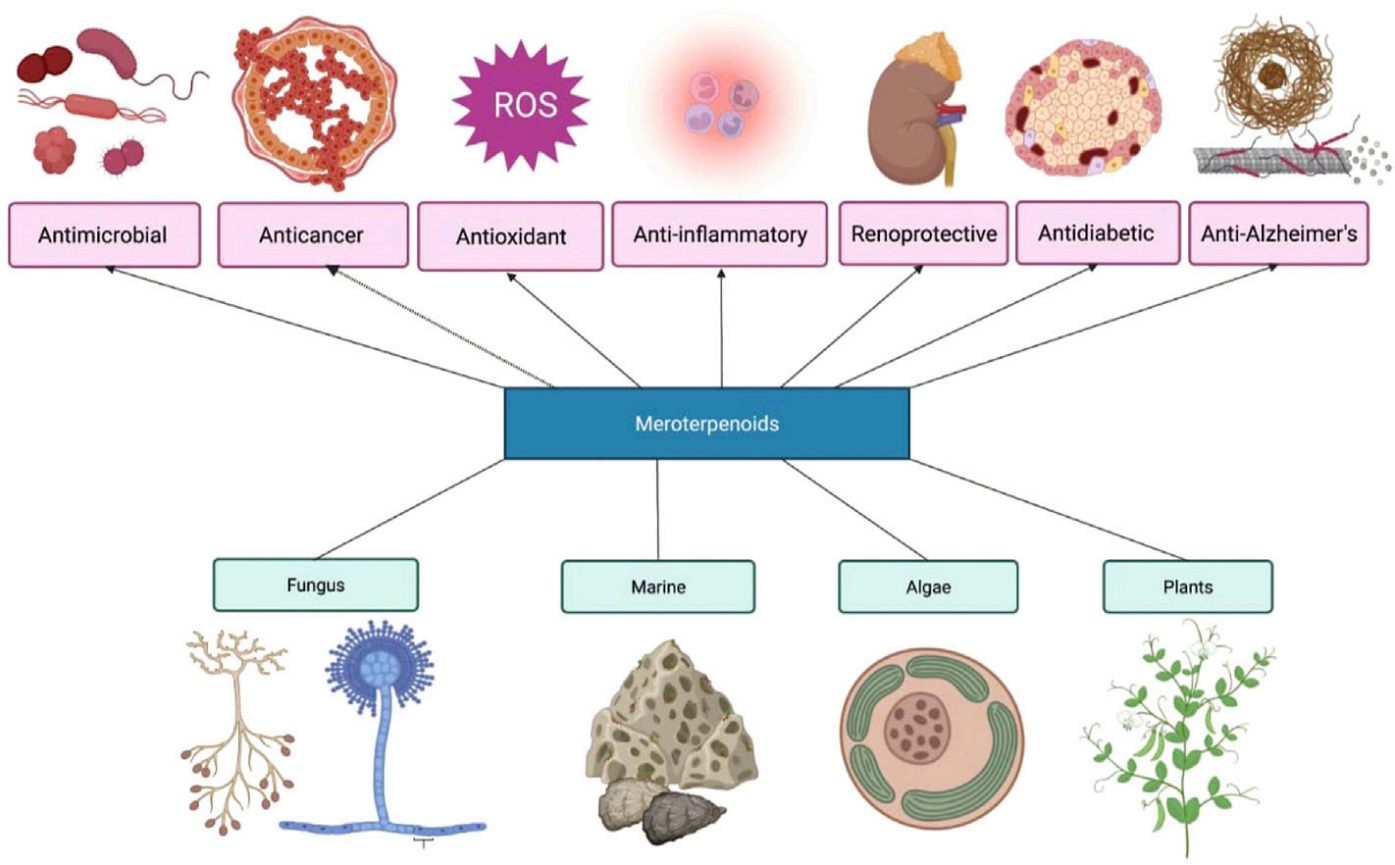
Biological activities of meroterpenoids obtained from various sources.

Phloroglucinol-meroterpenoid cystophloroketals A–D were extracted from alga *Cystoseira tamariscifolia* (Hudson) in a study conducted by Hattab et al. The study showed that cystophloroketals A, B, and D could inhibit the growth of marine bacteria and fungi with MICs values of 1 μg/ml, and cystophloroketals C had the highest inhibitory activity (El Hattab et al., 2015) (Table 4 and Figure 5).

### Anti-bacterial activity of meroterpenoids

#### Anti-bacterial activity of meroterpenoids from the fungus

Meroterpenoids isolated from different fungal species such as *Ganoderma orbiforme* (Fr.) Ryvarden (2000), *Ganoderma cochlear (Blume & T. Nees) Bres.*, *Emericella* species TJ29, *Penicillium*, and *Dysidea* have shown moderate-to-potent anti-bacterial activity against various strains such as *B. cereus*, *S. aureus*, *E. coli*, *P. aeruginosa*, and *S. epidermidis*.

From the cultivated fruiting bodies of *Ganoderma orbiforme* (Fr.) Ryvarden (2000), basidiomycete, norlanostane-type triterpenoids ganoboninone G, and ganomycin I were isolated by Li et al. This research revealed that these compounds exhibited poor action toward *M. tuberculosis* H37Ra (MIC value of 50 μg/ml) and also ganomycin I reported activity against *E. faecium* (MIC 25 μg/ml) Gram-positive bacteria, *B. cereus* (MIC 25 μg/ml), and *S. aureus* (MIC 12.5 μg/ml) (Li W. et al., 2018). In another study, Qin et al. isolated phenolic meroterpenoids (±) cochlearoids O and P from *Ganoderma cochlear* (Blume & T. Nees) Bres*.* These compounds exhibited strong inhibitory activity with IC_50_ values ranging 5.43–17.99 μM against *S. aureus* (Qin F.-Y. et al., 2019). Terpene-polyketide hybrid meroterpenoid, namely, emervaridone A, was isolated from *Emericella* species TJ29. The compounds showed activity against five drug-resistant microbial pathogens [MRSA, *P. aeruginosa*, *Enterococcus faecalis*, *K.* pneumoniae, and β-lactamase-producing *E. coli* (ESBL-producing *E. coli*)]. Emervaridone A also displayed anti-bacterial activity against ESBL-producing *E. coli* and *P. aeruginosa*, in which emervaridone A had MIC values of 2 and 8 μg/ml (He et al., 2017b). Drimane-type sesquiterpene meroterpenoid verruculides B2 isolated from *Penicillium* sp. displayed weak inhibitory with an MIC of 32 μg/ml activity against *S. aureus* (Kong et al., 2017). In another similar study, a fungus *Penicillium citrinum* (Thom, C. 1980), meroterpenoids penicimarins G–H, dehydroaustin, 11β-acetoxyisoaustinone, and austinol exhibited selective anti-bacterial activity. Penicimarin H and austinol showed activity against *S. epidermidis* and *S. aureus* with the same MIC values of 10 µM. Moreover, penicimarins G and H showed a large action spectrum against pathogenic bacteria *S. epidermidis*, *E. coli*, *B. Cereus*, *S. aureus*, *E. coli*, *B. cereus*, and *Vibrio alginolyticus* (Huang et al., 2016). Duan et al. isolated meroterpenoids preaustinoid A1 and (S)-18,19-dihydroxyneogrifolin from *Penicillium* sp. T2-8. The result showed preaustinoid A1 exhibited inhibitory activity against *B. subtilis* (MIC value 4 μg/ml) and (S)-18,19-dihydroxyneogrifolin exhibited potent inhibitory activity against *E. Coli* (MIC value 8 μg/ml) (Duan et al., 2016). Meroterpenoids, dysidphenols A and C, smenospongimine, smenospongine, smenospongorine, smenospongiarine, and smenospongidine isolated from *Dysidea* sp. showed anti-bacterial activity against *E. coli* (25,922), *B. subtilis* (6,633), and *S. aureus* (25,923) strains. Dysidphenols A and C exhibited weak activity against the three strains. However, smenospongimine, smenospongine, smenospongorine, smenospongiarine, and smenospongidine showed potent inhibitory activity in all three strains (Zhang et al., 2016) (Table 1 and Figure 1).

### Anti-bacterial activity of meroterpenoids from marine source

Meroterpenoids studied from different marine species such as *Actinomycete*, *Streptomyces*, and *Spongia* have reported anti-bacterial activity against Gram-positive strains. The report showed that merochlorins E and F, isolated by Ryu et al. from *Streptomyces*, exhibited strong inhibitory activity against *B. subtilis*, *S. aureus*, and *Kocuria rhizophila* (MIC values from 1 to 2 μg/ml) (Ryu et al., 2019). Nguyen investigated Vietnamese marine sponge *Spongia* species and isolated sesquiterpene hydroxyquinone langcoquinone C and smenospongorine, which had significant activity against *S. aureus* and *B. subtilis* with MIC ranging from 6.25 to 25 µM (Nguyen et al., 2017). Sesquiterpene aminoquinones langcoquinones A–B, dictyoceratin A, ilimaquinone, smenospongine, smenospongidine, and nakijiquinone L from the marine sponge *Spongia* species exhibited significant inhibitory activity against *S. aureus* and *B. subtilis* with MICs in a range of 6.25–12.5 µM (Li H. et al., 2018). Haste et al. isolated two naphthoquinone meroterpenoids (A80915A and A80915B) produced by actinomycete, marine-derived, *Streptomyces* sp. CNQ-525 strain. These compounds demonstrated strong and fast bactericidal action against modern strains of MRSA (Haste et al., 2011) (Table 2 and Figure 2)

### Anti-bacterial activity of meroterpenoids from plants

Three phloroglucinols meroterpenoids, aspidin BB, desaspidin BB, and desaspidin PB, isolated from *Dryopteris championii* (Benth.), were tested against the *S. aureus*, *E. coli*, *B*. *subtilis*, and *Dickeya zeae* (MIC values between 4 and 16 μg/ml) (Chen et al., 2016). Two meroterpenoids, eugenials C and D, isolated from the *Eugenia Umbelliflora* (O.Berg) fruits, reported strong activity against *B. Subtilis*, *S. aureus*, and MRSA (Li H. et al., 2018) (Table 3 and Figure 3).

### Anti-bacterial activity of meroterpenoids from algae

Meroterpenoid sargachromanol L of the chromene class was isolated from *Sargassum siliquastrum* (Mertens ex Turner) C. Agardh brown algae. The result showed that sargachromanols L exhibited weak anti-bacterial activity (Chung et al., 2011) (Table 4 and Figure 4).

### Antitubercular activity of meroterpenoids

Quinone and hydroquinone-based meroterpenoids, deacetoxyyanuthone A, macrophorin A, and 4′-oxomacrophorin, were isolated by Jun He et al. from fungus *Gliomastix* sp. ZSDS1-F7. The result showed that these compounds showed important inhibitory action against *M. tuberculi* with IC_50_ values of 22.1, 2.44, and 17.5 µM, respectively (He W. J. et al., 2017).

### Anti-fungal activity of meroterpenoids

#### Anti-fungal activity of meroterpenoids from fungus

Zhang et al. synthesized and explored the anti-fungal activity of meroterpenoid (+)-chromazonarol and (+)-yahazunone. The findings revealed that these compounds showed beneficial activity with EC_50_ values of 24.1 and 28.7 μM against *Sclerotinia scleotiorum* (Zhang S. et al., 2018). Endophytic fungus *Phyllosticta* sp. WGHL2 also showed four new meroterpenoids, namely, guignardones U–X, along with known meroterpenoids. However, none of the four newly isolated compounds showed anti-fungal activity (Yan et al., 2021) (Table1 and Figure 1).

### Anti-fungal activity of meroterpenoids from marine sources

Cohen et al. isolated meroterpenoid insuetolides A, strobilactone A, and (E, E)-6-(60,70-dihydroxy-20,40-octadienoyl)-strobilactone A from ethyl acetate extract of the culture medium of the marine-derived fungus *Aspergillus insuetus* (Bainier) Thom & Church (1929)*.* The MIC values of these compounds against the fungus *Neurospora crassa* were 140, 242, and 162 μM, respectively (Cohen et al., 2011). Merosesquiterpene 24-methylsulfinyllancoquinone B isolated from marine sponge *Spongia pertusa* has been reported for its moderate anti-fungal activity against human pathogens, namely, *Candida albicans* and *Trichophyton* species (Tang et al., 2022) (Table 2 and Figure 2).

### Anti-fungal activity of meroterpenoids from plants

Meroterpenoids studied from various species of plants, such as *Eucalyptus robusta* Smith and *Psoralea glandulosa* L., have been reported to date to possess anti-fungal activity in their different parts.

From the leaves of *Eucalyptus robusta* Smith, formyl phloroglucinol (FPM) meroterpenoids, namely, eucalrobusones T, U, and X, were isolated by Shang et al*.* The results showed that eucalrobusones T and U exhibited significant activity MIC_50_ values less than 10 μg/ml against *C. glabrata*. Eucalrobusone X showed the strongest activity with an MIC_50_ value of 10.78 μg/ml against *C. albicans*. It was also found that FPMs are more effective against *C. glabrata* than *C. albicans* (Shang et al., 2019). A similar study was conducted on FPMs, namely, eucalrobusones J and O, isolated from the leaves of *Eucalyptus robusta* Smith by Shang et al*.* The result showed that compounds eucalrobusones J and O exhibited significant inhibitory activity against *C. glabrata* and eucalrobusone O also showed moderate activity against *C. albicans* (Shang et al., 2016b). Similarly, from extracts of *Psoralea glandulosa* L., Madrid et al. isolated meroterpenoids, namely, bakuchiol and 3-hydroxybakuchiol. Both compounds demonstrated potent activity with the MIC_80_ ranging from 4 to 416 and 0.125–16 μg/ml, respectively, against the strains of *C. albicans* ATCC7978 and *Candida parapsilosis* ATCC22019 (Madrid et al., 2012) (Table 4 and Figure 4).

### Beta-site amyloid precursor protein cleaving enzyme 1 (BACE1) inhibitory activity of meroterpenoids

#### BACE1 inhibitory activity of meroterpenoids

Meroterpenoids studied from two fungal species, namely, *Aspergillus terreus* Thom (1918) and *S. serratifolium* (C. Agardh), have been reported to show moderate-to-potent BACE1 inhibitory activity.

Qi et al. investigated various DMOA meroterpenoids from the fungus *Aspergillus terreus* Thom (1918) for BACE1 inhibitory activity. Terreusterpenes A and B inhibited BACE 1 with IC_50_ values of 5.98 and 11.42 μM. Terreusterpene D exhibited promising inhibitory activity (IC_50_ values of 1.91 μM); asperterpenes E, F, and J exhibited significant inhibitory activity (IC_50_ values of 3.3, 5.9, and 31.7 μM); and asperterpenes A and B demonstrated moderate activity (IC_50_ values of 78 and 59 μM) (Qi et al., 2016; 2018b; 2018a). Seong et al. isolated sargahydroquinoic acid, sargaquinoic acid, and sargachromenol meroterpenoids from *S. serratifolium* (C. Agardh) and tested them for anti-Alzheimer’s disease (AD) activity. The study demonstrated that all three compounds exhibited potent inhibitory activity compared with quercetin (Seong et al., 2017). A study on spiroterreusnoids A–F spiro-dioxolane meroterpenoids isolated by Changxing et al. from *A. terreus* with IC_50_ values 5.86–27.16 μM range showed potential BACE1 inhibitory effects (Qi et al., 2019). Yatsu et al. isolated 4-hydroxybenzoic acid-based meroterpenoids from fruiting bodies of *B. asiaticus*. Asiaticusinol C, asiachromenic acid, and asiaticusin A showed BACE1 inhibitory activity with IC_50_ values between 2 and 14 μM (Yatsu et al., 2019) (Table 1 and Figure 1).

### Renal protective effect of meroterpenoids

Luo et al. isolated applanatumols A and (+)-B from *Ganoderma applanatum* (Pers.) Pat. 1887. The biological activity of these compounds toward renal fibrosis was evaluated in rat proximal tubular epithelial cells. The results show that applanatumols A and (+)-B could inhibit extracellular matrix (ECM) components (fibronectin and collagen I) (Luo et al., 2016).

### Acetylcholinesterase inhibitory activity of meroterpenoids

#### Acetylcholinesterase inhibitory activity of meroterpenoids from the fungus

Various species of *Ganoderma*, *Aspergillus*, and *Penicillium* fungus have yielded meroterpenoids that have shown potent AchE inhibiting activity.

Qi et al*.* investigated DMOA-based meroterpenoid, terreusterpene D, obtained from *A. terreus*. The compounds with an IC_50_ value of 8.86 μM exhibited promising AchE inhibitory activity, which could also serve for Alzheimer’s disease treatment (Qi et al., 2018b). From *Aspergillus* 16-5c, Long et al. isolated polyketide-terpenoid meroterpenoids, namely, isoaustinol, dehydroaustin, and dehydroaustinol, and reported potent AchE inhibiting activity (Long et al., 2017). Polycyclic-meroterpenoid enantiomers ganocin D isolated by Peng et al. from the *Ganoderma cochlear* (Blume & T. Nees) Bres. fruiting bodies showed weak inhibition with an inhibition of 32% (50 μM) (Peng et al., 2014). Luo et al. isolated (+)-zizhines G, (−)-zizhines G, (−)-ganosinensols A, (+) zizhines P, (−) zizhines P, (+)-zizhines Q, and (−) zizhines Q from *Ganoderma* species. All the compounds exhibited inhibitory activity with inhibition rates of 88.77%, 87.68%, 82.18%, 89.24%, 87.73%, 83.43%, and 83.71%, respectively, at the concentration of 50 μM using tacrine as a positive control (Luo et al., 2019a). Aromatic meroterpenoid ganocapenoid C, ganocalidin E, cochlearin I, and patchiene A were isolated from *Ganoderma capense* (Lloyd). These compounds showed inhibition with the IC_50_ values of 28.6 ± 1.9, 8.7 ± 1.6, 8.2 ± 0.2, and 26.0 ± 2.9 μM, respectively (Liao et al., 2019). Dai et al. isolated meroterpenoid arisugacins D, M, O, P, and Q from *Penicillium* species in a phenotype-based zebrafish assay. The compound arisugacin D has been reported as a selective inhibitor with an IC_50_ value of 3.5 μM. Compounds arisugacin M, O, P, and Q induced paralysis in zebrafish embryos, with arisugacin O demonstrating potent and selective inhibitory activity (Dai et al., 2019). A study on spiroterreusnoids A–F spiro-dioxolane meroterpenoids extracted by Changxing et al. from fungus *Aspergillus terreus* Thom (1918) showed moderate AchE inhibitory effects, with IC_50_ values ranging from 22.18 to 32.51 *μ*M (Qi et al., 2019) (Table 1 and Figure 1).

### Acetylcholinesterase inhibitory activity of meroterpenoids from marine sources

Huaqiang Li et al. obtained asperversins G from the fungus *Aspergillus versicolor* (Vuill), which exhibited an inhibitory effect (IC_50_ of 13.6 μM) (Li H. et al., 2018). Ding et al. isolated α-pyrone meroterpenoids 3-epiarigsugacin E, territrem C, arisugacin B, and terreulactone C from the fungus *Penicillium* sp. SK5GW1L. The result showed that compound 3-epiarigsugacin E exhibited weak inhibitory activity compared to arisugacin B, territrem C, and terreulactone C (IC_50_ values of 3.03, 0.23, and 0.028 μM) (Ding B. et al., 2016) (Table 2 and Figure 2).

### Acetylcholinesterase inhibitory activity of meroterpenoids from plants

Qin et al. isolated dimeric phellandrene-derived meroterpenoids *Eucalyptus* dimer A, (±) eucalyprobusone A, from fruits of *Eucalyptus robusta* Smith, and triketone sesquiterpene type meroterpenoid rhodomyrtusials A, rhodomyrtusials B, and tomentodione Q from *Rhodomyrtus tomentosa. Eucalyptus* dimer A, (±) eucalyprobusone A, rhodomyrtusials A, rhodomyrtusials B, and tomentodione Q with IC_50_ values of 17.71, 13.61, 8.8, 6.0, and 6.6 μM exhibited inhibitory activity, respectively (Qin X.-J. et al., 2018; Qin et al., 2019 X.). Luo et al. isolated meroterpenoids dayaolingzhiols D and E from *Ganoderma lucidum* (Karst). These reported strong inhibitory activity with IC_50_ values of 8.52 and 7.37 μM, respectively (Luo et al., 2019b) (Table 3 and Figure 3).

### Acetylcholinesterase inhibitory activity of meroterpenoids from algae sources

Seong et al. isolated sargahydroquinoic acid, sargachromanol, and sargaquinoic acid meroterpenoids for anti-Alzheimer’s disease (AD) activity from *S. serratifolium* (C. Agardh). The result showed that all three compounds exhibited moderate inhibitory activity compared with berberine (Seong et al., 2017) (Table 4 and Figure 4).

### Protein tyrosine phosphatase (PTP1B) inhibitory activity of meroterpenoids

#### PTP1B activity of meroterpenoids from marine

Preaustinoid-related meroterpenoids, preaustinoid A6, and berkeleyone C were isolated and identified from *Penicillium* species on the chemical investigation by Park et al. The compounds inhibited PTP1B activity with IC_50_ values of 17.6 and 58.4 μM. It was also found that compound preaustinoid A6 lowered the apparent value of V_max_ and increased the K_i_ value of 17 μM, indicating that it inhibited PTP1B in a non-competitive manner (Park et al., 2019) (Table 2 and Figure 2).

### PTP1B activity of meroterpenoids from plants

Meroterpenoids from species *Magnolia* and *Rhododendron* have been extensively studied for PTP1B inhibiting activity. Li et al. isolated polycyclic meroterpenoid magterpenoids A and C from ethanolic extract bark of *Magnolia officinalis* (Rehder & Wilson) var. biloba. The result displayed PTP1B with IC_50_ values of 1.44 and 0.81 μM, respectively (Li C. et al., 2018). Meroterpenoids enantiomeric pairs, (−) and (+)-rhodonoid B, were extracted from partly racemic mixtures that existed naturally in *Rhododendron capitatum* (Maxim.). The result demonstrated inhibition (IC_50_ values of 43.56 and 30.38 μM) compared to positive control oleanolic acid (Liao et al., 2015). From *Rhododendron nyingchiense* (R.C. Fang & S.H. Huang), Huang et al. isolated meroterpenoids, (+) nyingchinoids A and B, (−) nyingchinoids C and D, (±)-nyingchinoids H, and grifolin. The study showed that the compounds with IC_50_ values between 5.7 ± 0.5 and 61.0 ± 4.8 μM exhibited weak inhibitory effects (Huang et al., 2018). Li et al. isolated compounds of magmenthanes E and H from *Magnolia officinalis* (Rehder & Wilson) var. Biloba bark. The compounds displayed significant inhibition against PTP1B (IC_50_ values of 4.38 and 3.88 μM) (Li C. et al., 2019) (Table 3 and Figure 3).

### Bromodomain-containing protein 4 (BRD4) inhibitory activity of meroterpenoids

Bromodomain-containing protein 4 is a transcriptional and epigenetic protein in humans encoded by the *BRD4* gene. *BRD4* plays a critical role in cancer growth and embryogenesis and is responsible for the development of many diseases. BRD4 inhibited by molecules can be developed as anti-viral, anti-inflammatory, anti-proliferative, and anticancer drugs (Qin F.-Y. et al., 2019).

The fruiting bodies of *Ganoderma cochlear* (Blume & T. Nees) Bres*.* have isolated (±) cochlearoids N–P, three pairs of meroterpenoids. The outcome revealed that (±) cochlearoid N showed a *BRD4* inhibitory effect against K562 cells with IC_50_ values of 7.68 and 6.68 μM (Qin F.-Y. et al., 2019) (Table 1 and Figure 1).

### Anti-Kaposi’s sarcoma-associated herpes virus activities of meroterpenoids

Kaposi’s sarcoma-associated herpes virus (KSHV) is a double-stranded DNA-based carcinogenic pathogen. KSHV is involved in Kaposi’s sarcoma diseases, AIDS, Castleman’s disease, and primary lymphoma drugs such as ganciclovir, cidofovir, or nelfinavir, and the target is generally used to inhibit KSHV replication. However, this drug cannot restrain the virus effectively. Therefore, natural products such as meroterpenoids were investigated as KSHV inhibitors (Hu et al., 2018).

Hu et al. investigated acylphloroglucinol-based meroterpenoid japonicols E and H from *H. japonicum* (Thunb.). The result exhibited strong inhibition toward the lytic replication in Vero cells (IC_50_ values of 8.30 and 4.90 μM) (Hu et al., 2018).

### Immunosuppressive activity of meroterpenoids

By effective genome mining, arthripenoid C was isolated from two fungi targeting genetically proximal genes from polyketide and terpenoid biosynthesis. These compounds inhibit concanavalin- (ConA-) induced T-cell proliferation (IC_50_ values of 8.8 μM). In addition, both TNF-α and IFN-γ were substantially secreted from activated T cells in response to stimulation with ConA, which was markedly attenuated with IC_50_ 4.2 and 12.1 μM treatment with arthripenoid C (Zhang X. et al., 2018).

### Effect of meroterpenoids in obesity and non-alcoholic fatty liver disease

Kwon et al. investigated the effect of meroterpenoids from ethyl acetate fraction of *Sargassum serratifolium* (C. Agardh) (ESS) on obesity and related stenosis on the administration of a high-fat diet to C57BL/6J mice. EES supplementation restored the phosphorylation levels of AMP-activated protein kinase (AMPK) and reduced lipogenic proteins. Thus, ESS exerted the anti-obesity and lipid-lowering effects by activating AMPK-related fatty acid oxidation signaling in the adipocyte’s cells. The study concluded that EES has the ability to prevent diet-induced obesity and related metabolic disorders by inhibiting lipogenesis and adipogenesis in 3T3-L1 preadipocytes and activating energy expenditure (Kwon et al., 2018a; 2018b).

### Effect of meroterpenoids in sodium channel activation, inactivation, and window currents

Electrophysiological influences on the gating kinetics of voltage-gated sodium channels in central neurons were tested for acetoxydehydroaustin A and austin, isolated from *Verticillium albo-atrum* (Reinke & Berthold, 1879) fungus. They also improved the recovery time from rapid sodium channel inactivation. These findings found that both compounds affected the activation, inactivation, and window currents of the sodium channel (Wu et al., 2018).

### Anti-viral activity of meroterpenoids

#### Anti-viral activity of meroterpenoids from the fungus

The anti-viral activity of *Penicillium* and *Aspergillus* isolated meroterpenoids has been reported. Austalide U, merochlorin D, austalide I, and austalide P acid meroterpenoids were isolated from *Aspergillus aureolatus* (Muntañola-Cvetkovic & Bata, 1964) HDN14-107 sponge-derived fungus culture by Peng et al*.* The CPE inhibition assay assessed the anti-influenza A virus (H1N1) activities of these compounds. The results showed that compounds with IC_50_ values of 90, 99, 131, and 145 μM exhibited inhibitory effects (Peng J. et al., 2016). Drimane-type sesquiterpene meroterpenoids chrodrimanins K and N and 3-hydroxypentacecilide A isolated from *Penicillium* sp. SCS-KFD09 displayed anti-H1N1 activity (IC_50_ values of 74, 58, and 34 μM) (Kong et al., 2017). Chrodrimanins A, E, and F isolated from *Penicillium funiculosum* (Thom, 1910) GWT2-24 showed inhibition against influenza A virus (H1N1) (IC_50_ values of 21, 55, and 57 μM) compared to that of the positive control ribavirin (Zhou et al., 2015) (Table 1 and Figure 1).

### Anti-viral activity of meroterpenoids from marine sources

Polycyclic meroterpenoid talaromyolide D, obtained from the marine fungus *Talaromyces* sp. CX11, exhibited an inhibitory activity with a CC_50_ value of 3.35 μM against the pseudorabies virus (PRV) (Cao et al., 2019) (Table 2 and Figure 2).

### Anti-viral activity of meroterpenoids from plants

Liao et al. performed a chemical investigation on the *Rhododendron capitatum* (Maxim.) aerial parts and isolated enantiomeric meroterpenoid and (+)-rhodonoid C. The anti-viral activity was evaluated against the HSV-1 *in vitro* study using the cytopathic effect (CPE) assay with acyclovir as the positive control. The compound showed inhibitory activity against HSV(IC_50_ value of 80.6 ± 4.7 µM) (Liao et al., 2017). The hybrid polyketide-terpenoid stachybonoid A isolated from fungus *Stachybotrys chartarum* (Ehrenb.) 952 reported inhibitory activity against the dengue virus replication (Liu Z. et al., 2018). Linzhen hu et al. isolated filicinic acid-based meroterpenoid hyperjaponols B and D from *Hypericum japonicum* (Thunb.). The compounds were assessed for activity against the anti-Epstein–Barr virus. The compounds with EC_50_ values of 0.57 and 0.49 μM showed an inhibitory effect on the Epstein–Barr virus (Hu et al., 2016) (Table 3 and Figure 3).

### Neuroinhibitory activity of meroterpenoids

Matos et al. investigated hydroquinones and benzoquinone-based meroterpenoid compounds from *Cordia oncocalyx* (F. Allum.). They isolated a new compound rel-1,4,8α-trihydroxy-5-furanyl-2-methoxy-8aβ-methyl-6,7,8,8a,9,10-hexahydro-10-anthracenone, reported to possess the neuroinhibitory activity, and none of the pharmacological antagonists was reversed. Additionally, compounds rel-1,4,8α-trihydroxy-5-furanyl-2-methoxy- 8aβ-methyl-6,7,8, 8a, 9,10-hexahydro-10-anthracenone and 6-formyl-2-methoxy-9-methyl-1,4-phenanthrendione were able to inhibit the 69% and 63% contractions, respectively (Matos et al., 2017).

### Neuroprotective activity of meroterpenoids

From *Ganoderma austral*, meroterpenoids ganomycin C, (−)-ganoresinain A, ganotheaecoloid G were isolated by Zhang et al. The compounds were tested in glutamate-induced SH-SHY cells for neuroprotective activity. The result showed that these compounds prevent glutamate-mediated cellular toxicity of neural cells (Zhang J. J. et al., 2019). Benzylic phloroglucinol-terpene hybrid type meroterpenoid, namely, melaleucadines A and B, were isolated by Kie et al. from branches and leaves of *Melaleuca Leucadendron* (L.) L. These compounds possessed neuroprotective activity on Cort-induced PCI-2 cell injuries with cell viability of 53.72% and 58.38%, respectively, at 50 µM (Xie et al., 2019).

### JAK3 inhibitory activity of meroterpenoids

Spiroapplanatumines G and H spiro meroterpenoids were isolated from *Ganoderma applanatum* (Pers.) Pat. 1887, fungus. The results showed that these compounds with IC_50_ values of 7.0 ± 3.2 and 34.8 ± 21.1 μM display inhibitory properties on JAK3 kinase (Luo et al., 2017).

### Anti-plasmoid activity of meroterpenoids

Cadelis et al. studied thiaplidiaquinones A and B and their effect against the NF54 strain of chloroquinone-sensitive *P. falciparum*. The prenyl and farnesyl analogs exhibited moderate activity against *P. falciparum* (Welch, 1897) (IC_50_ 0.29 mM), with the farnesyl series exhibiting greater selectivity (Cadelis et al., 2017).

Chan et al. conducted a bioassay of the New Zealand ascidian *Aplidium scabellum* (Michaelsen, 1924) that yielded pseudodimeric meroterpenoid, namely, scabellone B. The compound exhibited selectivity toward *Plasmodium falciparum* (Welch, 1897) (IC_50_ 4.8 *μ*M) (Chan et al., 2011).

### HMG-CoA reductase inhibitory activity of meroterpenoids

Triterpene meroterpenoids ganomycins I, B, and C were isolated by Wang et al. from fruiting bodies of *Ganoderma leucocontextum* (T. H. Li, W. Q. Deng, Dong M. Wang & H. P. Hu, 2015). These compounds exhibited stronger inhibition compared to the positive control atorvastatin against HMG-CoA reductase (Wang et al., 2017).

### Renal protective activity of meroterpenoids

Petchiethers A and B, isolated from *Ganoderma petchii* (Lloyd) Steyaert, 1972, were tested for the inhibition of overproduction of fibronectin. The results show that both compounds could inhibit the development of fibronectin in a dose-dependent manner and achieve maximal effects at 20 μM concentrations (Li C. G. et al., 2016). Phenolic meroterpenoids, namely, cochlearoids (F–I, K), cochlearol (K, S, U, X, and Y), and cochlearin E, isolated from *Ganoderma cochlear* (Blume & T. Nees) Bres. demonstrated an inhibitory effect against TGF-β1-induced HKC-8 cells and TGF-β1-induced NRK-49F cells, respectively. Cochlearoids (F–I, K) showed a potential inhibitory effect on fibronectin overproduction in TGF-β1-induced HKC-8 cells. Similarly, cochlearols (K, S, U, X, and Y) and cochlearin E inhibited fibronectin overproduction in TGF-β1-induced NRK-49F cells (Wang X. L. et al., 2016; 2019b; 2019a). Racemic polycyclic meroterpenoid (+)- and (−)-cochlearols A and B isolated from *Ganoderma cochlear* (Blume & T. Nees) Bres. reported inhibitory activity of collagen I, fibronectin, and α-SMA in a dose-dependent manner in TGF-β1- induced rat renal proximal tubular cells. Also, (−)-cochlearol B showed strong inhibitory activity against p-Smads in TGF-β1- induced rat renal proximal tubular cells (Dou et al., 2014). Luo et al. isolated chizhine F, fornicin B, and ganomycin I from *Ganoderma lucidum* (Curtis) P. Karst., which inhibited the MCP-1 expression in high-glucose-induced mesangial cells in a dose-dependent manner (Luo et al., 2015). Lactone fused meroterpenoid lingzhilactone B isolated from *Ganoderma lingzhi* (Sheng H. Wu, Y. Cao & Y.C. Dai, 2012) reported an inhibitory effect in adriamycin-induced nephropathy mice. The *in vitro* and *in vivo* results suggested that lingzhilactone B inhibits various activities such as ROS generation, increased expression of Nrf2, mRNA expression of collagen IV, and fibronectin in rat tubular epithelial cells. It also could reduce urinary albumin levels, inhibit the phosphorylation of Smad3, and protect against renal injuries by inhibiting inflammation and increasing the activity of anti-oxidants (Yan et al., 2015b).

### Anti-fibrotic activity of meroterpenoids

Ding et al. isolated lingzhifuran A and lingzhilactone D, phenolic meroterpenoids, from the fruiting bodies of *Ganoderma lucidum* (Curtis.) P Karst. The compounds exhibited Smad3 phosphorylation inhibition (Ding W. Y. et al., 2016).

### Cardioprotective activity of meroterpenoids

Zhu et al. isolated flavonoid-triterpene saponin meroterpenoids, namely, clinoposides B, D, and F, which showed cell viability of 87.2 ± 7.7%, 82.7 ± 8.3%, and 90.8 ± 6.5% at 25.0 μg/ml using quercetin and ginsenoside Rb 1 as a positive control. All three compounds showed better protective effects as evidenced by increased levels of SOD, CAT, and GSH-Px and reduced MDA, LDH, caspase-3, and caspase-9 levels (Zhu et al., 2016).

### Anti-leishmanial activity of meroterpenoids

Two stigmasterol derivatives, (22E, 24R)-stigmasta-5,7,22-trien-3-β-ol, stigmast-4-en-3-one, isolated from the roots of *Carthamus lanatus* L. (Asteraceae) showed good exhibition toward *L. donovani* (IC_50_ values of 4.61 and 6.31 μg/ml) (Ibrahim et al., 2015) (3R)- and (3S)-tetraprenyltoluquinol and (3R)-tetraprenyltoluquinone and (3S)-tetraprenyltoluquinone, isolated from *Cystoseira baccata* (S. G. Gmelin) P. C. Silva, 1952, could inhibit the growth of the *L. infantum* (Nicolle, 1908) promastigotes (IC_50_ 44.9 and 94.4 μM). Compound (3R)- and (3S)-tetraprenyltoluquinol decreased the intracellular infection index (IC_50_ = 25.0 ± 4.1 μM). Disulfated meroterpenoids, isoakaterpin, from extracts of *Callyspongia* sp. exhibited inhibition of *Leishmania* spp. adenosine phosphoribosyl transferase (IC_50_ of 1.05 µM) (Gray et al., 2007) (Table 3 and Figure 3).

### Gastroprotective activity of meroterpenoids

Meroterpenoids sargaol, epitaondiol, stypodiol, and isoepitaondiol were isolated from the *Stypopodium flabelliforme* Weber-van Bosse, 1913, Chilean Seawood by Areche et al*.* The gastroprotective activity was evaluated using a gastric ulcer ethanol/HCL-induced mice model. Among meroterpenoids obtained, sargaol and epitaondiol with ED_50_ values of 35 and 40 mg/kg reported gastroprotective activity, respectively. Oral administration of stypodiol and isoepitaondiol at 40 mg/kg blocked 69% and 78% of the appearance of gastric mucosal lesions in mice, respectively (Areche et al., 2015). (Table 2 and Figure 2).

### Neural stem cell proliferation activity of meroterpenoids

Yan et al. isolated spirolingzhines A–D, lingzhines (B, D–F), and 4-(2,5-dihydroxyphenyl)-4-oxobutanoic acid meroterpenoids from the fruiting bodies of the *Ganoderma lingzhi* (Sheng H. Wu, Y. Cao & Y.C. Dai), 2012, fungus. In order to determine whether the isolated compounds affect the CNS, their ability to regulate adult NSCs from P7 mouse dentate gyrus was evaluated. The results showed that these compounds promoted NSC proliferation (-)-spirolingzhine A, which was found to exhibit the highest NSC proliferation activity comparable to the positive control forskolin (Yan et al., 2015a).

### Inhibition of AP-1 activity of meroterpenoids

In a study by Zhuravlena et al., isolated meroterpenoids, austalide H acid butyl ester, 13-O-deacetylaustalide I, austalide H acid, and 13-deacetoxyaustalide I, were isolated from *Penicillium lividum* Thom, C. KMM 4663 and *Penicillium thomii* Maire, R.C.J.E. 1917, KMM 4645. The outcome reported that the transcriptional activity of AP-1 oncogenic nuclear factor of JB6 Cl41 cells was inhibited at noncytotoxic concentrations after 12 h of treatment by these compounds. At 6.25 μM concentration, these compounds exhibited inhibitory activity, whereas the reduction of cell viability up to 100 μM was not observed (Zhuravleva et al., 2014).

### Insecticidal activity of meroterpenoids

Meroterpenoid dhilirolide L isolated from the fungus *Penicillium purpurogenum* Stoll (1923) by Centko et al. showed inhibitory activity and exhibited sublethal developmental disruption at low concentrations in the *Trichoplusia ni* (Hübner, 1800–1803) cabbage looper (Centko et al., 2014). Chrodrimanin-type (A, B, E, H, G, and F) meroterpenoids from the solid cultures of a mangrove endophytic fungus *Diaporthe* sp. SCSIO 41011 showed inhibitory insecticidal activity of GABA-gated chloride channels as potent and selective blockers of insects (Luo X. W. et al., 2019). Chondrimanins D–F were isolated by Hayashi et al. from okara, which is the solid residue of soybean, fermented with the YO-2 strain of *Talaromyces* sp., showing inhibitory activity against silkworms with LD_50_ values of 20, 10, and 50 μg/g of diet (Hayashi et al., 2012). Bai et al. isolated meroterpenoids, namely, penicianstinoids A and B, furanoaustinol, austinol, 1,2-dihydro-7-hydroxydehydroaustin, 7-hydroxydehydroaustin, and dehydroaustinol from bioactive metabolites of *Penicillium* sp. The researchers reported inhibitory with EC_50_ values of 9.4, 9.9, 19.1, 19.5, 20.5, 20.6, and 38.2 μg/ml against *C. elegans* (Bai et al., 2019).

### Selective inhibitors of the p-Smad3 activity of meroterpenoids

(+)-Lingzhiol and (-)-lingzhiol, a pair of rotary door-shaped meroterpenoid enantiomers, were isolated from *Ganoderma lucidum* Karst (1881) by Yan et al. to study the effect against diabetic nephropathy (+)lingzhiol and (-)-lingzhiol, demonstrating inhibition of TGF-β1-induced p-Smad3 in renal proximal tubular cells of rat and initiating the production of Nrf2/Keap1 in mesangial cells (Yan et al., 2013).

### Inhibitors of Na+/K + ATPase activity of meroterpenoids

Sargachromanols D, F, H, and L are the meroterpenoids of the chromene class isolated from the *Sargassum siliquastrum* (Mertens ex Turner) C. Agardh, 1820, brown algae. The study result indicated that compounds exhibited inhibitory activity toward Na+/K + ATPase from the porcine cerebral cortex in a study by Chung et al. (2011).

### Isocitrate lyase inhibitory activity of meroterpenoids

Chung et al. isolated chromene class meroterpenoids, namely, sargachromanols L, M, and P, from the brown alga *Sargassum siliquastrum* (Mertens ex Turner) C. Agardh, reporting that compounds exhibited moderate ICL inhibitory activity (Chung et al., 2011).

### Chenodeoxycholic acid-activated human farnesoid X receptor activity of meroterpenoids

Choi et al. isolated meroterpenoids tuberatolides A and B, 2′-*epi*-tuberatolide B, yezoquinolide, (*R*)-sargachromenol, and (*S*)-sargachromenol from the Korean marine tunicate *Botryllus tuberatus* Ritter & Forsyth, 1917*.* In a cotransfection cell-based assay, these compounds without significant cytotoxicity showed potent inhibition of hFXR transactivation. Also, tuberatolide A at low concentrations antagonized chenodeoxycholic acid- (CDCA-) dependent activation of hFXR without any cytotoxicity in both bioassay systems (Choi et al., 2011).

### Mammalian mitochondrial respiratory chain inhibitory activity of meroterpenoids

Two meroterpenoids, terretonins E and F, along with the known compound aurantiamine, was isolated as fermentation products of the marine fungus *Aspergillus insuetus* (Bainier) Thom & Church (1929), associated with the sponge *Petrosia ficiformis* (Poiret, 1979)*.* Meroterpenoids, terretonins E and F, showed potential inhibition of the integrated chain (NADH oxidase activity; also, aurantiamine was five times less potent than terretonin F (López-Gresa et al., 2009).

### Hypoxia-inducible factor-1 inhibitory activity of meroterpenoids

Meroterpenoids, bisbakuchiols A–C, 12,13-dihydro-12,13-dihydroxybakuchiol, 12,13-dihydro-12,13-epoxybakuchiol and O-methyl, and O-ethyl bakuchiols, were isolated from the seeds of *Psoralea corylifolia* L. (Fabaceae) in a study by Wu et al. The result displayed that all compounds exhibited an HIF-1 inhibitory effect (Wu et al., 2008). In a similar study, a bioassay-guided phytochemical investigation by Wu et al. of the methanol extract of *P. corylifolia* using a HIF-1-mediated reporter gene assay in human gastric cancer cells led to the isolation of dimeric meroterpenoid (S)-bakuchiol inhibited hypoxic activation of HIF-1 with an IC_50_ value of 6.1 µM (Wu et al., 2007).

### Larvicidal activity of meroterpenoids

Geris et al. conducted a study to determine the potential of larvicidal activity of meroterpenoids, dehydroaustin, acetoxydehydroaustin, and austin from *Penicillium* sp. against third-instar larvae of *A. aegypti.* The results showed that when the meroterpenoids at a concentration of 500 ppm each were exposed to third-instar larvae of *A. aegypti*, meroterpenoids dehydroaustin and acetoxydehydroaustin exhibited *in vitro* larvicidal activity of 100% and 70%, respectively, after 24 h of exposure and austin displayed a very low larval mortality compared with positive control temephos (Geris et al., 2008).

### Anti-invasion activity of meroterpenoids

Meroterpenoids, namely, avinosol, avarone, avarol, and avinosone, were isolated from *Dysidea* sp. marine sponge collected in Papua New Guinea in a study by Marrero et al. The meroterpenoids were tested in the anti-invasion assay against MDA-MB-231 breast cancer cell lines and LS174T colon carcinoma cells. It was found that avinosol had an IC_50_ of ∼50 μg/ml in the anti-invasion assay against both cell lines. Avarone, avarol, and avinosone were only active in the assay at a concentration of 100 μg/ml (Diaz-Marrero et al., 2006).

### Protein kinase MK2 inhibitory activity of meroterpenoids

Williams et al. isolated (+)-makassaric acid and (+)-subersic acid, new meroterpenoid inhibitors of the protein kinase MK2m from the marine sponge *Acanthodendrilla* sp. The study concluded that (+)-makassaric acid and (+)-subersic acid inhibited MK2 with IC_50_ of 20 and 9.6 µM, respectively (Williams et al., 2004).

### Antibiofilm activity of meroterpenoids

From the leaves of *E. robusta*, eucarobustol E (EE) meroterpenoid was isolated*.* The results showed strong inhibitory activity against *C. albicans* biofilms with 16 *μ*g/ml concentration. The study concluded that EE blocked yeast-to-hypha transition and thus reduced cellular surface hydrophobicity cells of biofilm (Liu R. H. et al., 2017).

### Phosphodiesterase-4 inhibitory activity of meroterpenoids

The isolation of *Psidium* meroterpenoids psiguajadials A–K was triggered by bioassay-guided fractionation of the ethanolic extract of *Psidium guajava L.* leaves, guajavadial C, psiguadial D, psiguadial A, guapsidial A, psidial A, guajadial, psiguajadial L, guajadials C–F, guajavadial A, and guadial A. The isolated compounds exhibited moderate inhibitory activity with IC_50_ values in the range of 1.34–7.26 *μ*M compared with positive control rolipram (Tang et al., 2017).

### Increase in intracellular free calcium activity of meroterpenoids

From the *Ganoderma petchii* (Lloyd) Steyaert (1972) fruiting bodies, Gao et al. isolated petchienes B and (-) D. Outcomes demonstrated that isolated compounds could significantly elevate the concentration of intracellular Ca^2+^ at 10 μM in HEK-293 cells (Gao et al., 2015).

### Effect of meroterpenoids in dermatological diseases

3,5-Dimethylorsellinic acid- (DMOA-) related meroterpenoids, namely, brasilianoids A–E were isolated, from the fungus *Penicillium brasilianum* Bat. WZXY-m122-9 ethyl acetate extract. Compound brasilianoid A significantly increased the expression of caspase-14 and filaggrin in HaCaT cells in a dose-dependent manner., The cytotoxicity of brasilianoid A against HaCaT cells was measured by the MTT assay to test the skin protective activity against UVB irradiation. After exposure to UVB 30 mJ/cm^2^, cell viability was decreased to 70% compared to the normal group. Brasilianoid A (20 μM) treated the damaged cells, increasing cell viability to 77% compared with positive control epigallocatechin gallate. NO production in LPS-induced RAW 264.7 macrophages was moderately inhibited by meroterpenoids, namely, brasilianoids B and C. In addition, brasilianoids C–E (10 μM) also resulted in the inhibition of DNA expression of the HBV virus in HepG2.2.15 cells with the inhibition rates of 25%, 15%, and 10%, respectively, the same as that of lamivudine (positive control) (Zhang J. et al., 2018).

### Phytotoxic activity (plant toxicity) of meroterpenoids

Ma et al. isolated guignardianone C from the fermentation extract of *Phyllosticta capitalensis* Henn., (1908). The phytotoxic effects of guignardianone C on *Lactuca sativa* L. and *Lolium perenne* L. were evaluated. Guignardianone C displayed inhibition activity on the shoot growth of *L. sativa* and *L. perenne* and the root growth of *L. perenne* (Ma et al., 2019).

### Growth inhibition activity of meroterpenoids against newly hatched larvae of *Helicoverpa armigera* (Hübner, 1808)

Bai et al. isolated bioactive metabolites from mangrove-derived fungal *Penicillium* sp. (penicianstinoids A and B; peniciisocoumarins A, B, E, F, and H; austinol; 1,2-dihydro-7-hydroxydehydroaustin; and austin). These were reported to have growth inhibitory activity with IC_50_ values between 50 and200 μg/ml, respectively (Bai et al., 2019).

## Summary

Meroterpenoids are a group of partially derived secondary metabolites from terpenoid biosynthetic pathways. They exhibit huge structural diversity, from basic compounds containing a prenyl unit to more complex meroterpenoids formed with functionalized carbon chains. Meroterpenoids and their derivatives are isolated from natural resources, such as seeds, animals, fungi, and marine organisms. They have been rigorously subjected to pharmacological screening and possess a broad spectrum of pharmacological activities. More than 190 meroterpenoids reported here were isolated from different species of fungi, such as *Penicillium*, *Aspergillus*, *Ganoderma*, and *Sargassum*, and have shown anticancer, anti-proliferative, anti-viral, anti-microbial, anti-inflammatory, anti-Alzheimer’s, and anti-obesity activities. Similarly, algal-based meroterpenoids isolated from algae species such as *Cystoseira*, *Sargassum*, and *Hypericum* have shown anti-oxidant, anti-microbial, anti-proliferative, and cytotoxic activity. Species of *Ganoderma*, *Eucalyptus*, *Cordial*, *Rhododendron*, and *Psidium* are primary sources of plant-based meroterpenoids active against HIV, leishmaniasis, diabetes, fungal, and bacterial infections and Alzheimer’s and cancer progression. More than 80 meroterpenoids were isolated from marine sources, such as seaweeds, clam, sponges such as *Dactylospongia*, Okinawan, Chilean, actinomycetes, and *Penicillium*. Species have reported pharmaco-biological activities such as anti-inflammatory, cytotoxicity, gastroprotective, anti-viral, antidiabetes, and anti-microbial. Meroterpenoids have also shown activity against alpha-glucosidase, Kaposi-sarcoma associated herpes virus, N-acetyltransferase, BACE1, acetylcholinesterase (AchE), PTP1B, and bromodomain-containing protein 4. They have also demonstrated renoprotective, cardioprotective, and neuroprotective activities. The plethora of research conducted on meroterpenoids from various sources suggests the potential of meroterpenoids being used against the spectrum of diseases and disorders. This review explicitly discusses the nomenclature and isolation of meroterpenoids from different sources and their reported biological activities. The promising range of biological activities and structural complexities exhibited by meroterpenoids make them valuable targets for in-depth study as novel drug candidates.

## Glossary

MAPK: Mitogen-activated protein kinase
LPS: Lipopolysaccharide
NO: Nitric oxide
COX-2: Cyclooxygenase
IL-6: Interleukin-6
IL-10: Interleukin-10
IL-1β: Interleukin-1 β
IL-8: Interleukin-8
PEG2: Prostaglandin G2
TNF-α: Tumor necrosis factor-α
LTB4: Leukotriene B4
NF-κB: Nuclear factor kappa light chain enhancer of activated B cells
5-LOX: 5-Lipoxygenase
Nrf2: Nuclear factor erythroid 2-related factor 2
HO-1: Heme oxygenase 1
PI3K: Phosphoinositide 3-kinase
Akt: Protein kinase B
ABTS- 2,2: Azinobis[3-ethylbenzothiazoline-6-sulfonate]
DPPH- 2,2: Diphenyl-1-picrylhydrazyl
TBARS: Thiobarbituric acid reactive substances
TEAC: Trolox equivalent anti-oxidant capacity
PCL: Photo chemiluminescence
MIC: Minimum inhibitory concentration **IC50**Half maximal inhibitory concentration
MRSA: Methicillin-resistant *Staphylococcus aureus*
FPM: Formyl phloroglucinol meroterpenoid
BACE1: Beta-site amyloid precursor protein cleaving enzyme 1
TGF-β1: Transforming growth factor beta 1
AchE: Acetylcholinesterase
PTP1B: Protein tyrosine phosphatase
ESS: Sargassum serratifolium
H1N: Anti-influenza A virus
MCP-1: Monocyte chemoattractant protein-1
SOD: Superoxide dismutase
CAT: Catalase
GSH-Px: Glutathione peroxidase
MDA: Malondialdehyde
LDH: Lactate dehydrogenase
NSC: Neural stem cell
