# Supplementary material for: Biological activities of meroterpenoids isolated from different sources
Source: Front Pharmacol. 2022 Sep 19;13:830103. doi: 10.3389/fphar.2022.830103 (PMC9527340; doi:10.3389/fphar.2022.830103)
Supplement: Supplementary file 1 [file DataSheet1.docx]

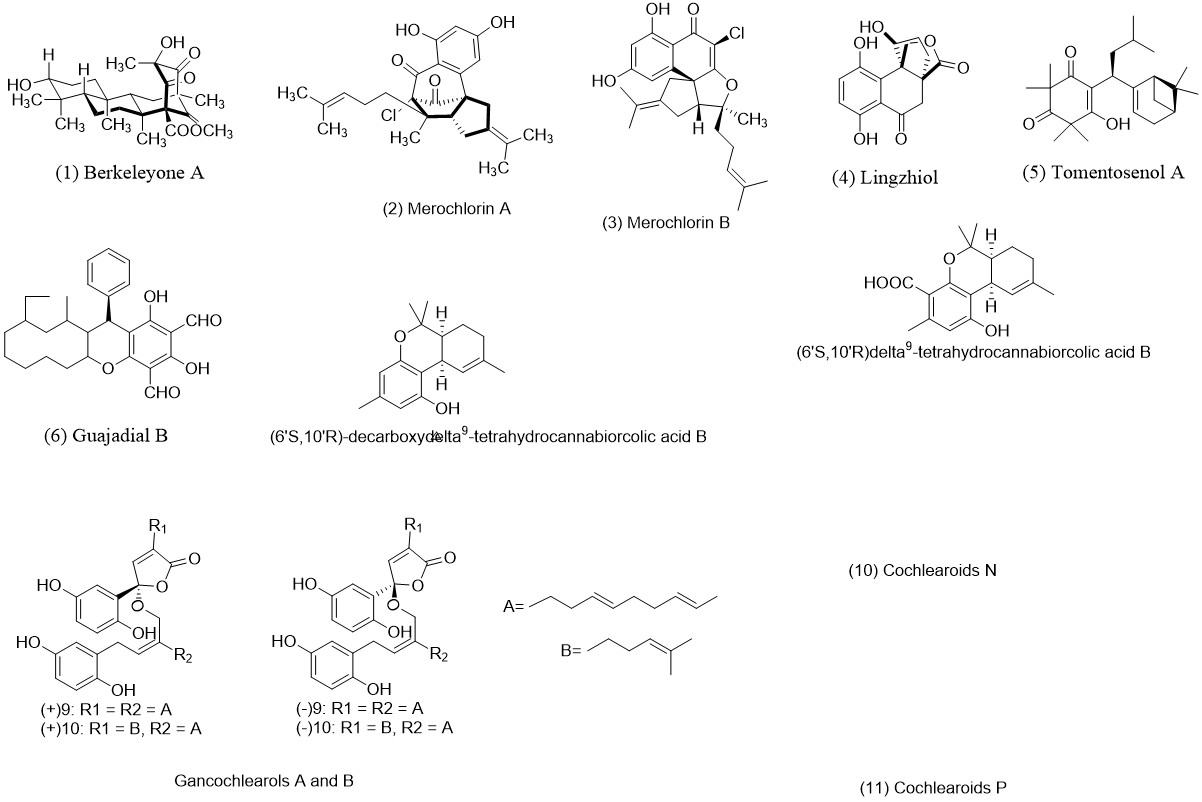

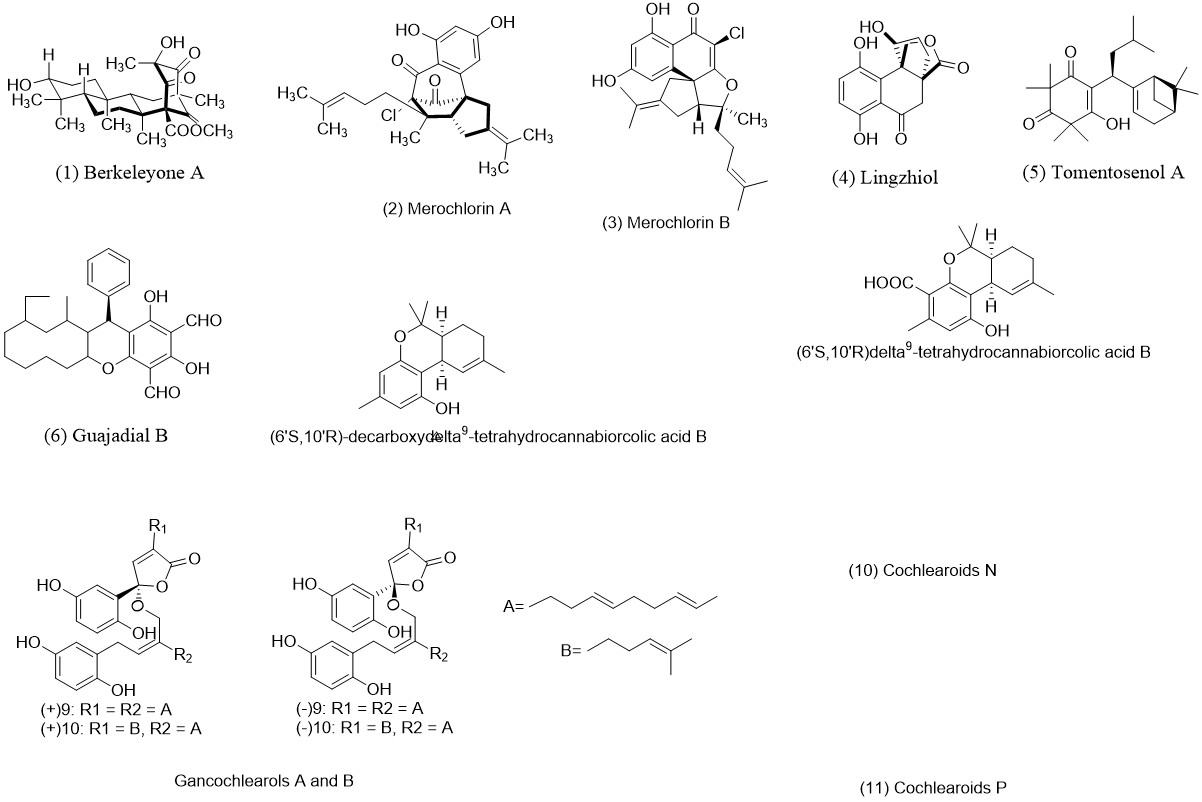

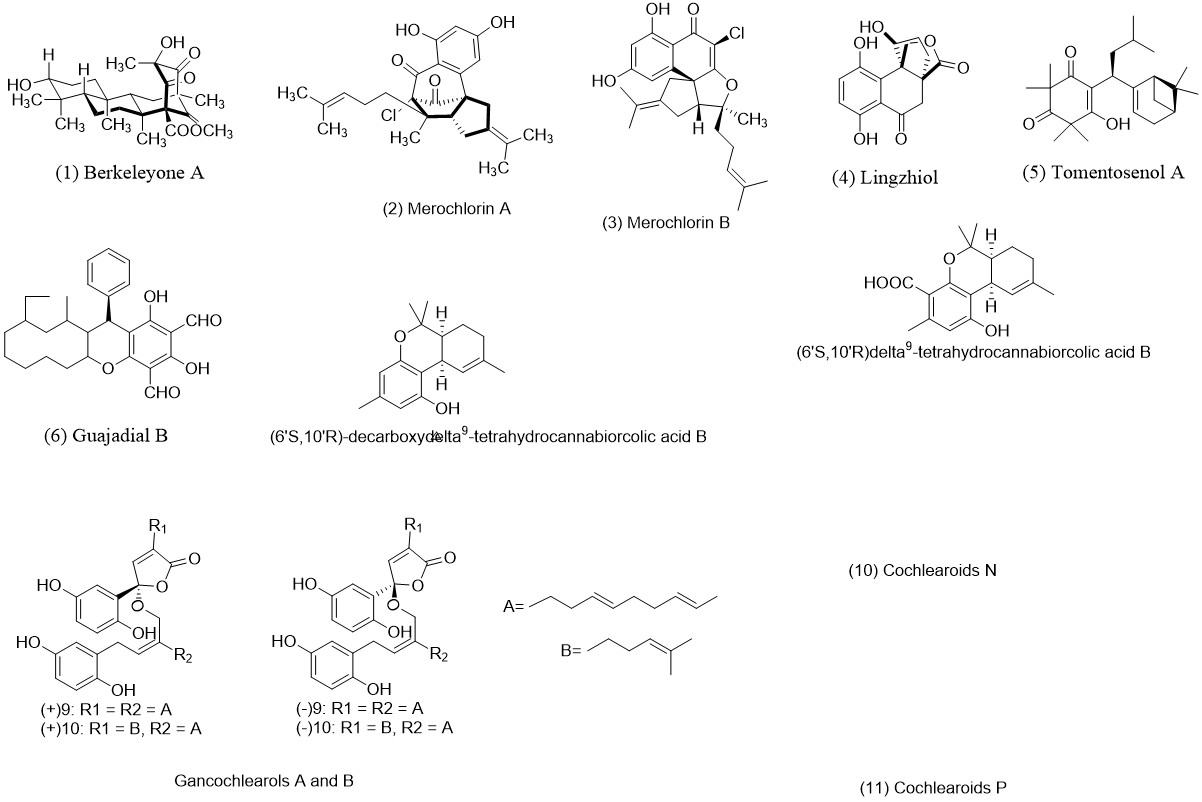

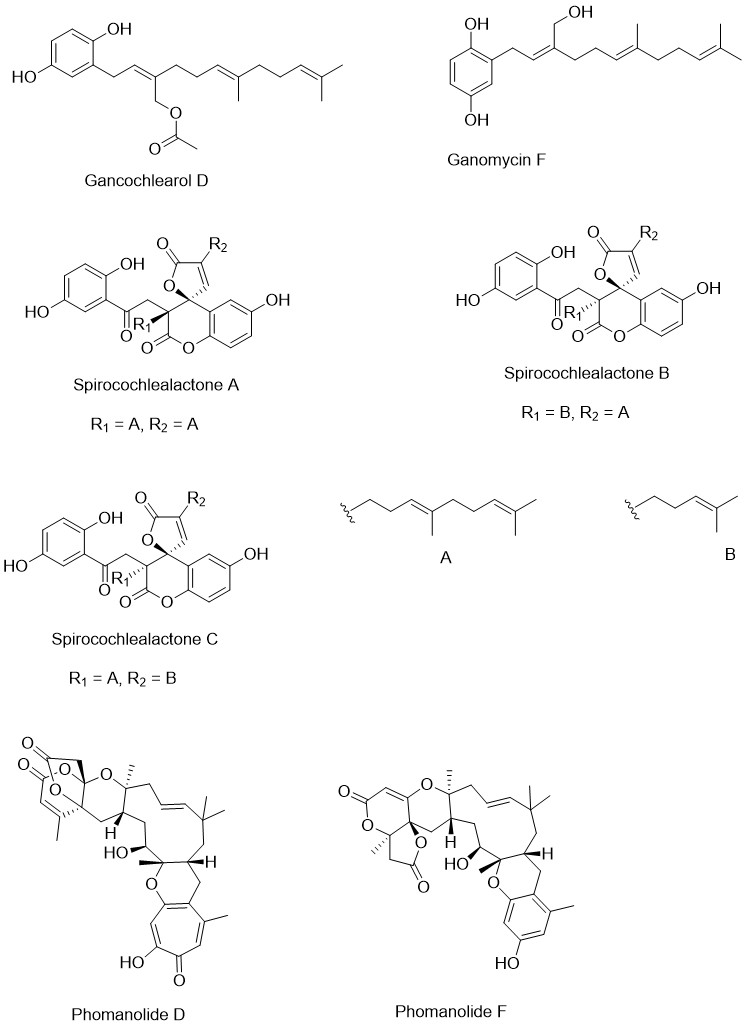


**Fig. No.1: Meroterpenoids showing cytotoxic activity**


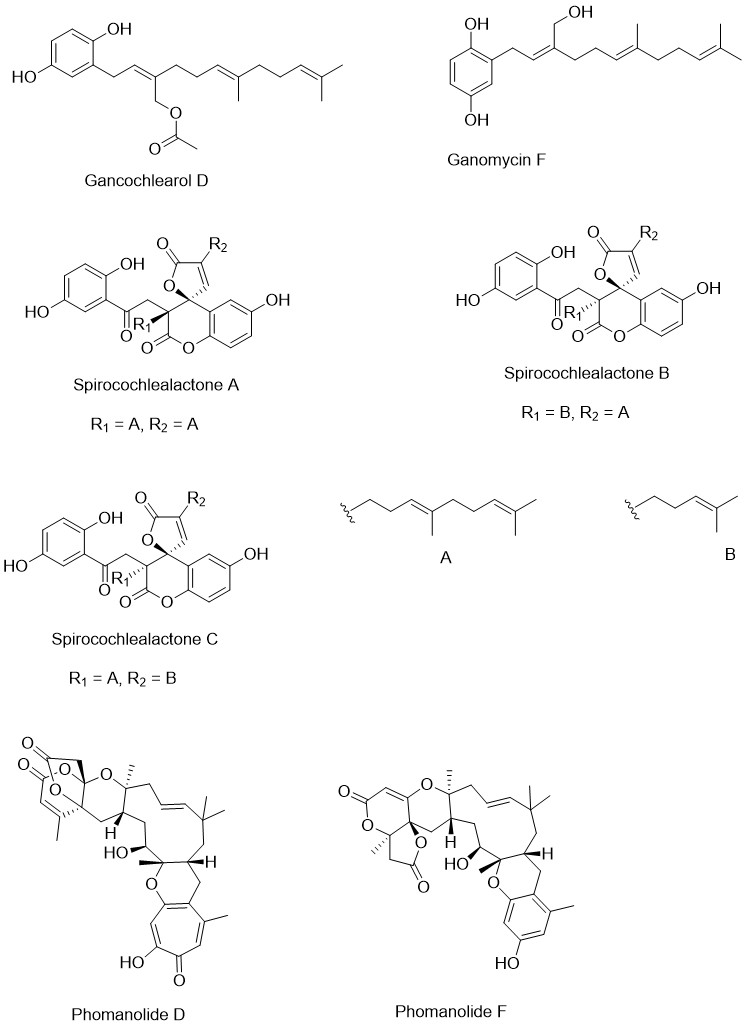

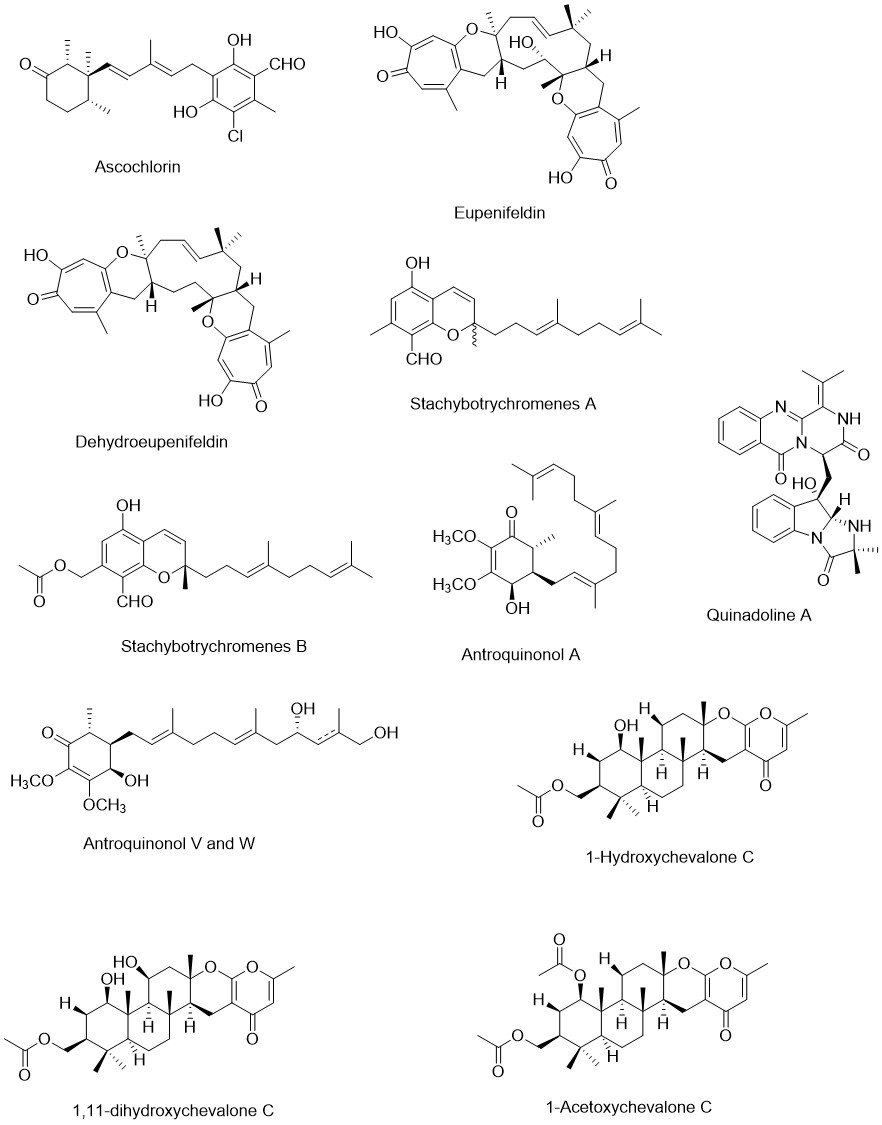


**Fig. No.2: Meroterpenoids showing cytotoxic activity**


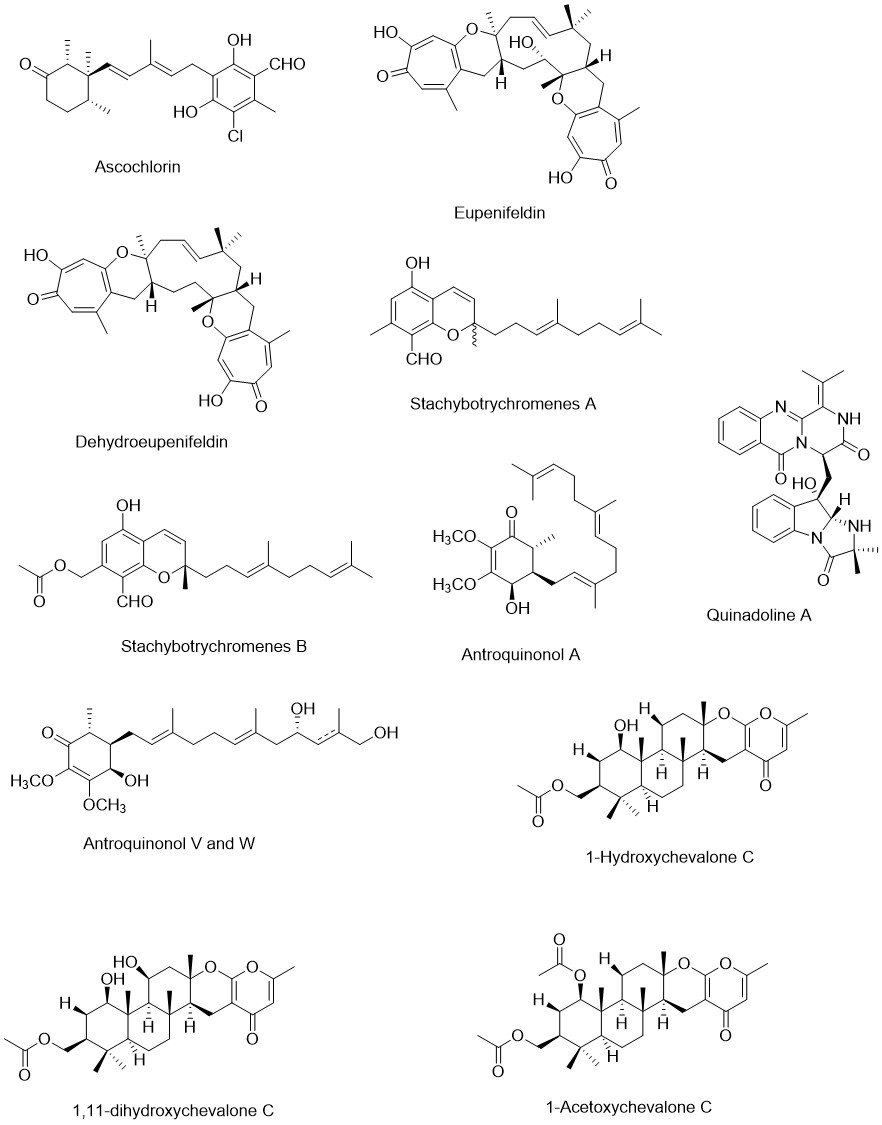

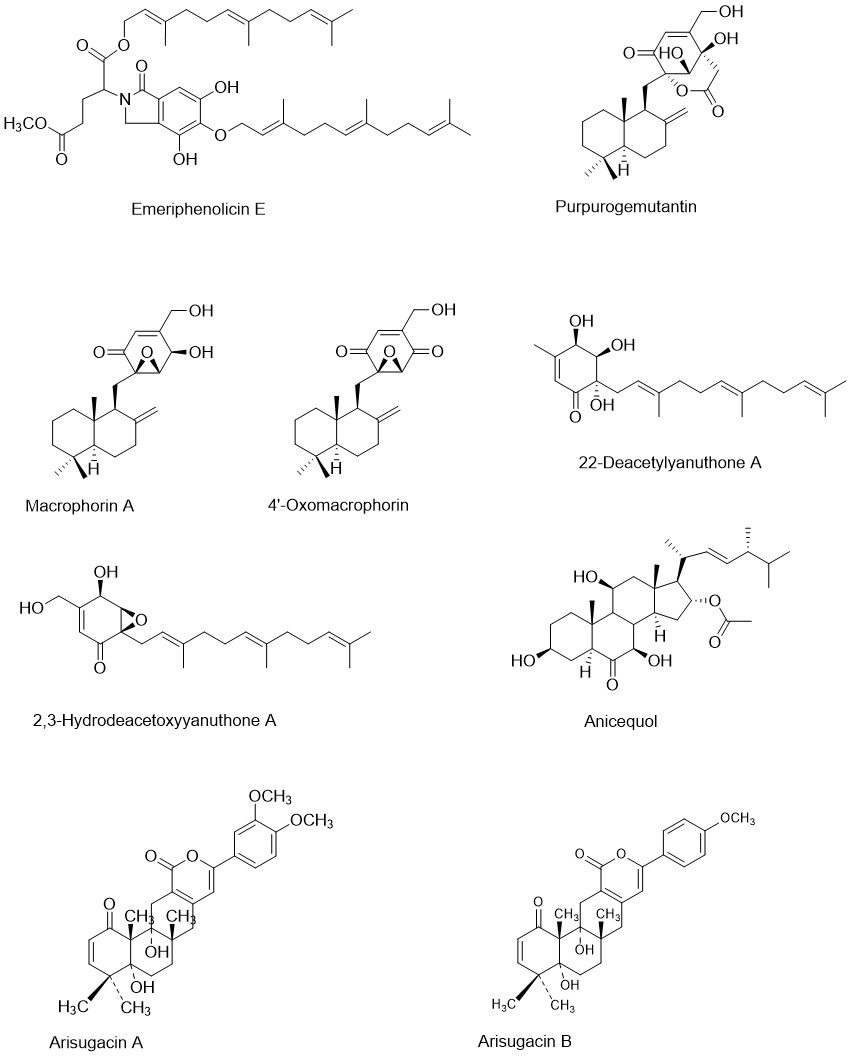


**Fig. No.3: Meroterpenoids showing cytotoxic activity**


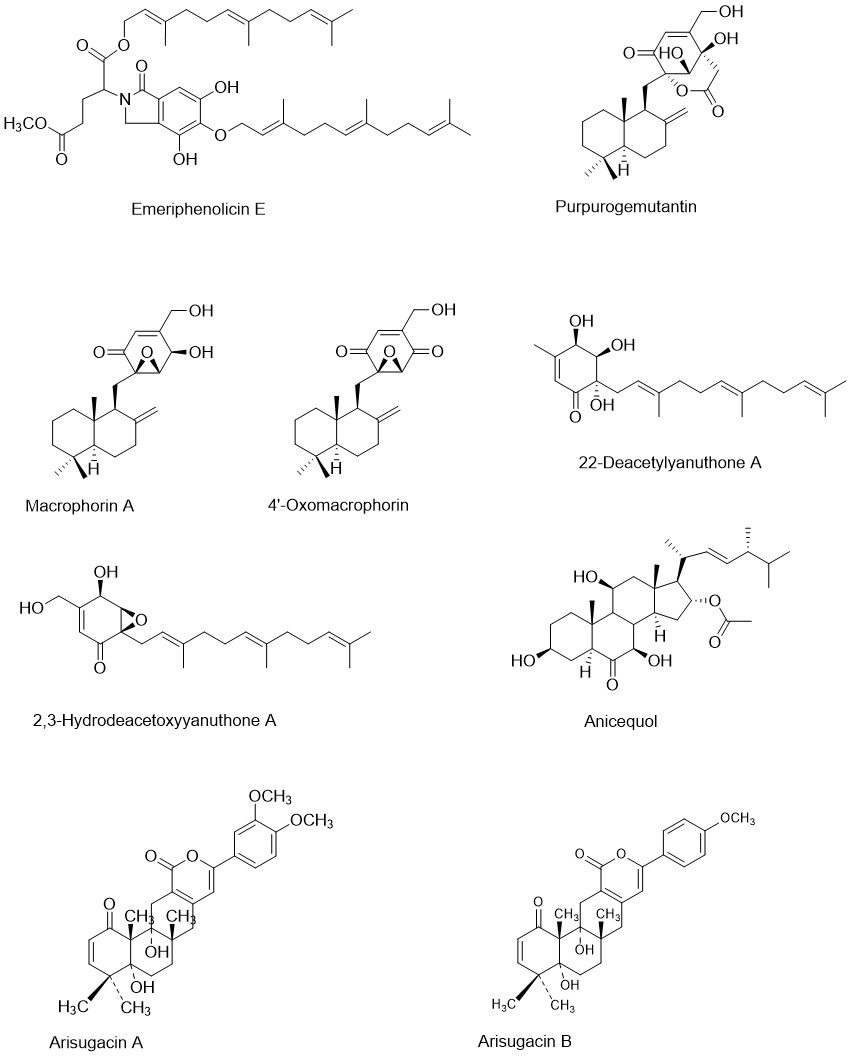

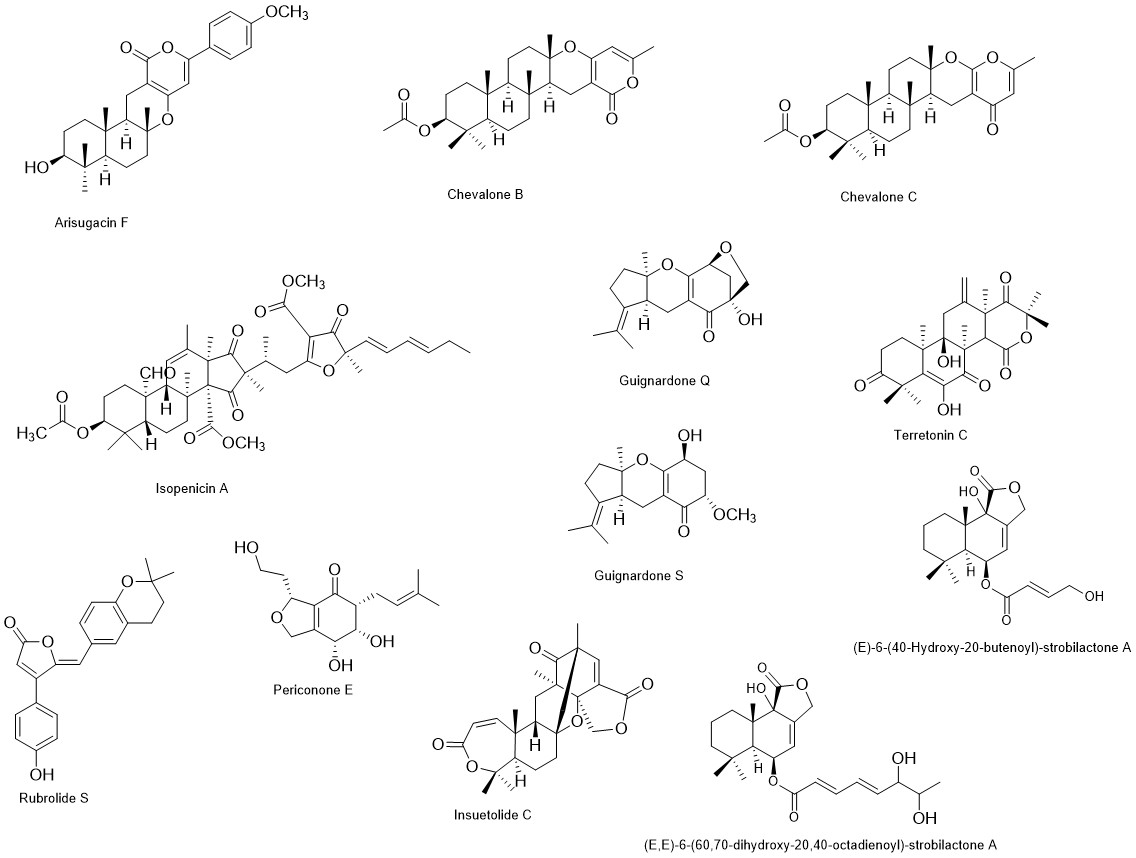


**Fig. No.4: Meroterpenoids showing cytotoxic activity**


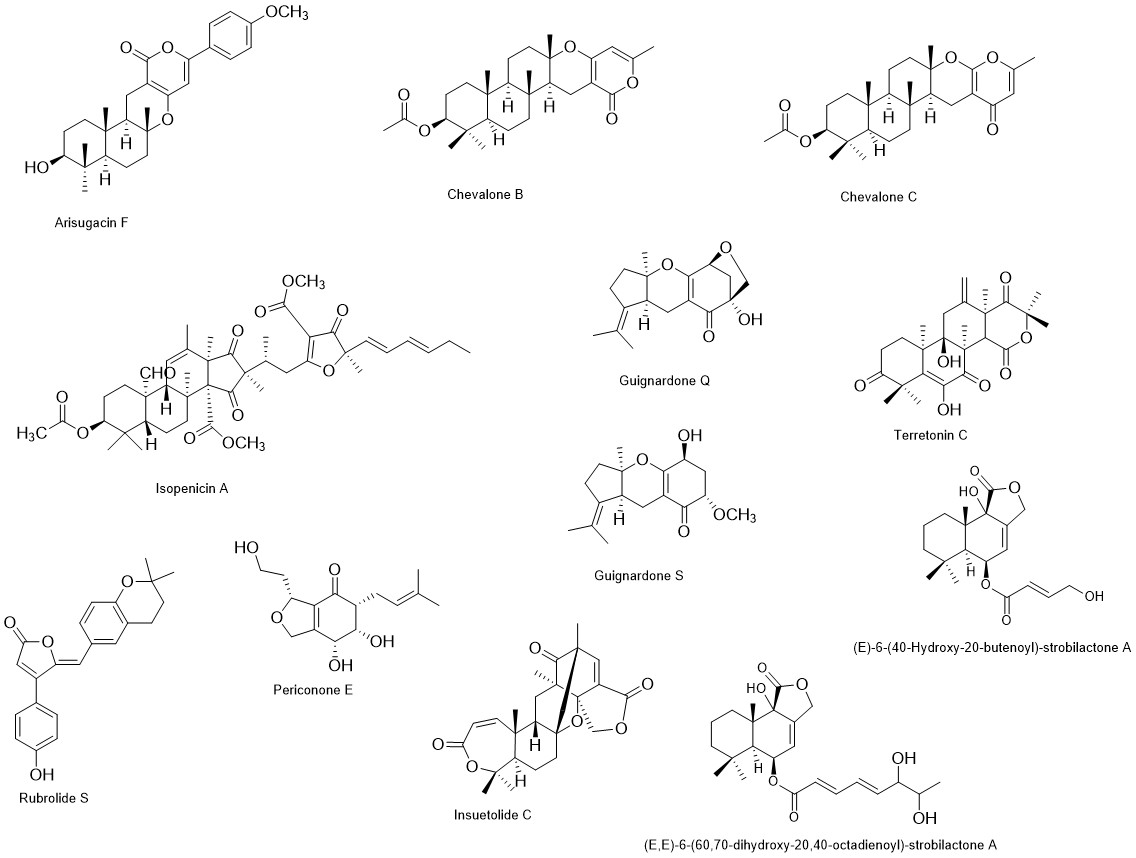

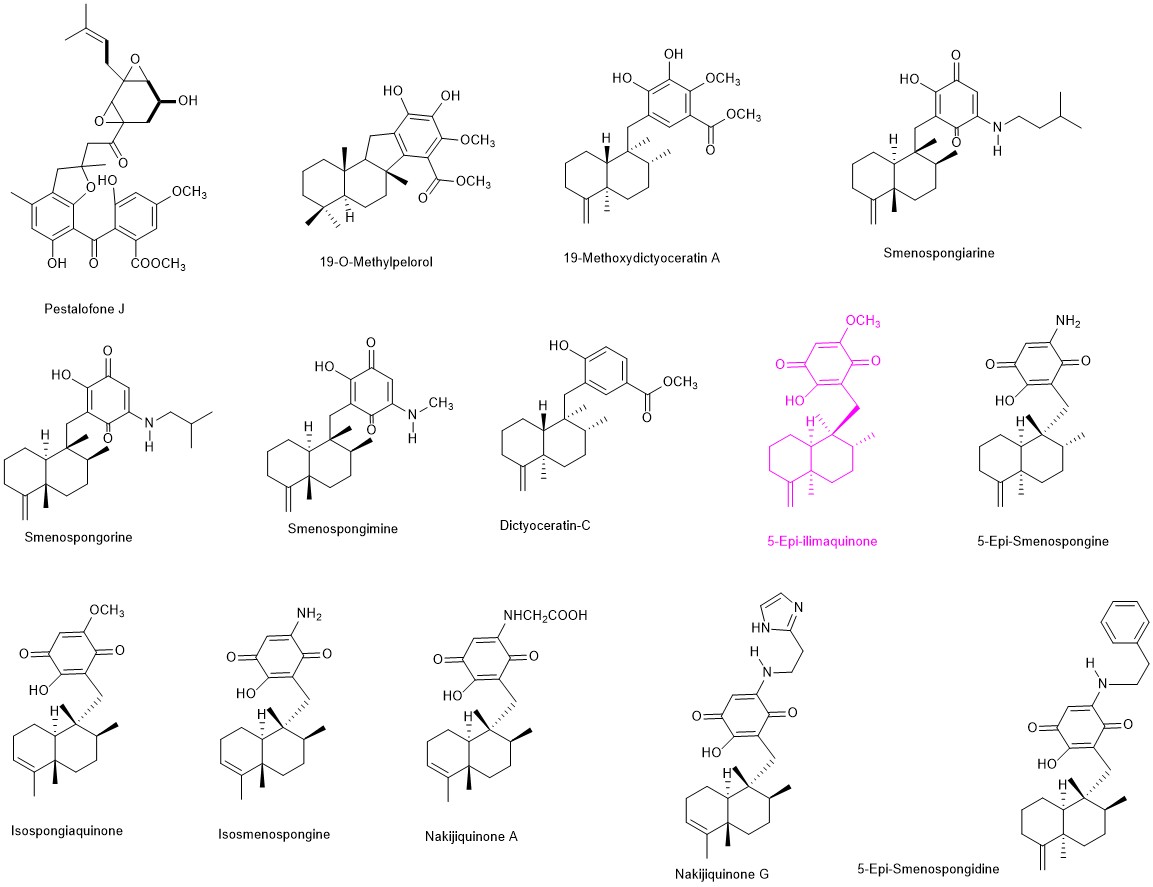

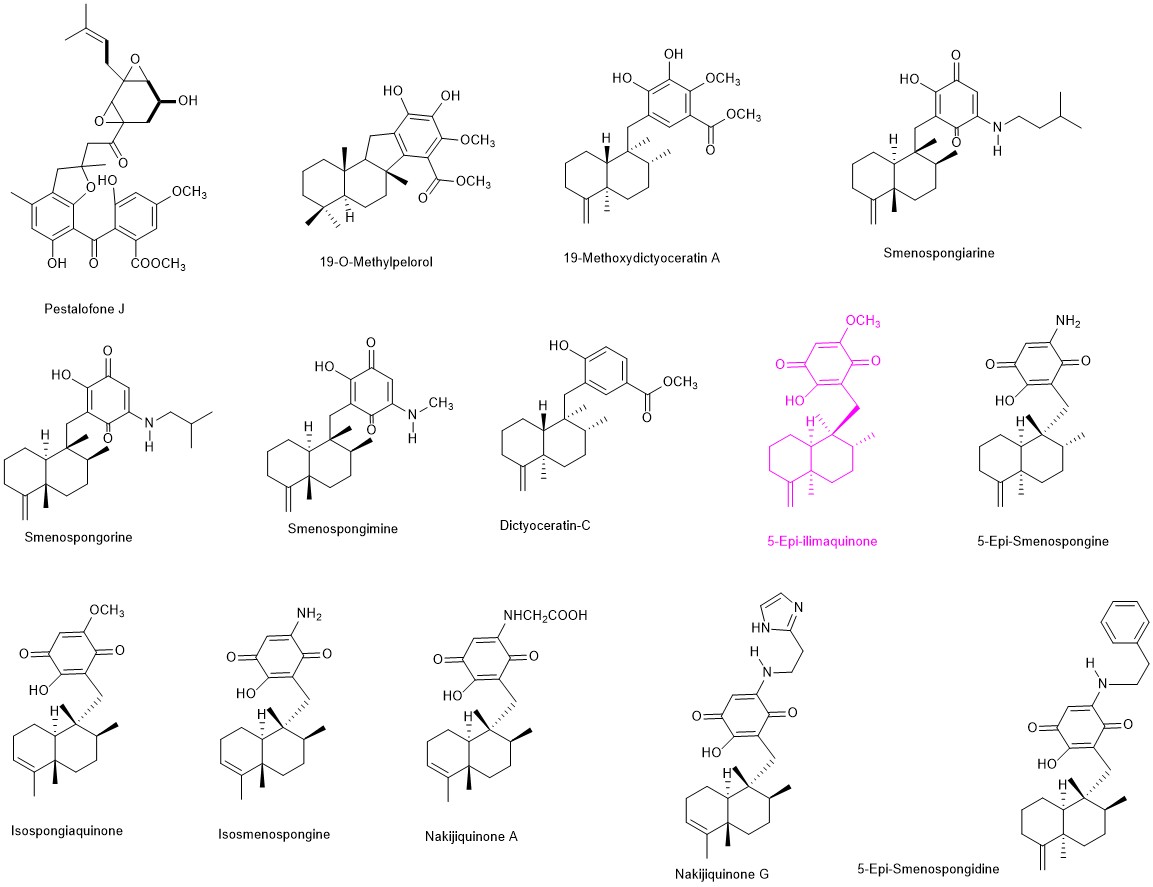

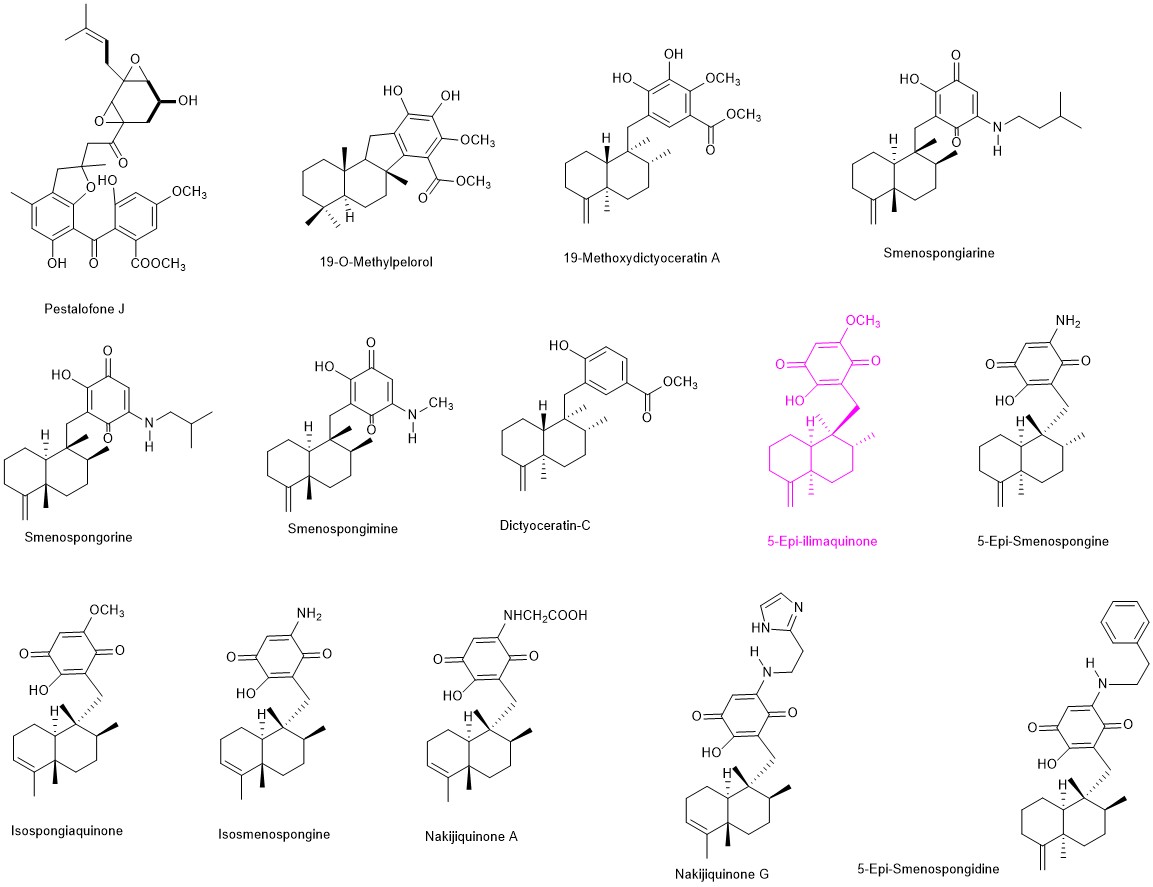

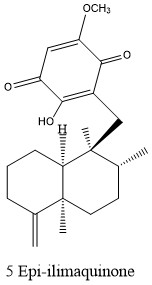


**Fig. No.5: Meroterpenoids showing cytotoxic activity**


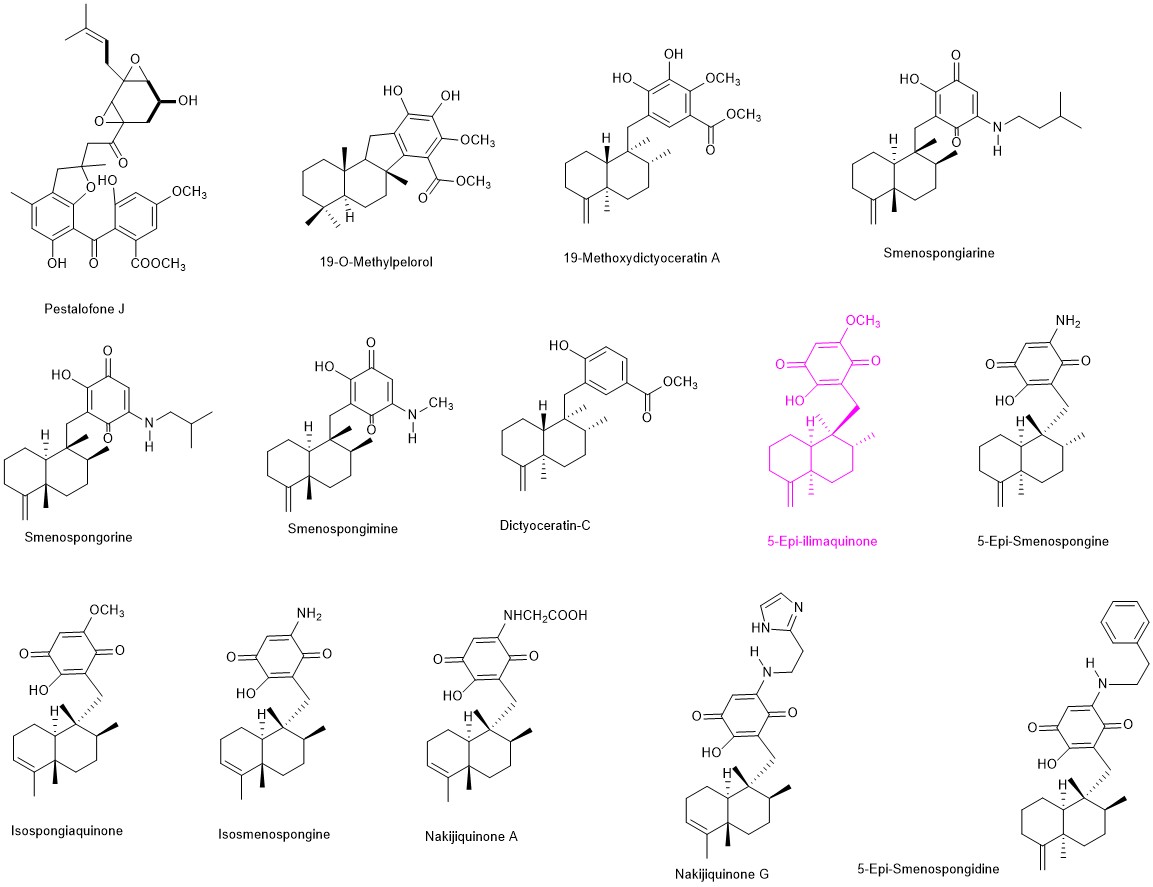

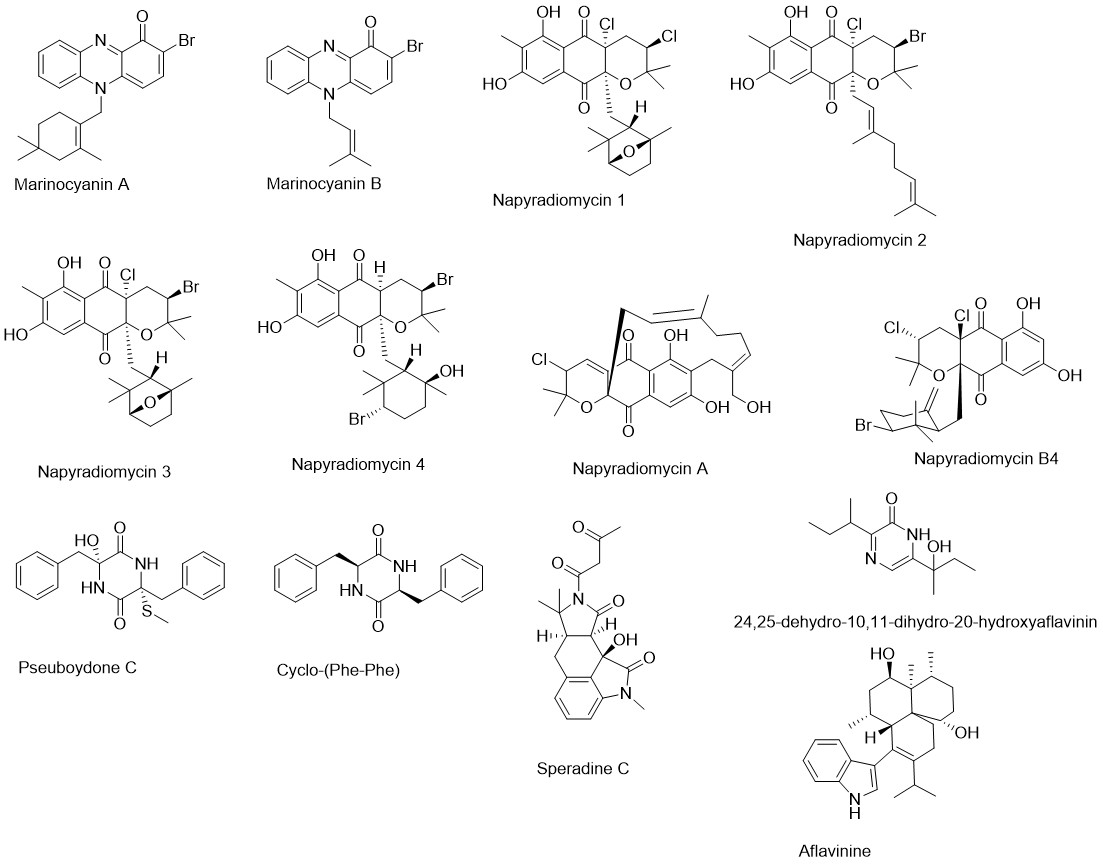


**Fig. No.6: Meroterpenoids showing cytotoxic activity**


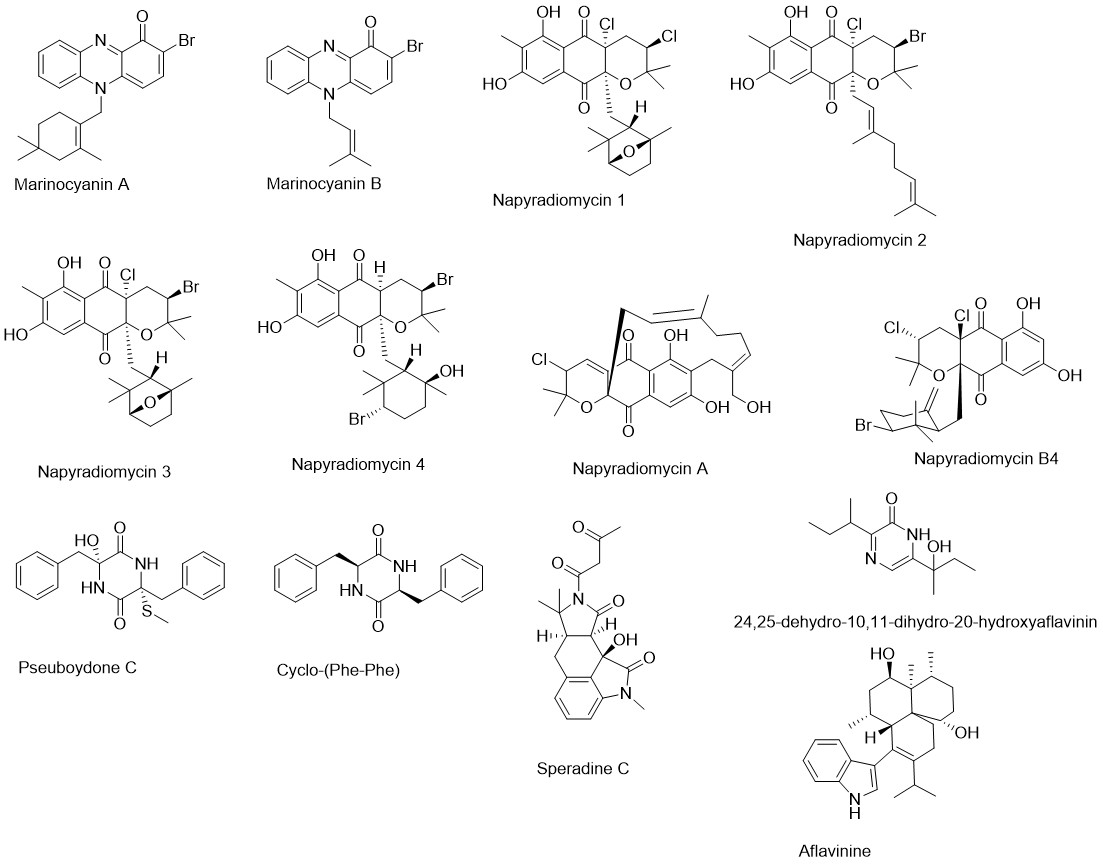

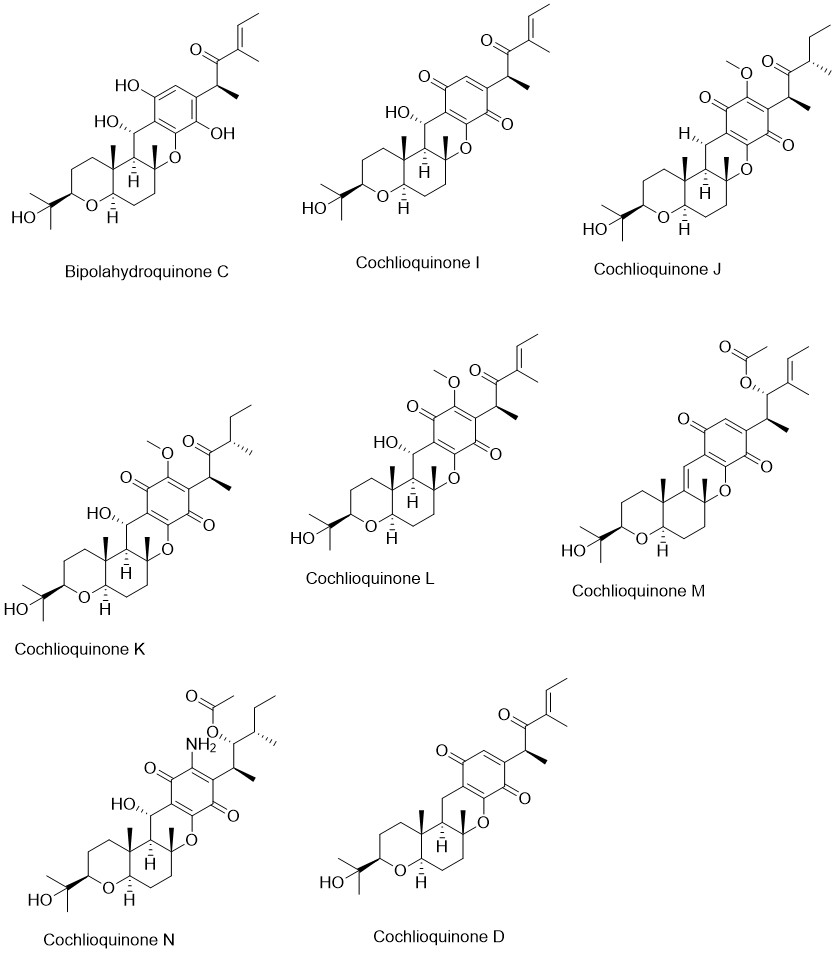


**Fig. No.7: Meroterpenoids showing cytotoxic activity**


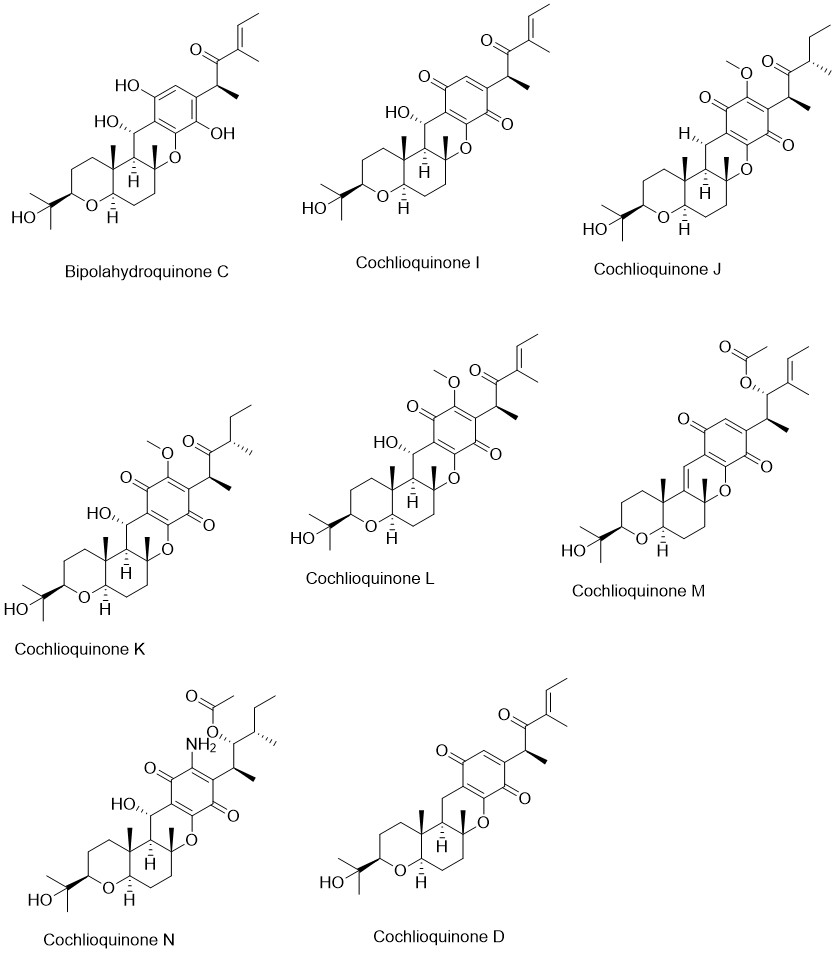

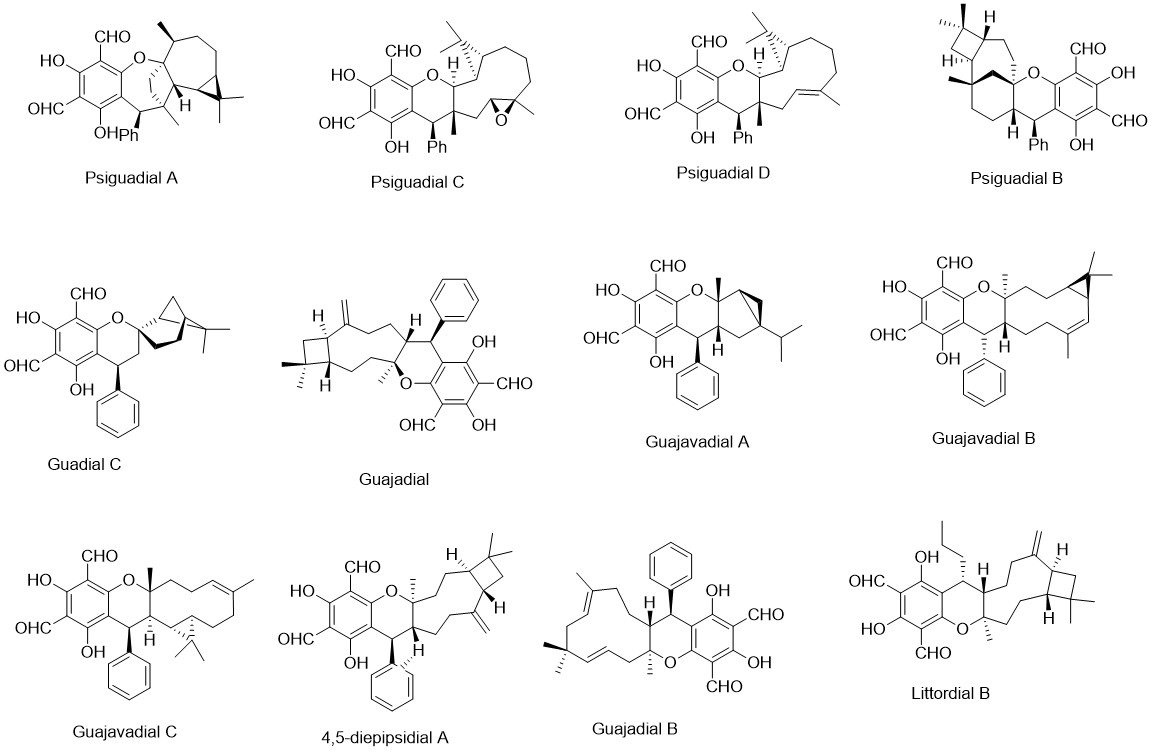


**Fig. No.8: Meroterpenoids showing cytotoxic activity**


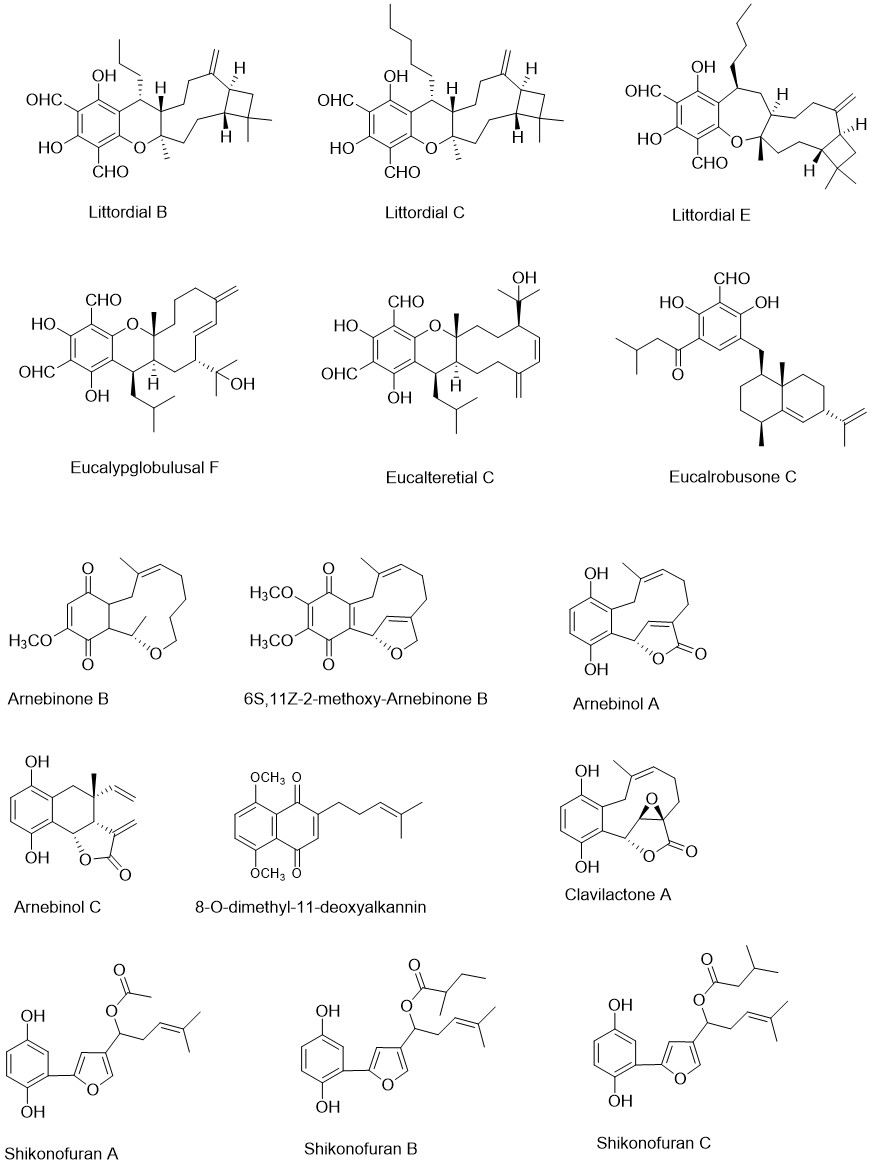


**Fig. No.9: Meroterpenoids showing cytotoxic activity**


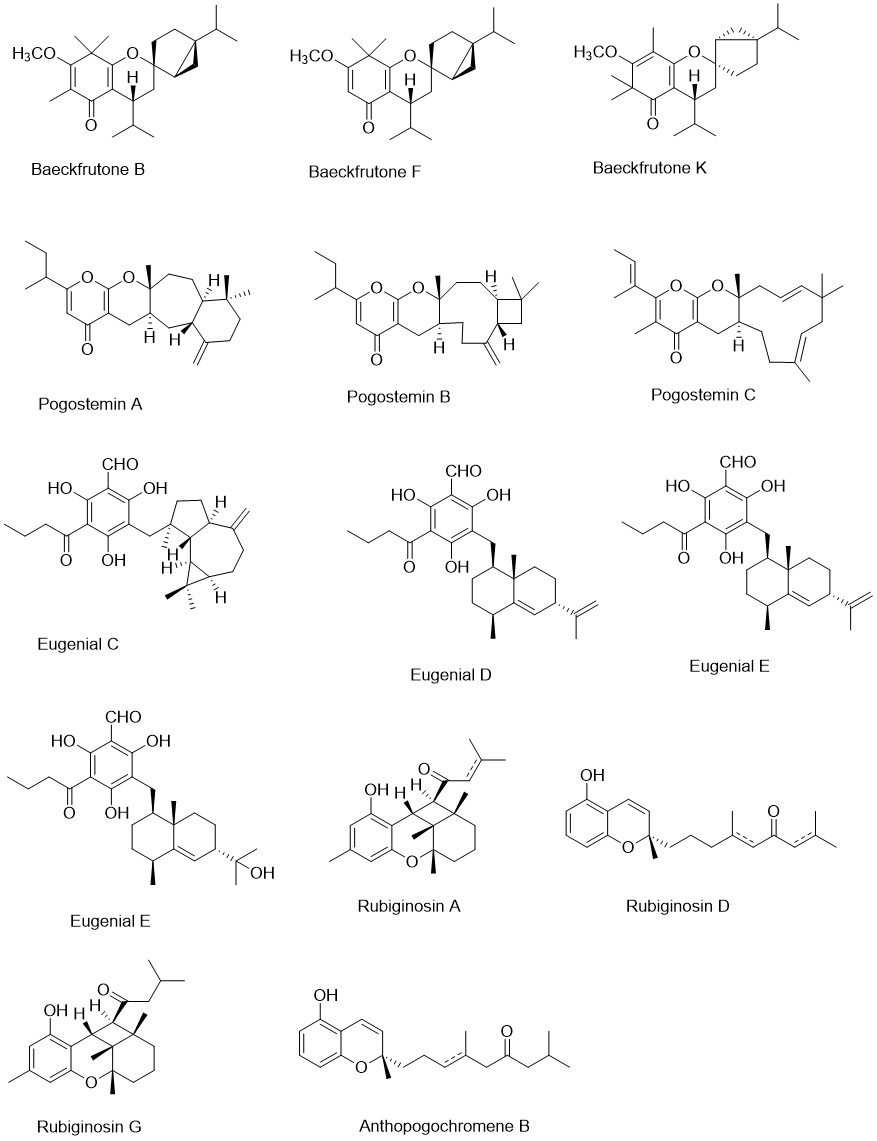


**Fig. No.10: Meroterpenoids showing cytotoxic activity**


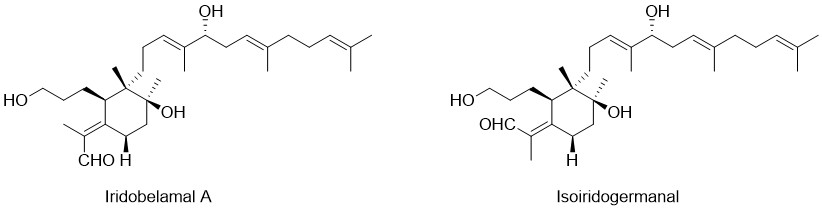

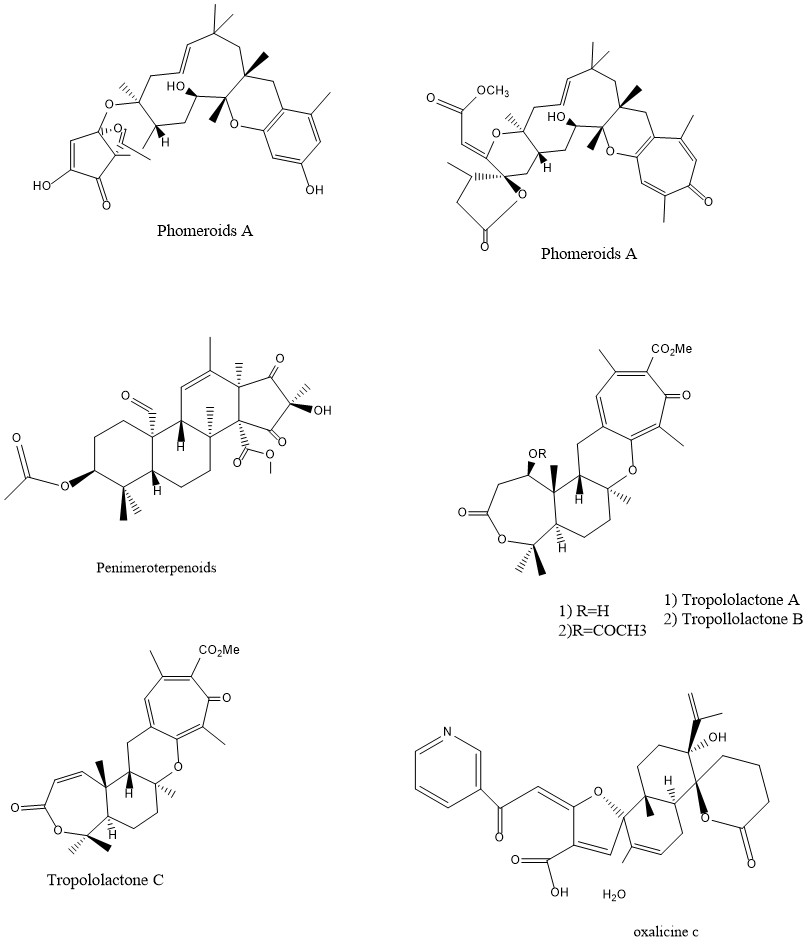

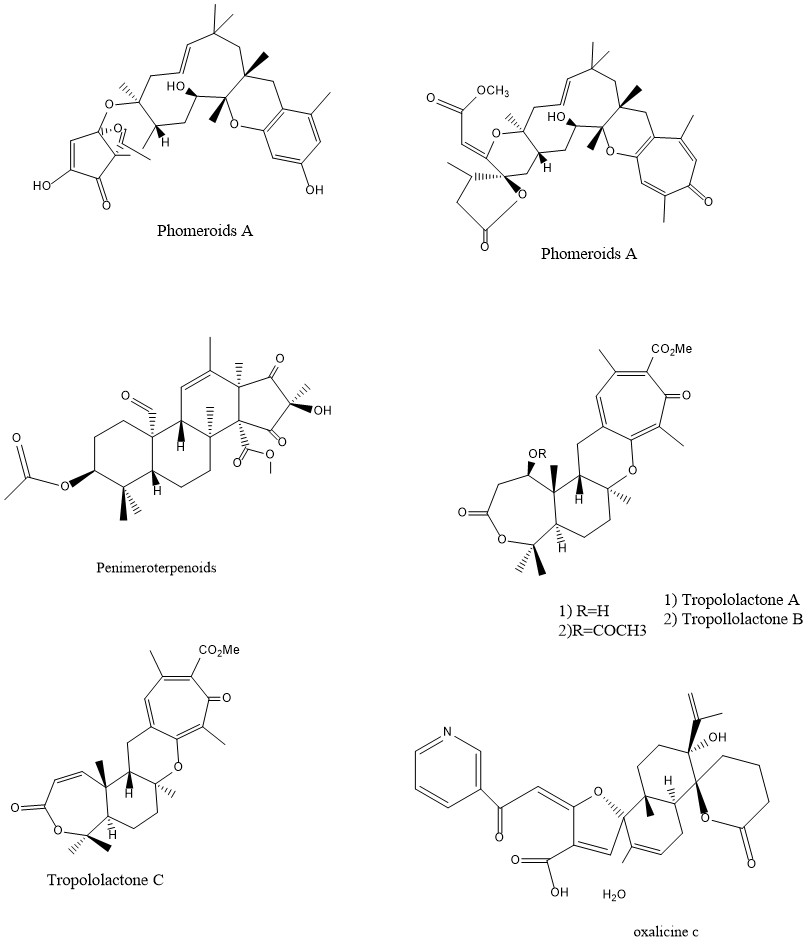


**Fig. No.11: Meroterpenoids showing cytotoxic activity**


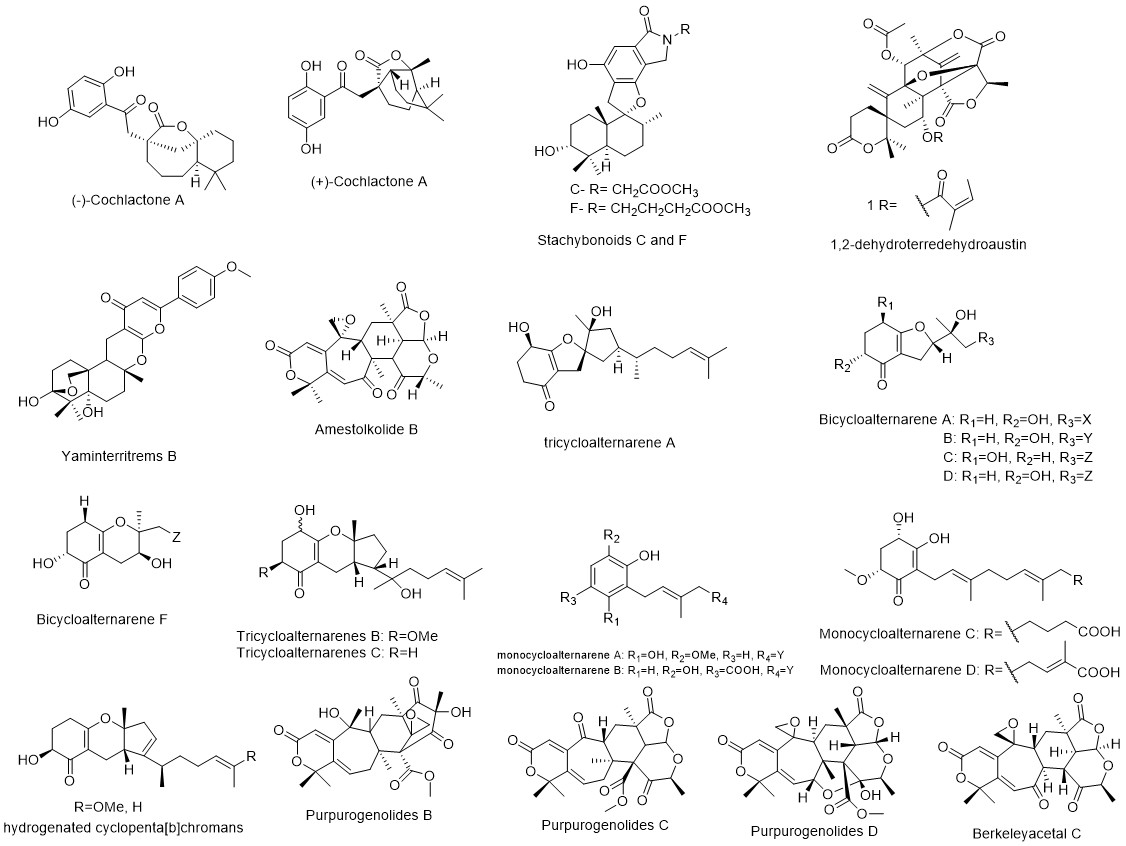


**Fig. No.12: Meroterpenoids showing anti-inflammatory activity**

**Fig. No.13: Meroterpenoids showing anti-inflammatory activity**


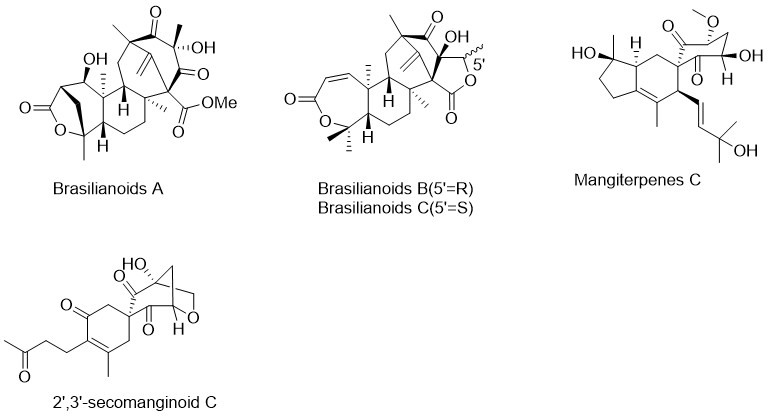

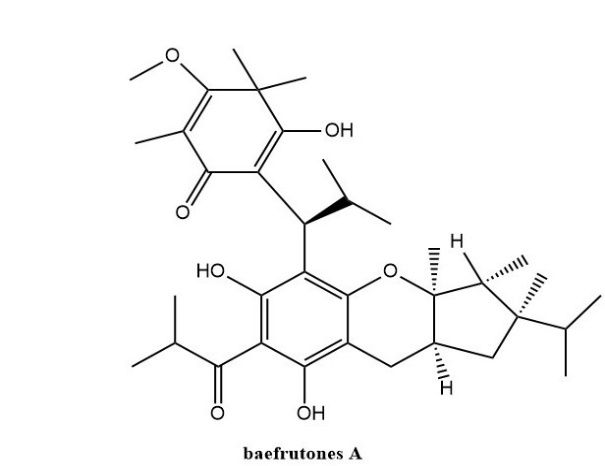

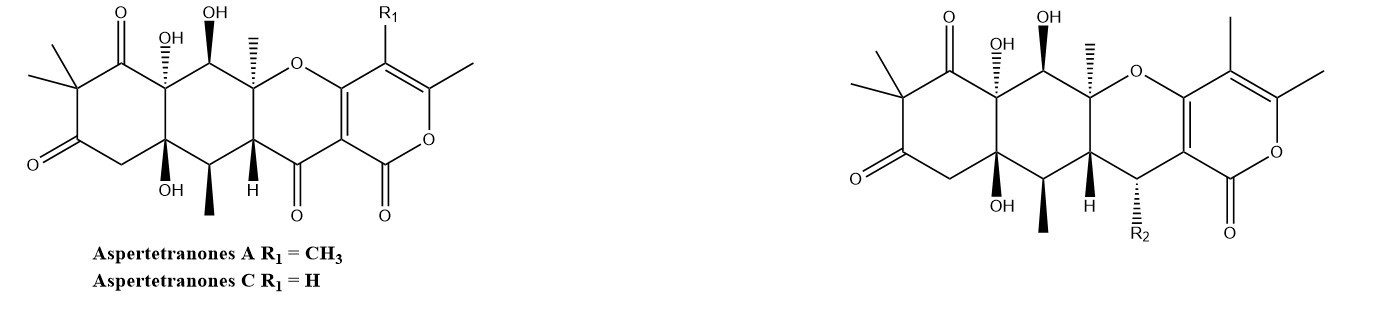

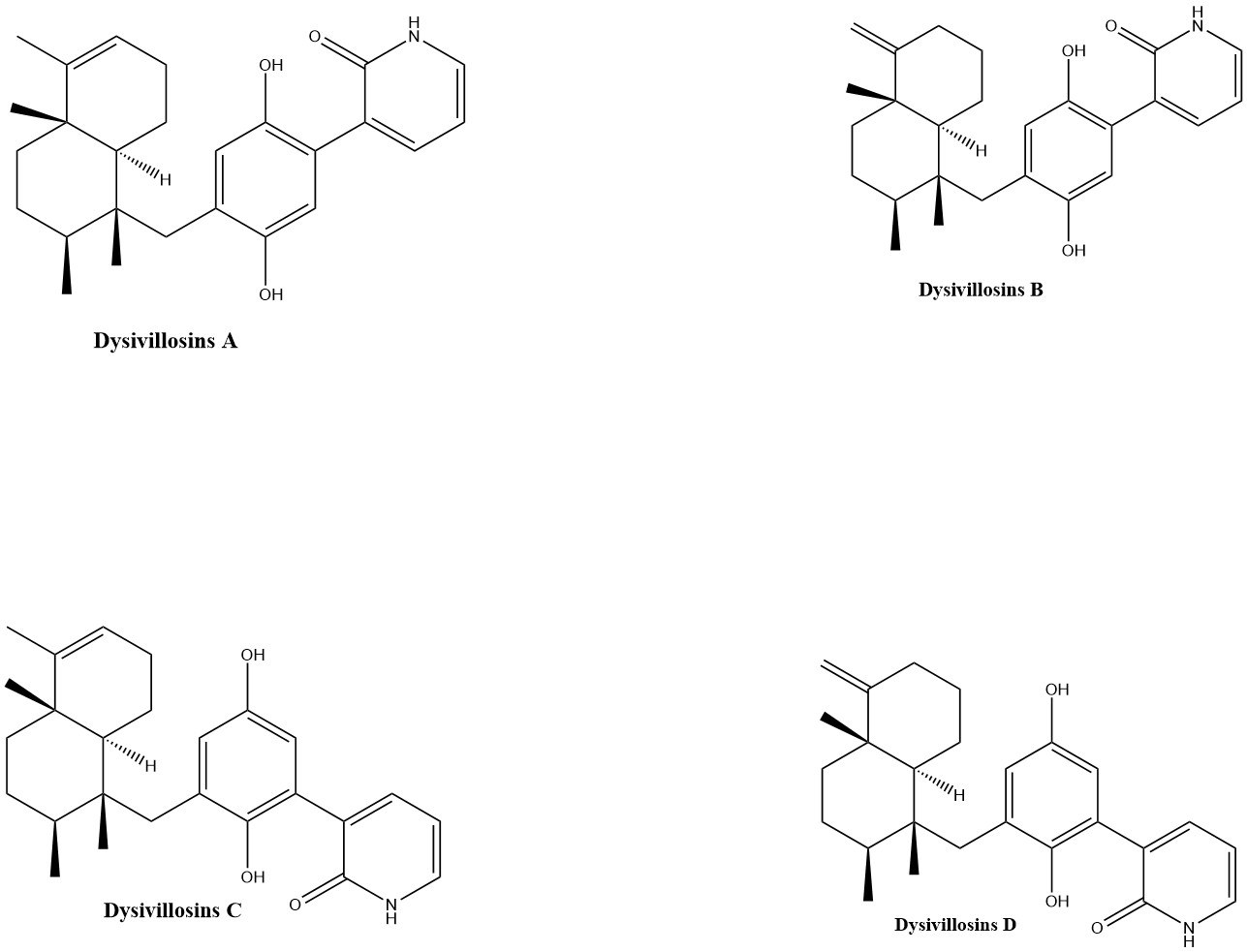

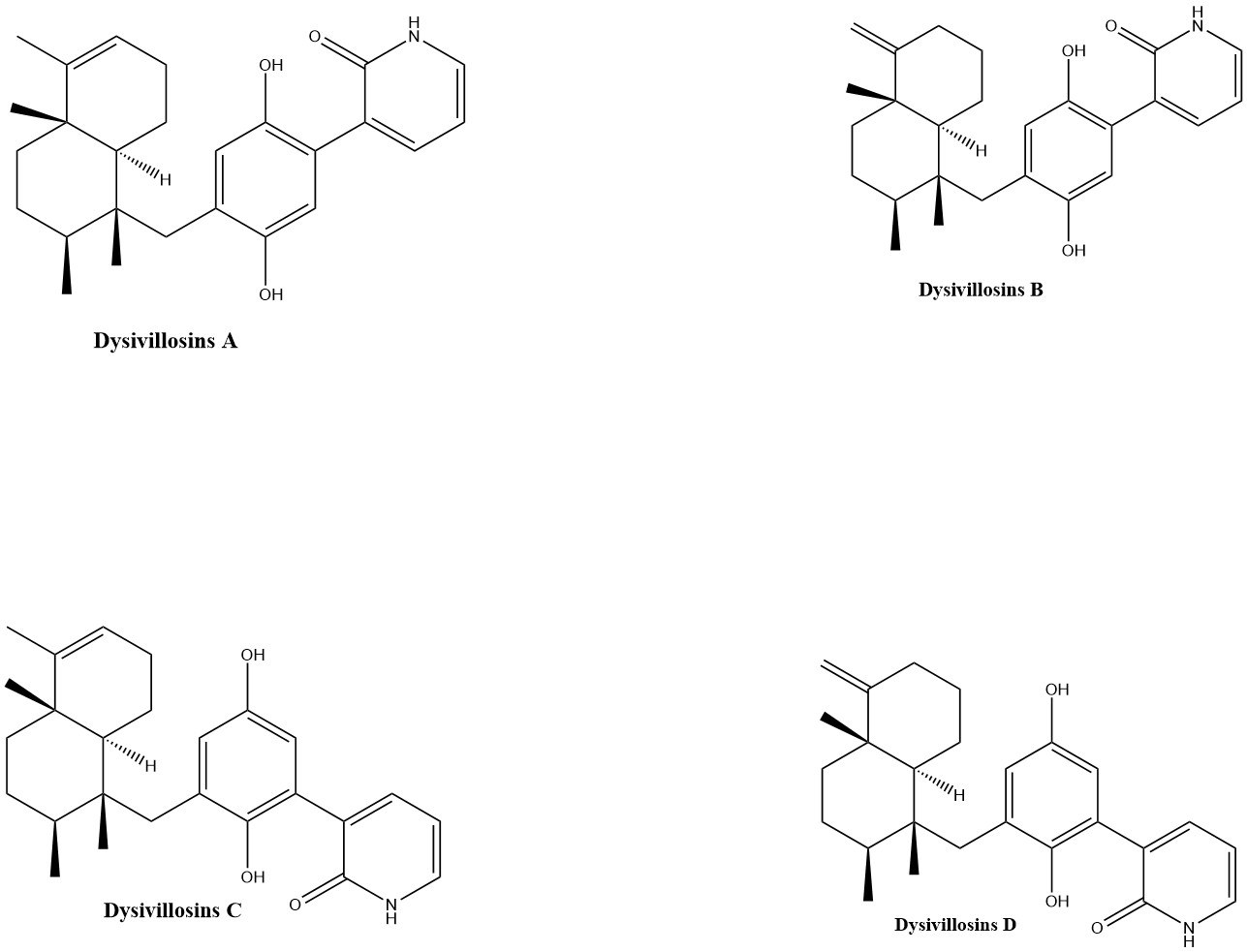

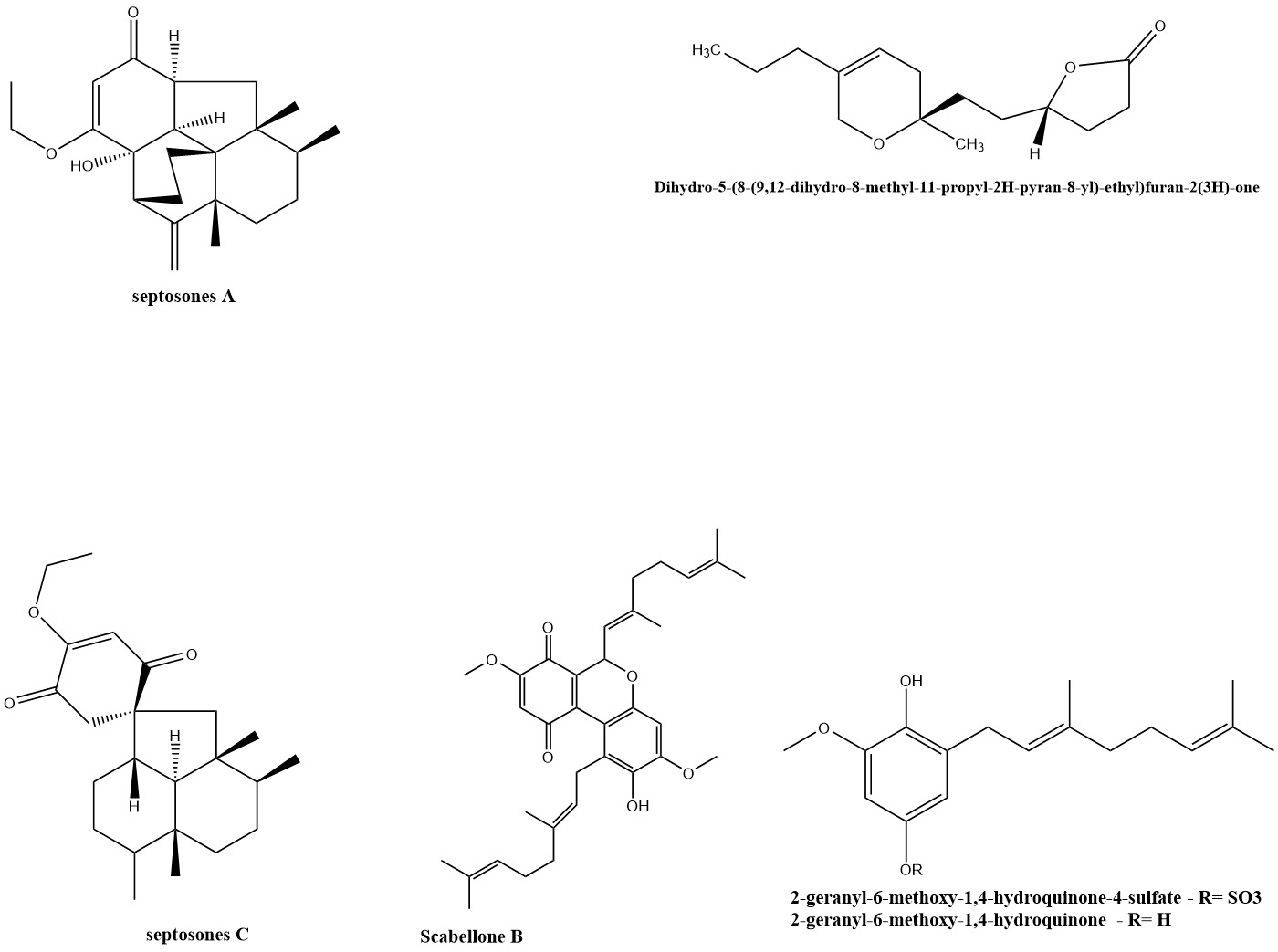

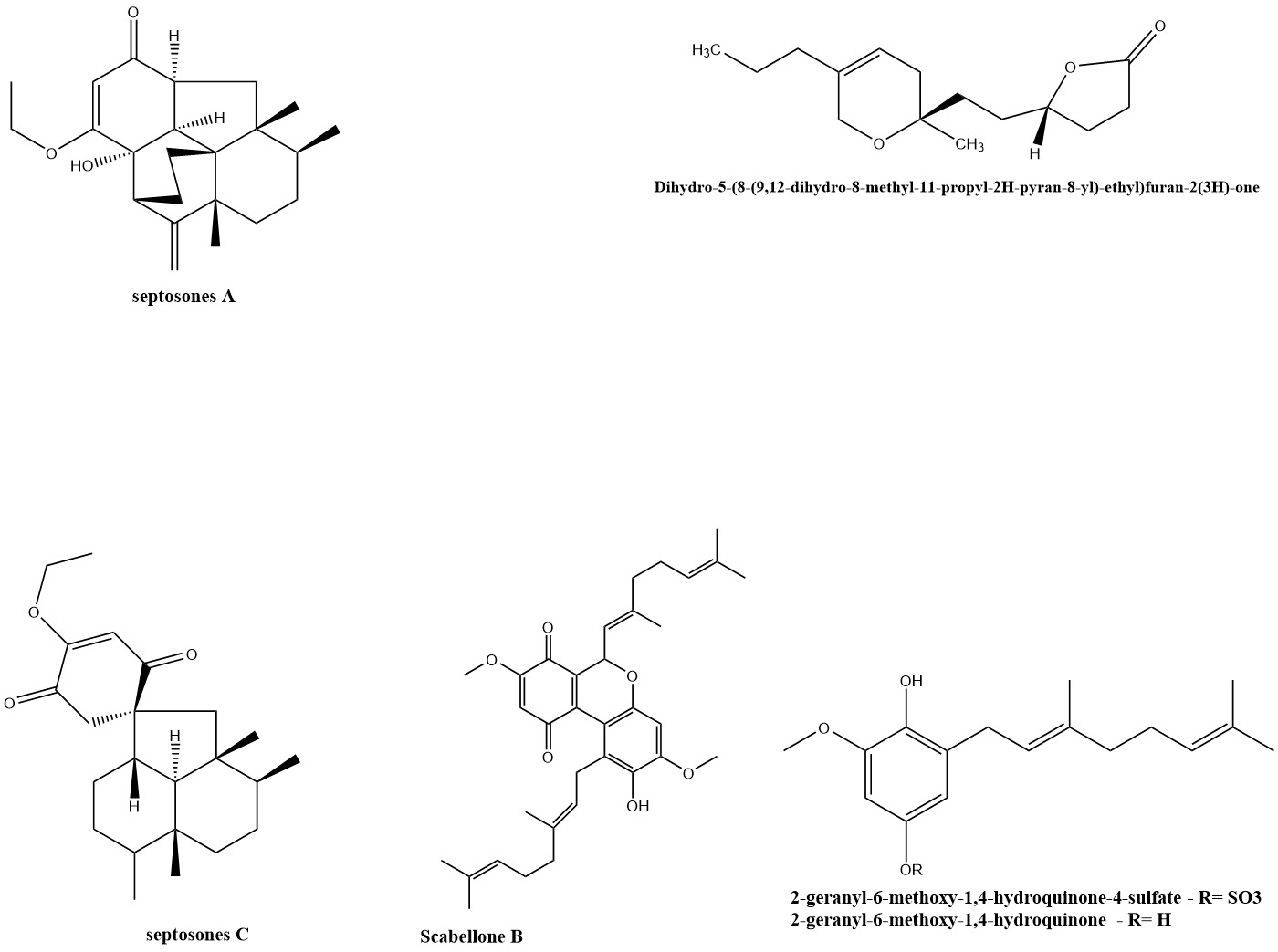


**Fig. No.14: Meroterpenoids showing anti-inflammatory activity**


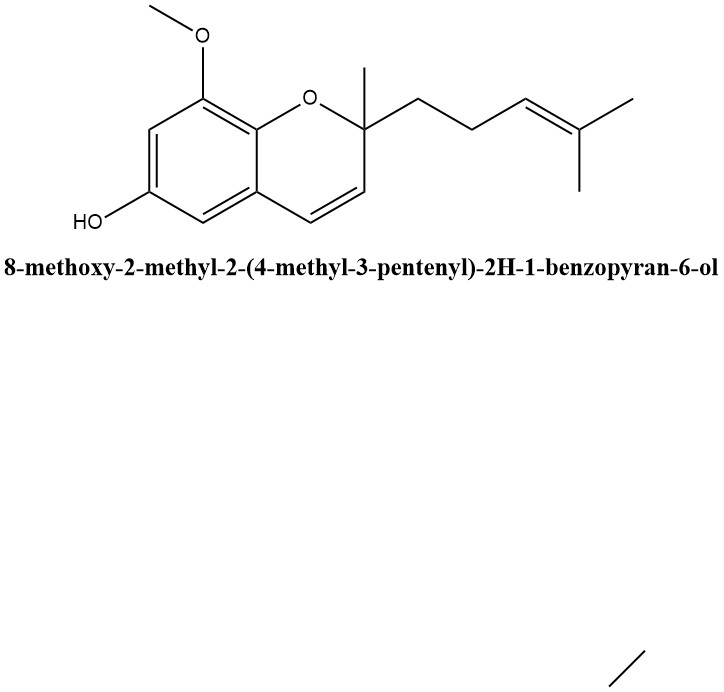

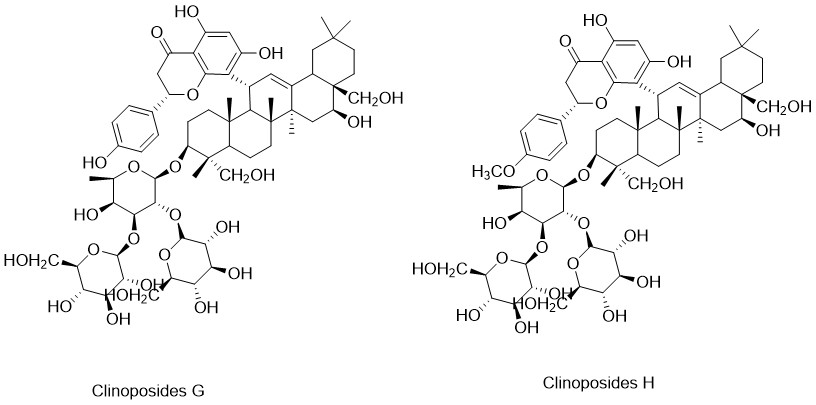

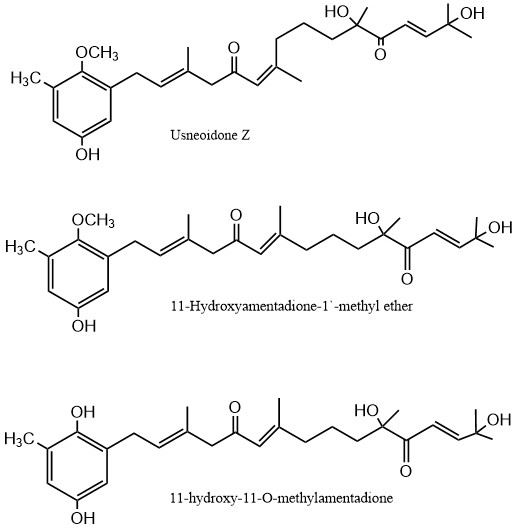

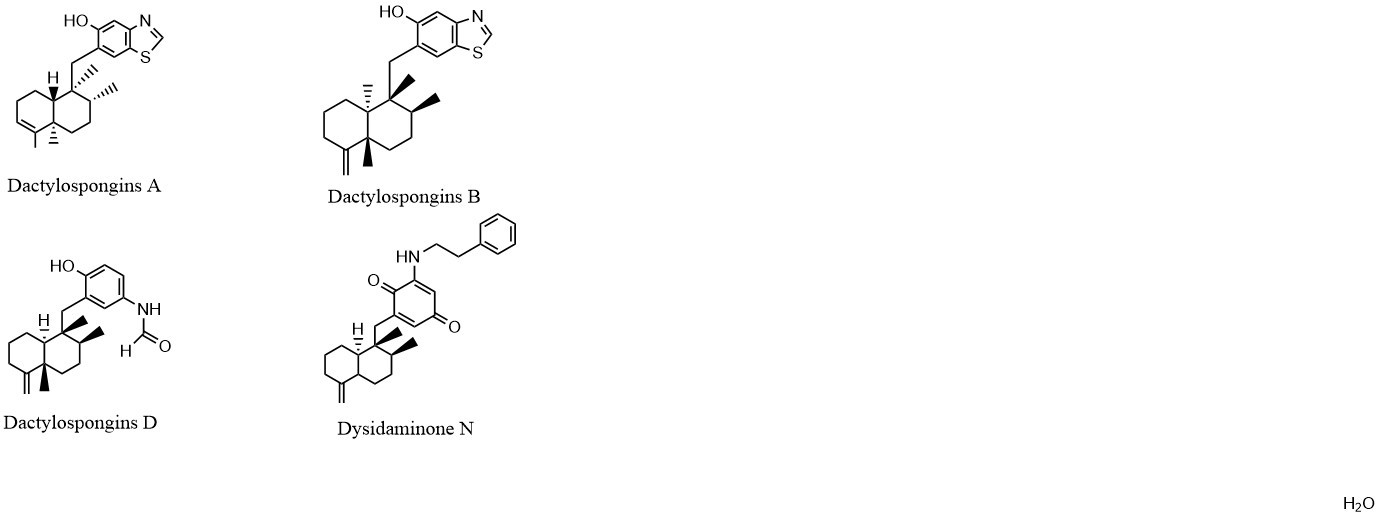


**Fig. No.15: Meroterpenoids showing anti-inflammatory activity**


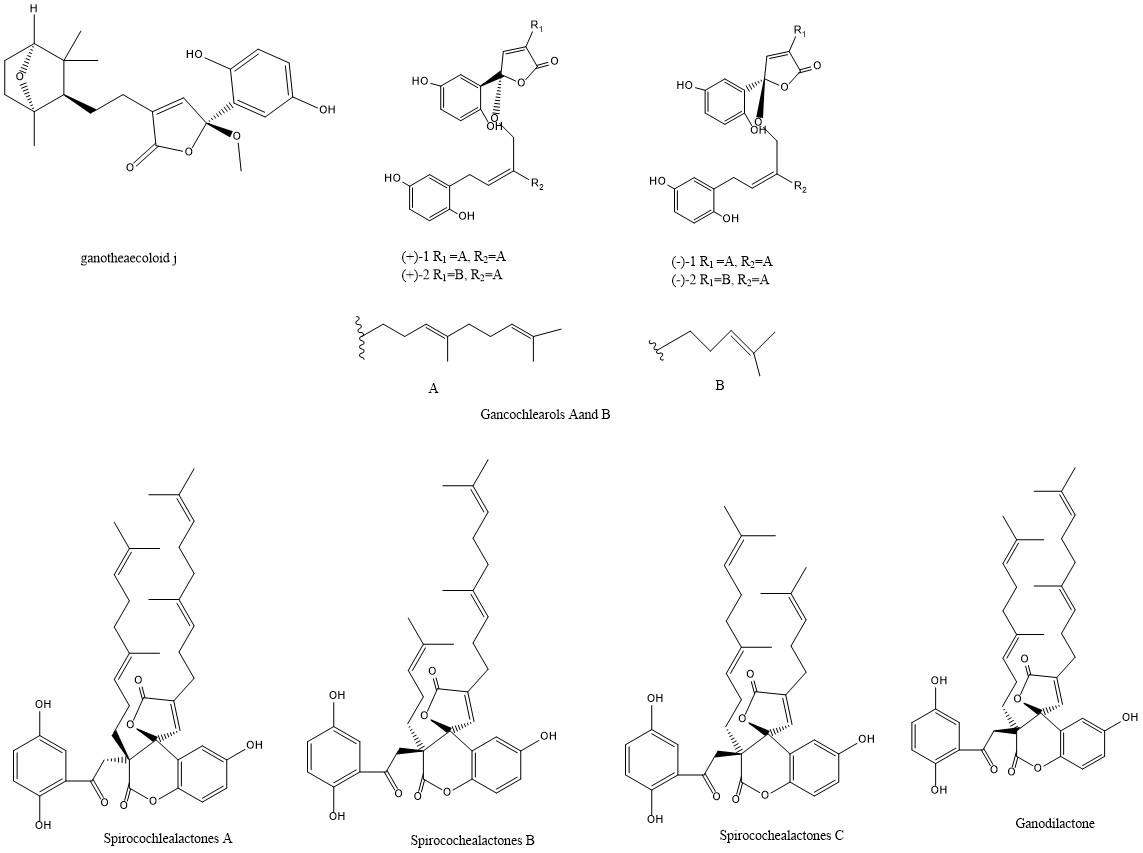


**Fig. No. 16: Meroterpenoids showing COX-2 inhibitory activity**


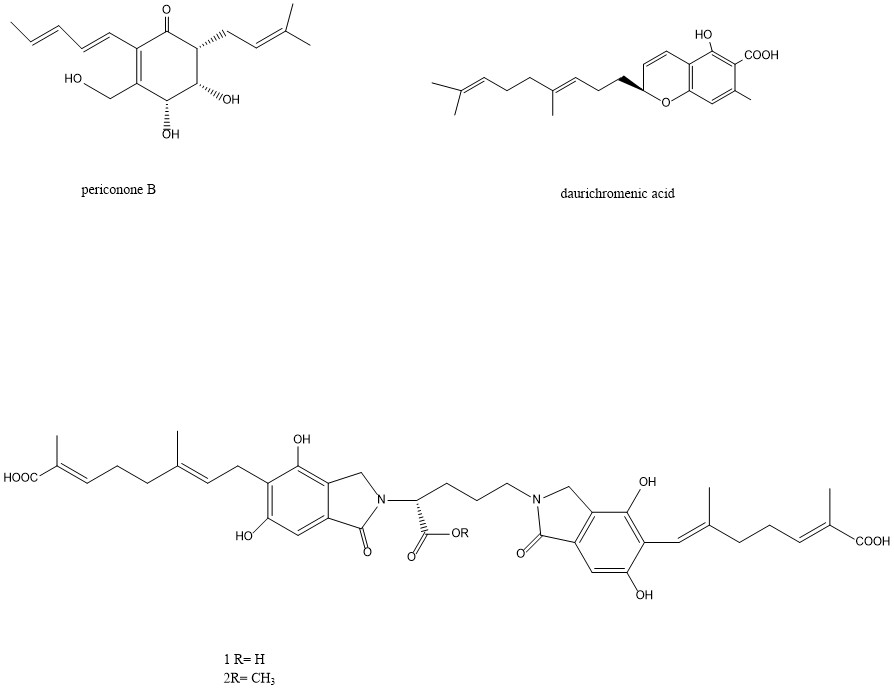


**Fig. No.17: Meroterpenoids showing anti-HIV activity**


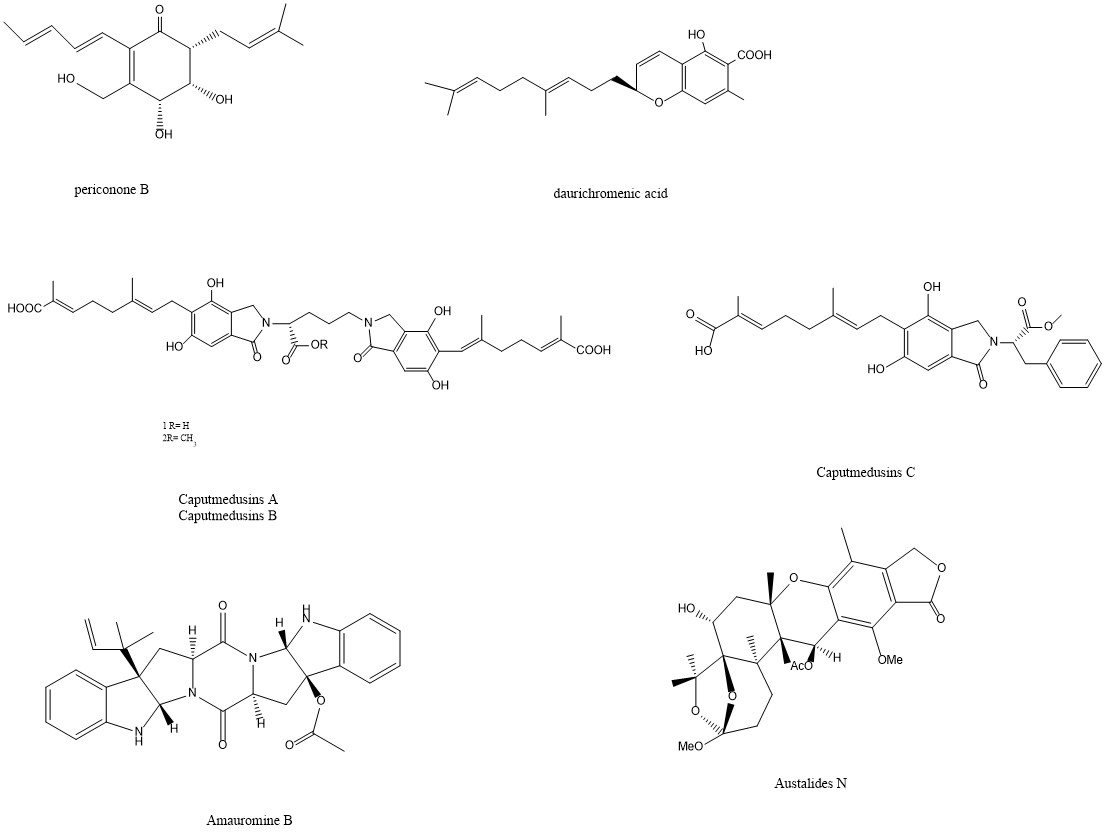

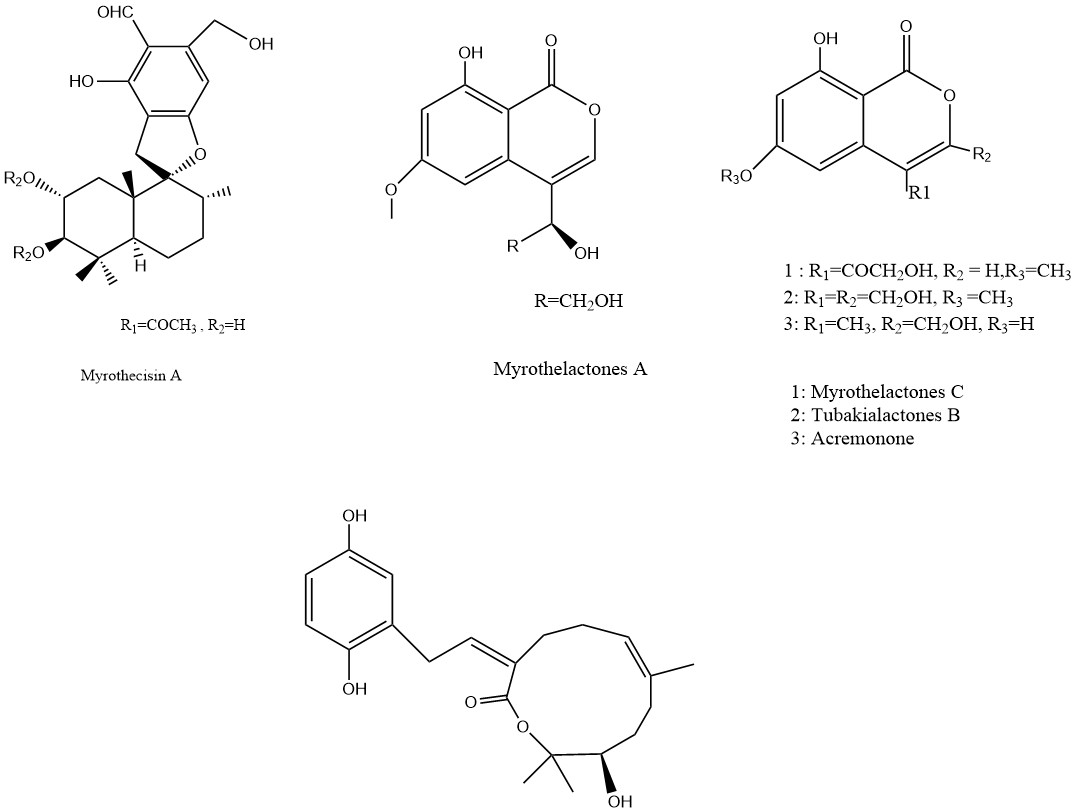


**Fig. No.18: Meroterpenoids showing alpha-glucosidase activity**


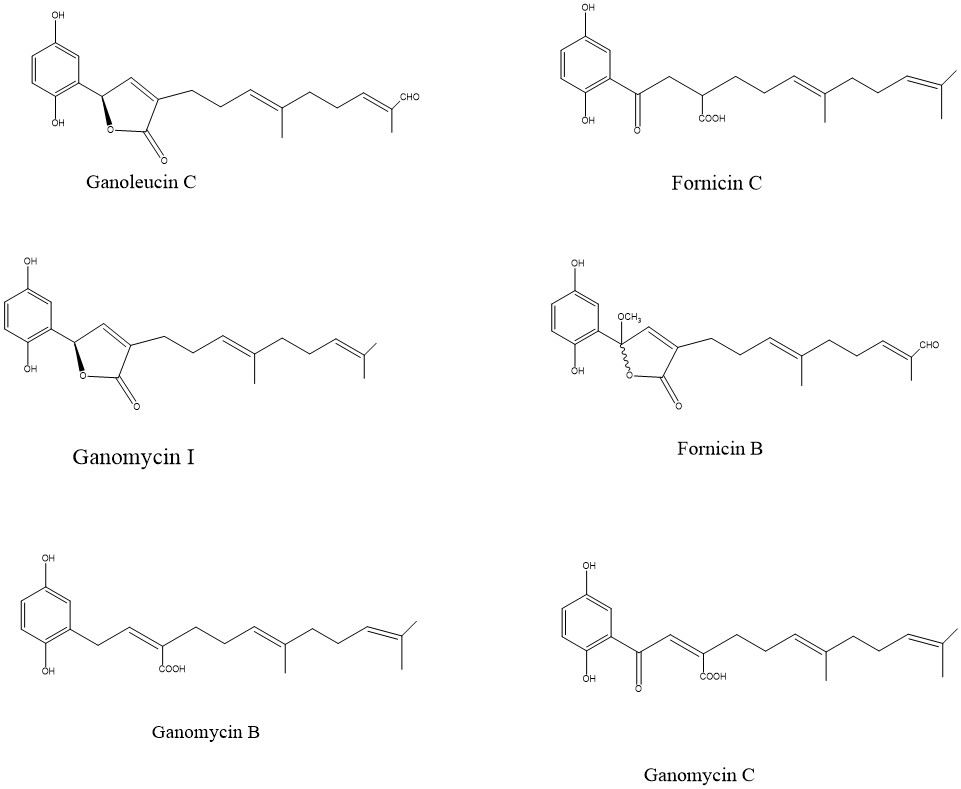

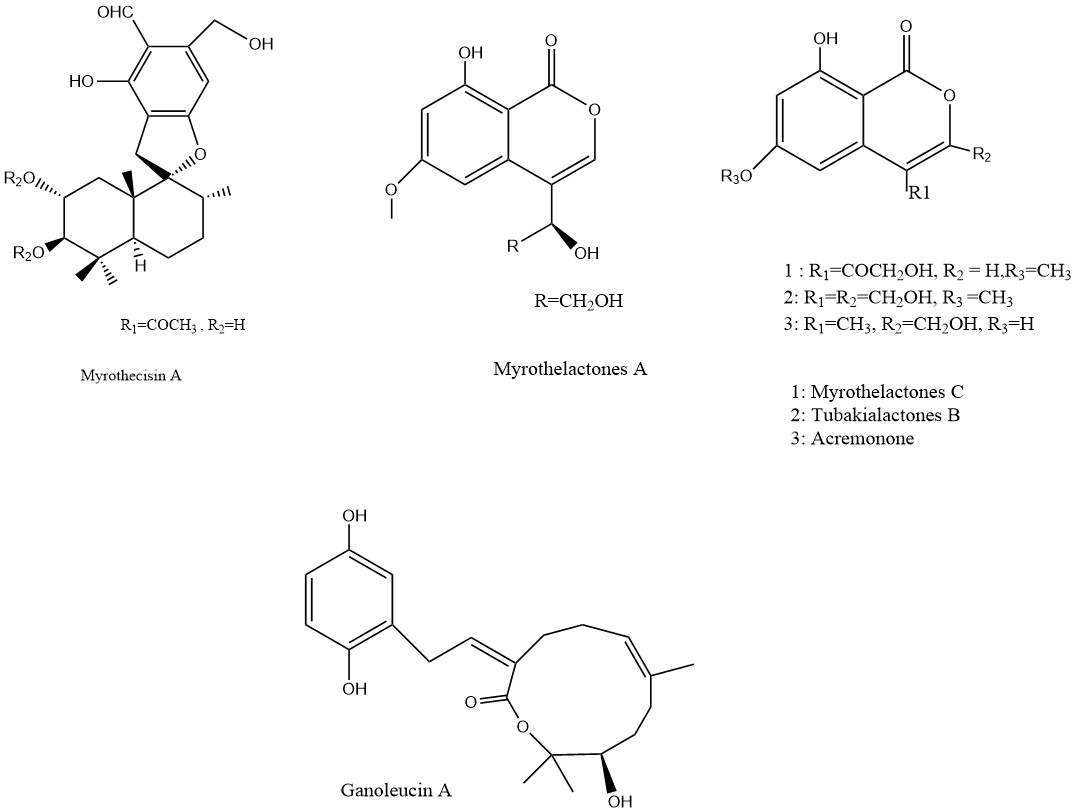


**Fig. No.19: Meroterpenoids showing alpha-glucosidase activity**


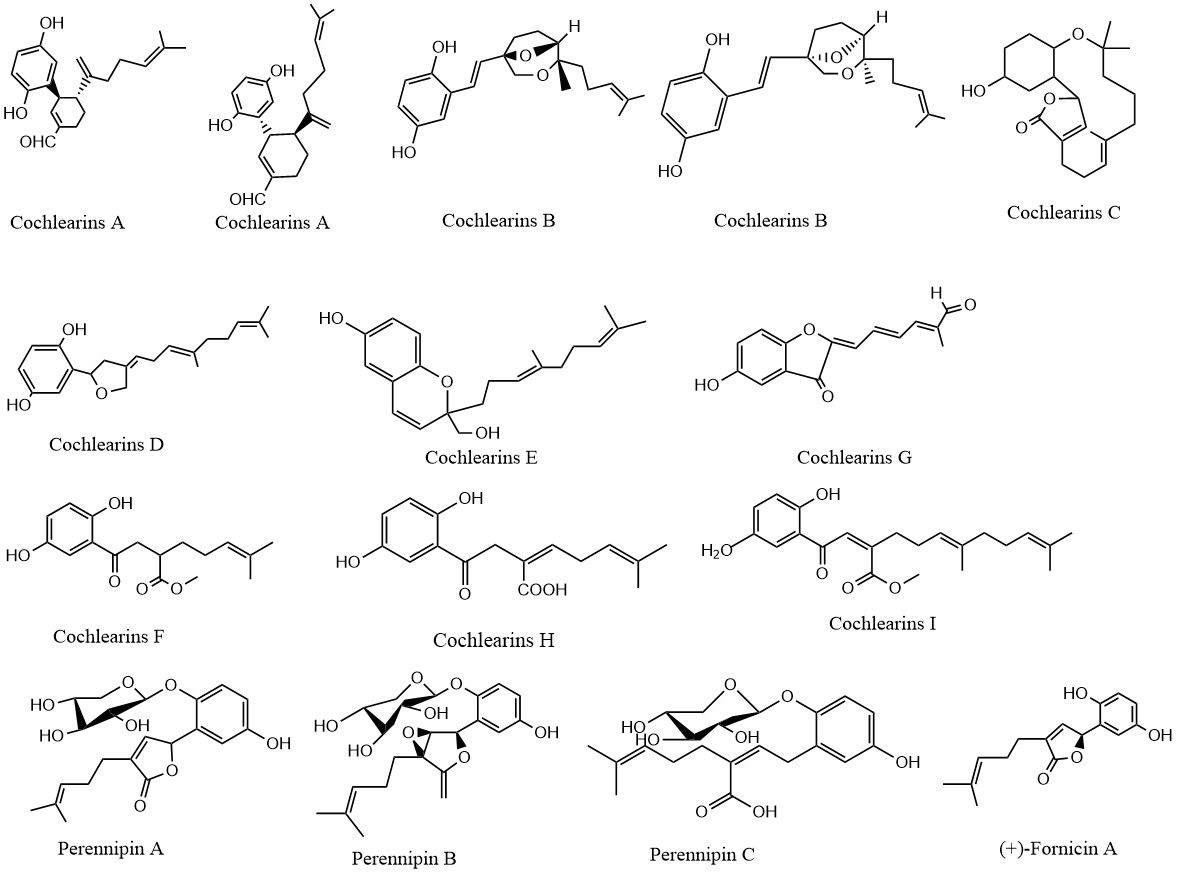


**Fig. No. 20: Meroterpenoids showing antioxidant activity**


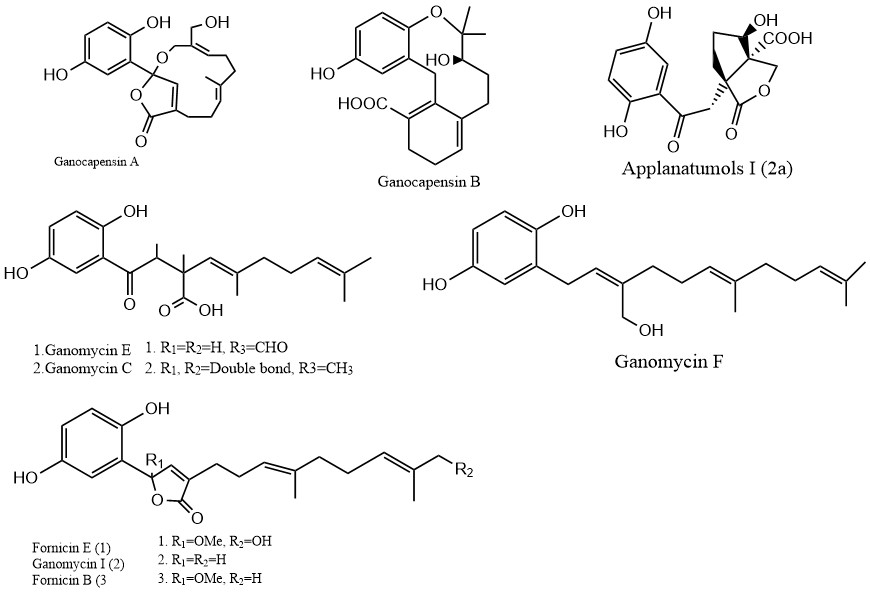

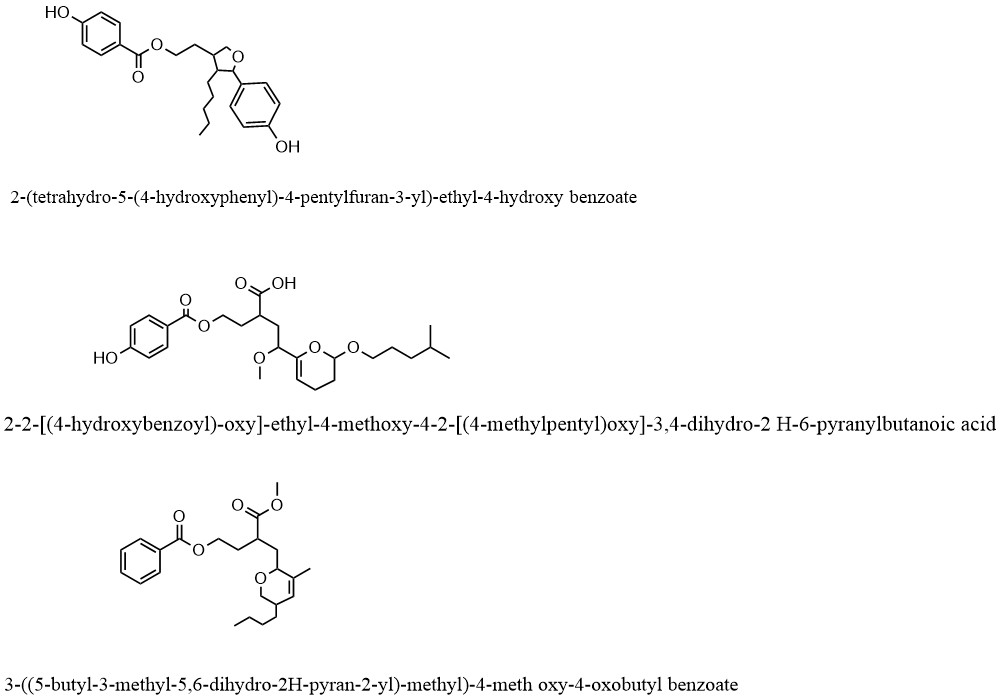


**Fig. No. 21: Meroterpenoids showing antioxidant activity**


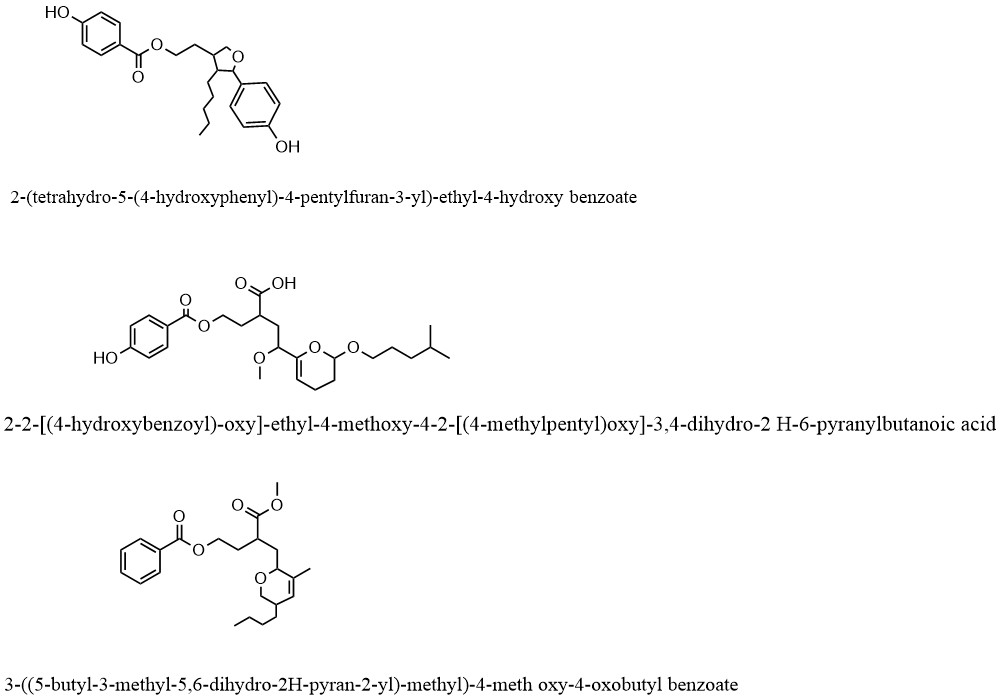

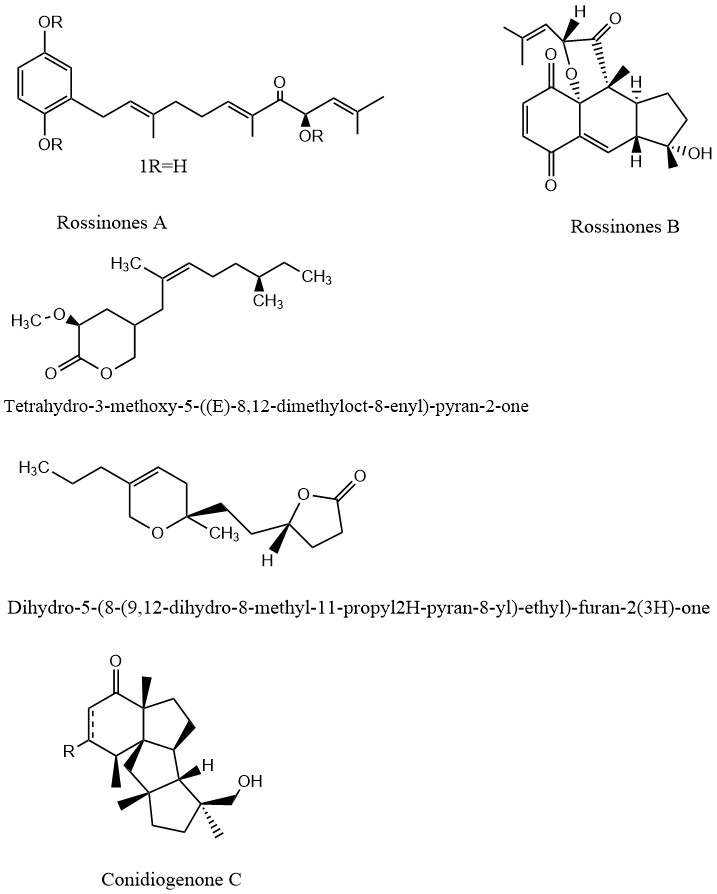


**Fig. No. 22: Meroterpenoids showing antioxidant activity**


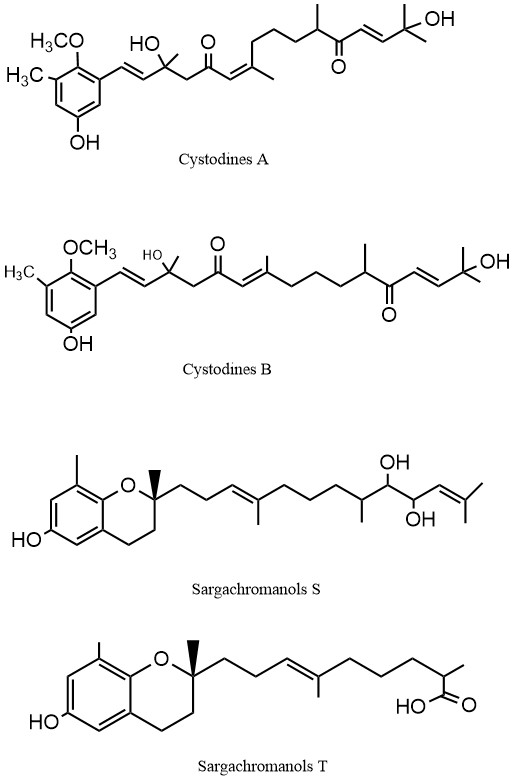

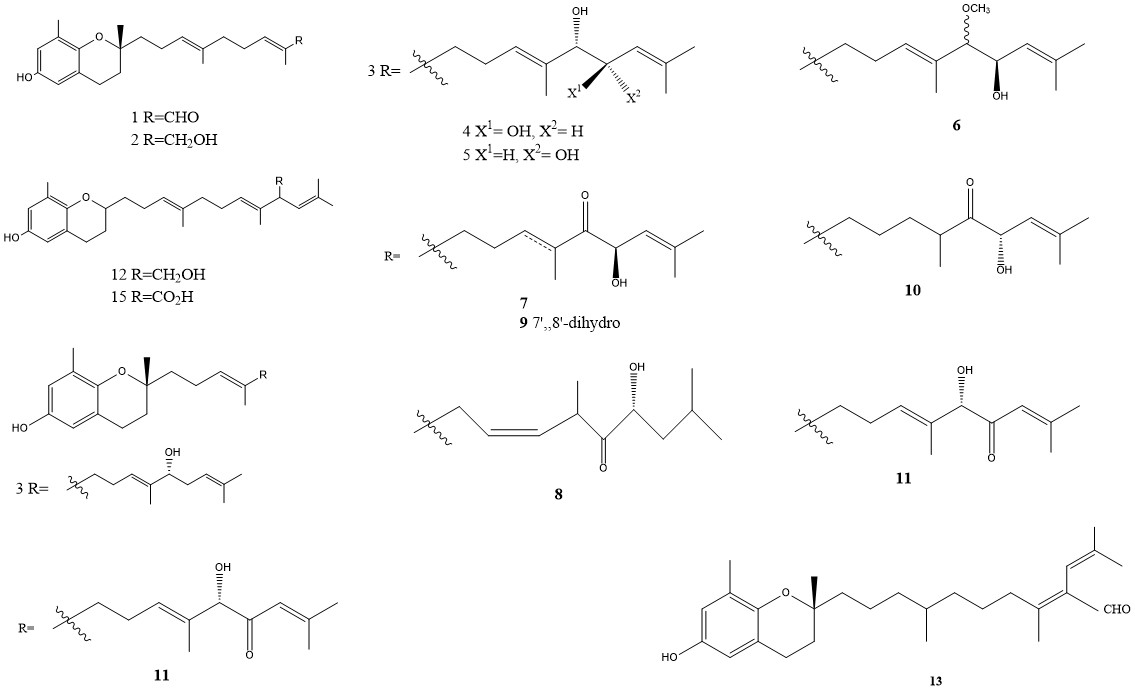


**Fig. No. 23: Meroterpenoids showing antioxidant activity**


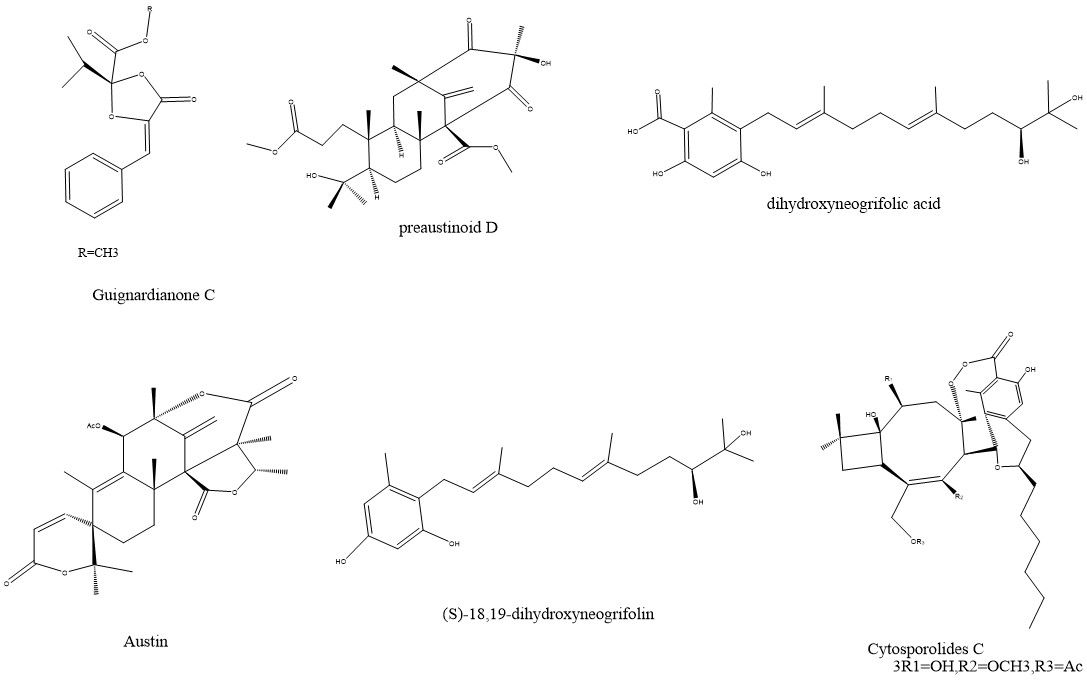

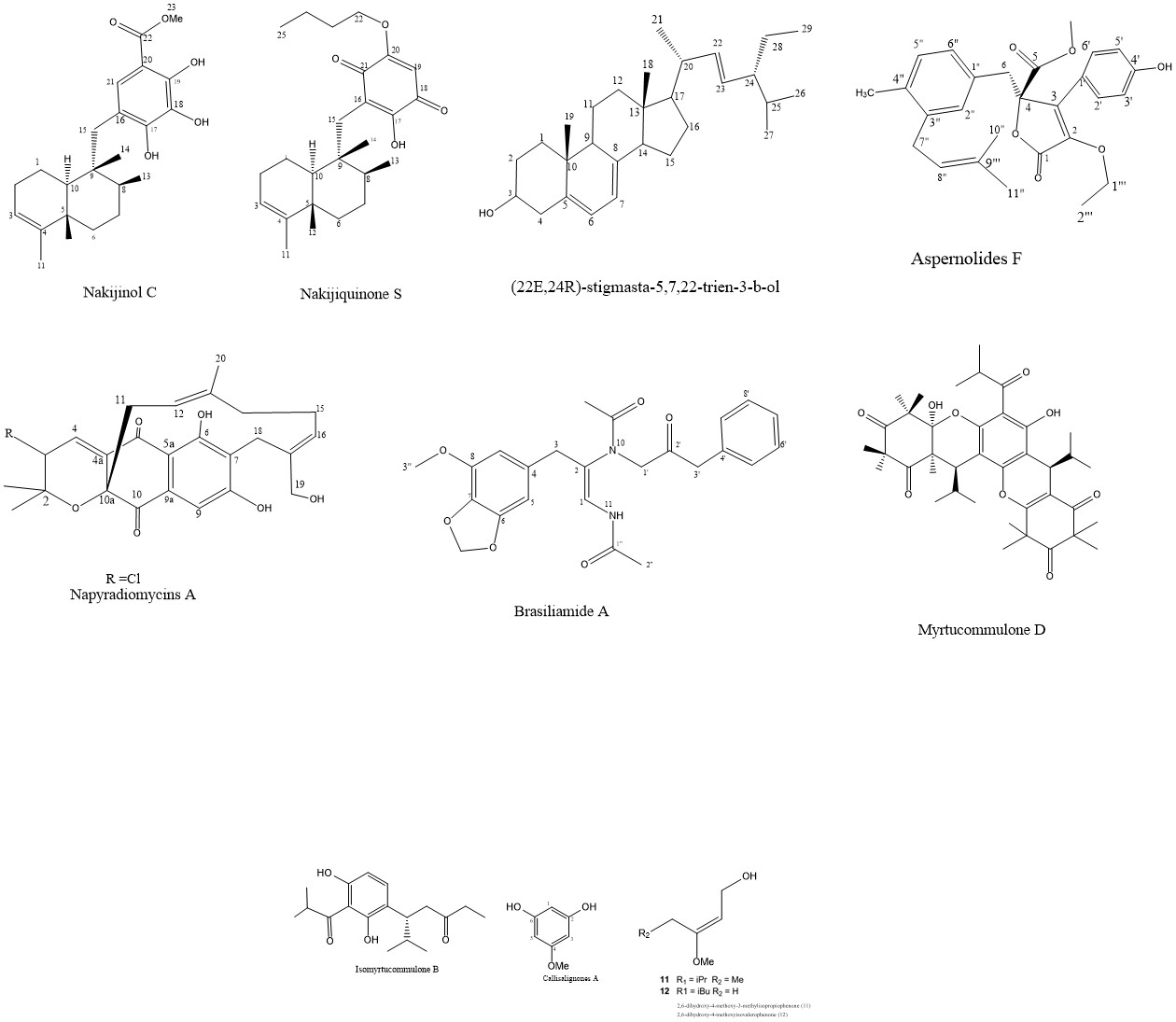

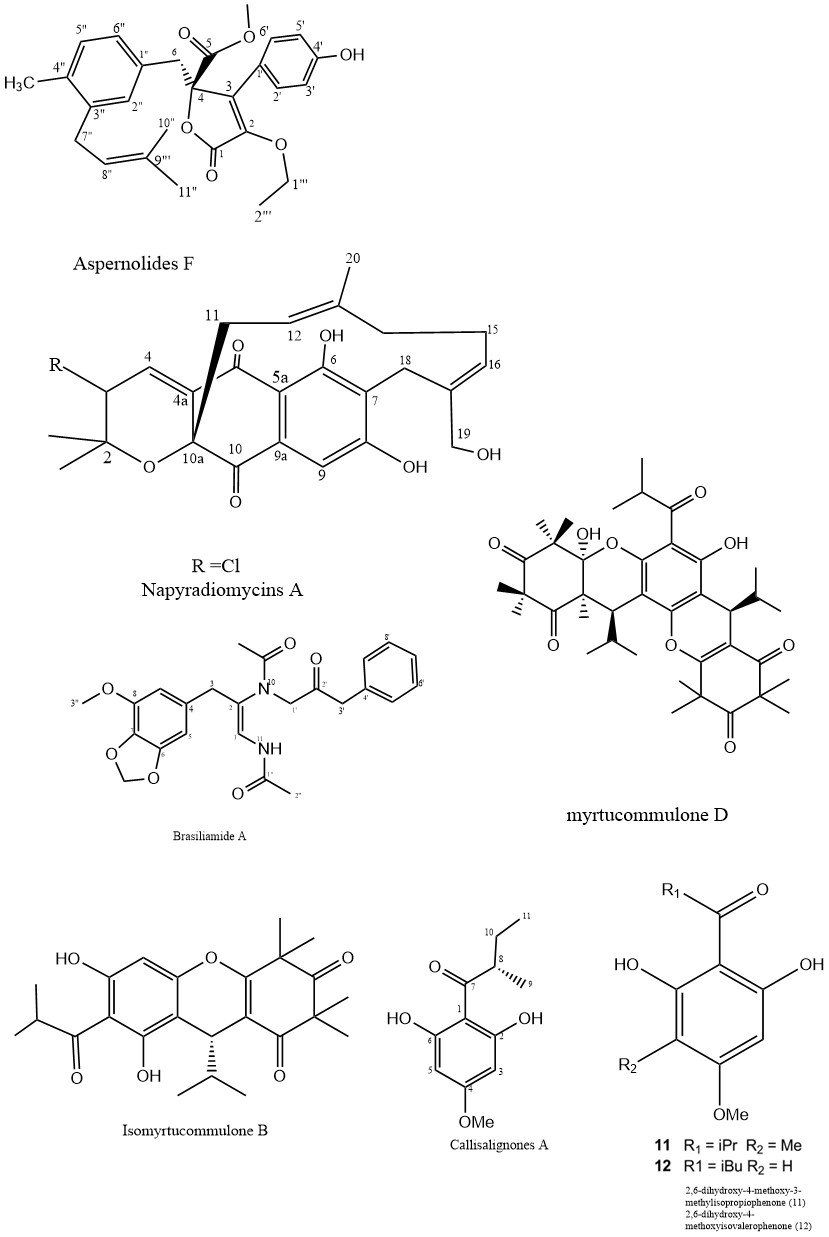


**Fig. No. 24: Meroterpenoids showing antimicrobial activity**


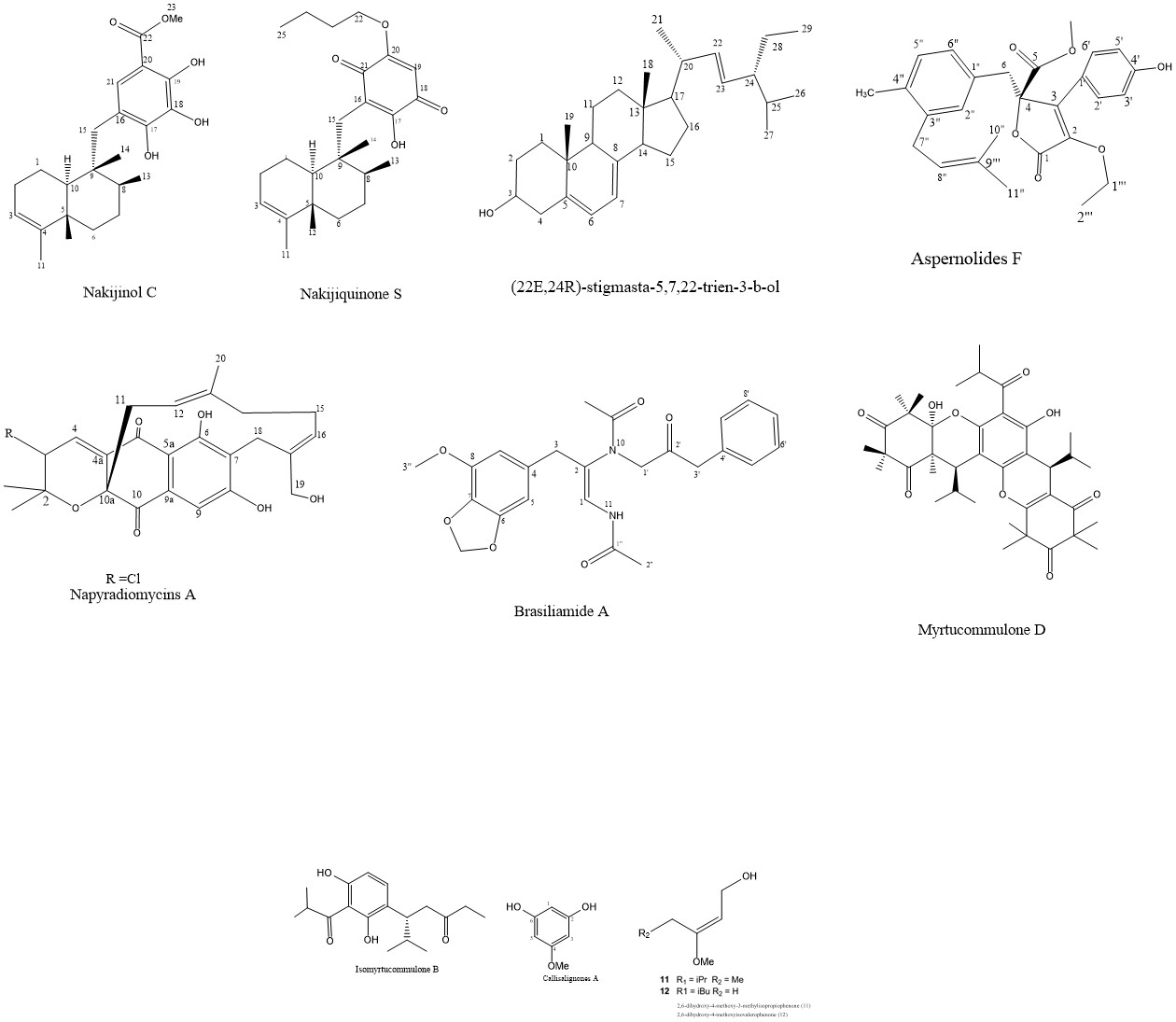

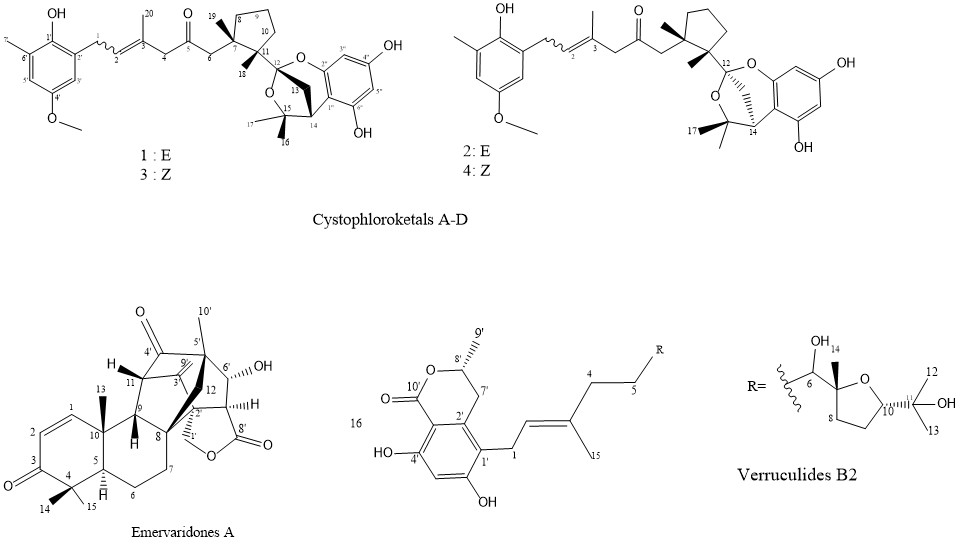

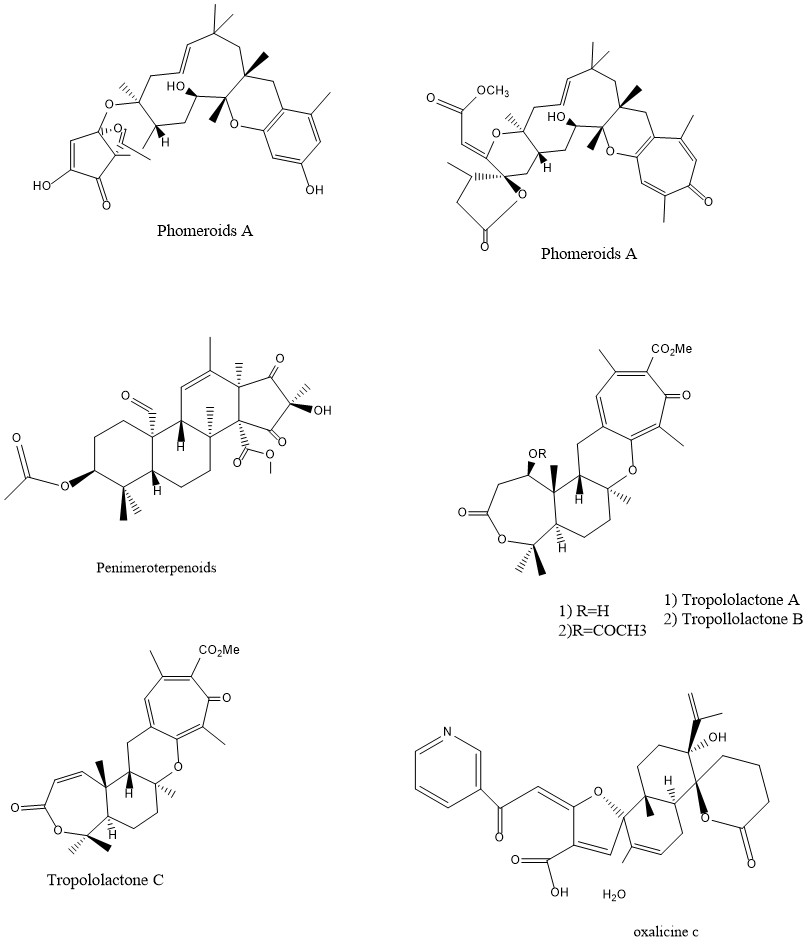


**Fig. No. 25: Meroterpenoids showing antimicrobial activity**


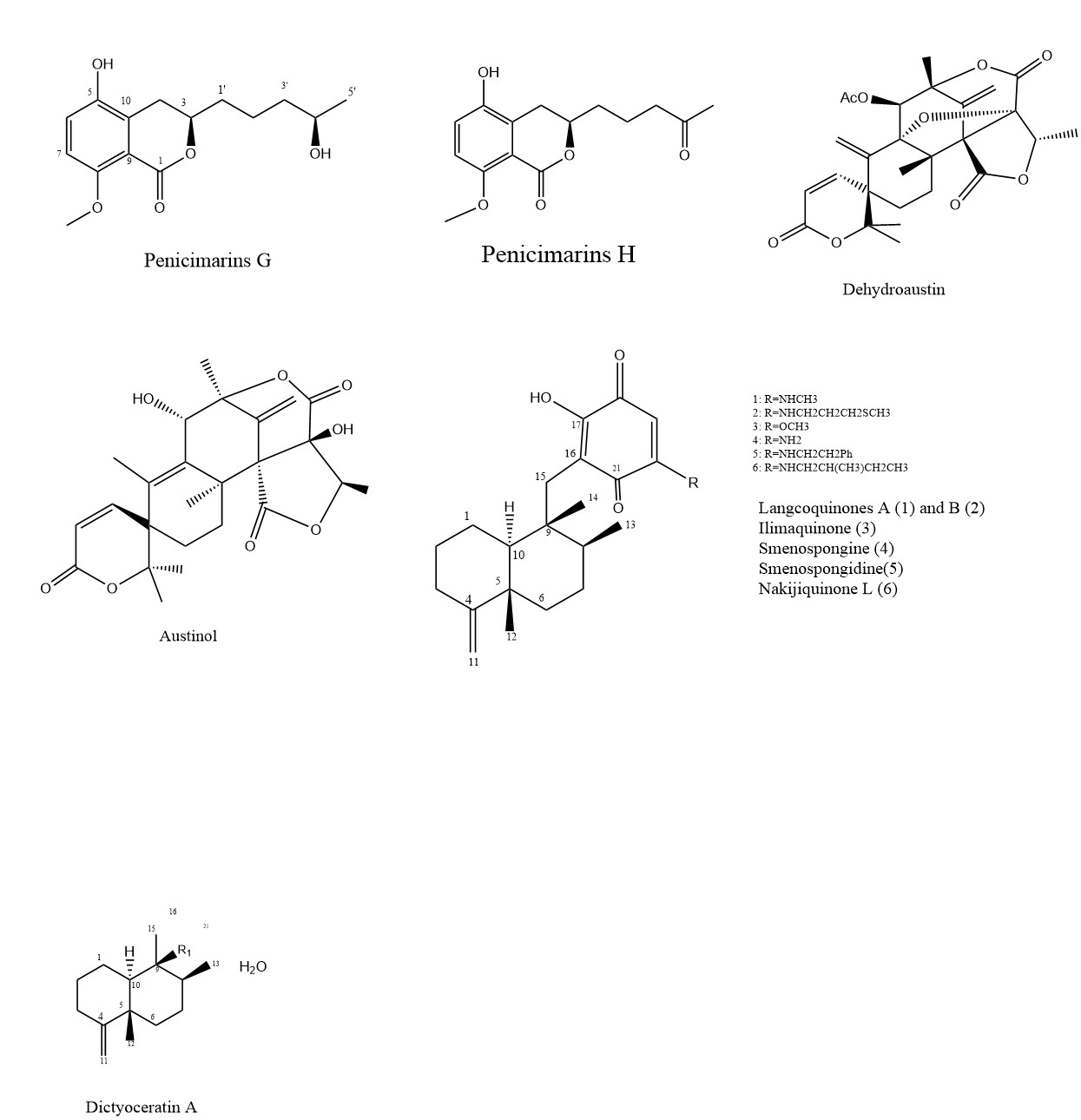

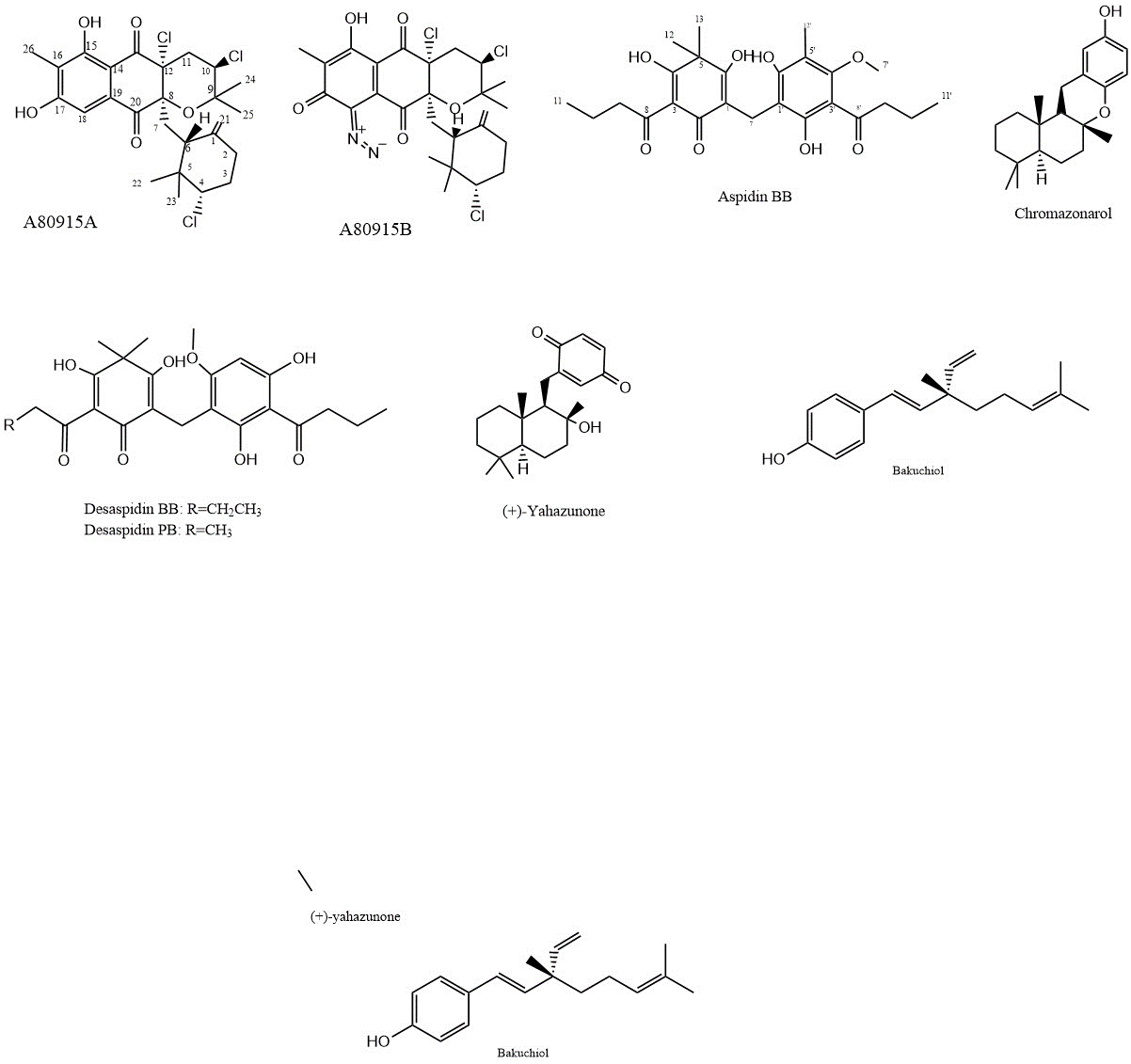


**Fig No. 26: Meroterpenoids showing antibacterial activity**


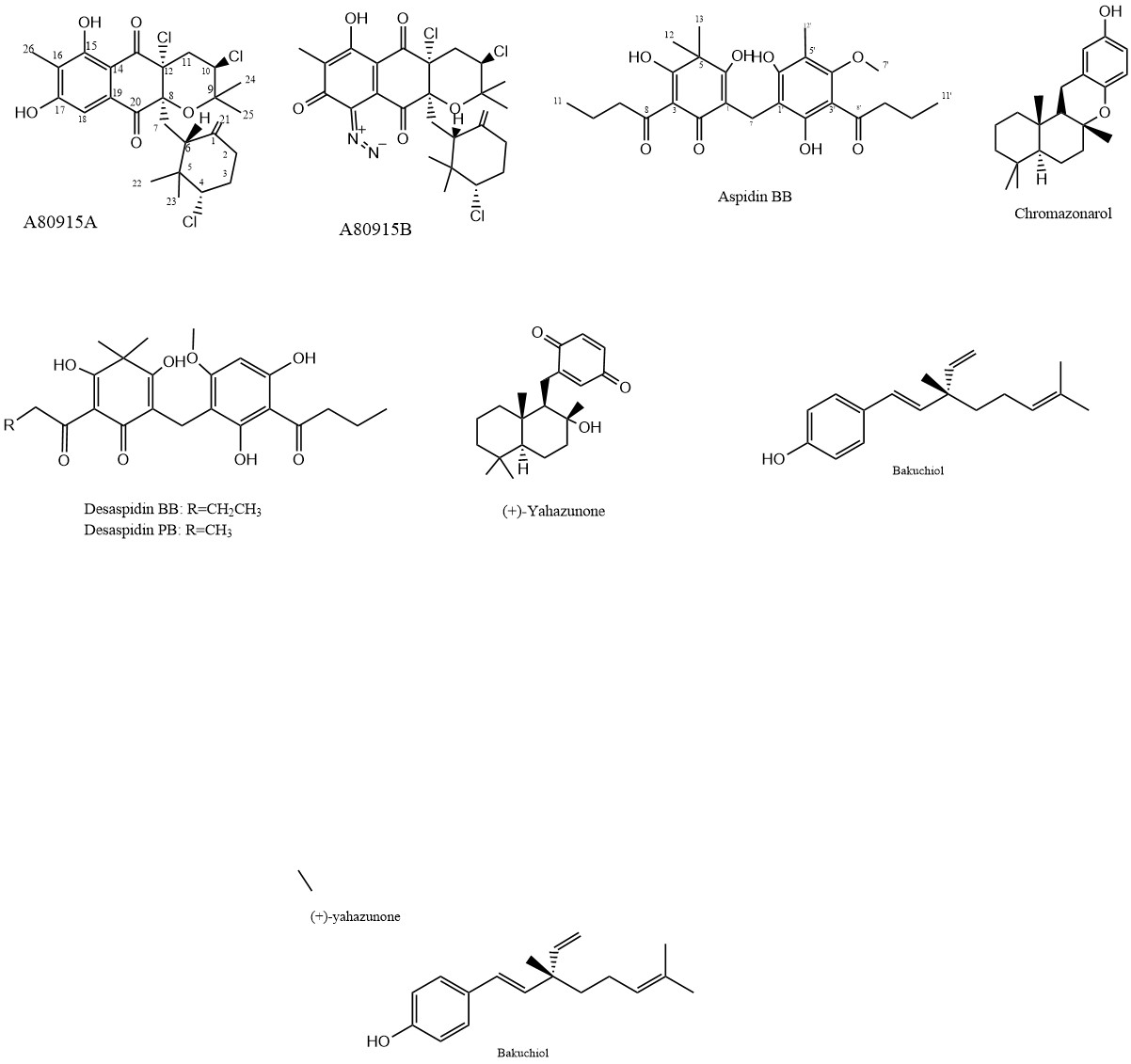

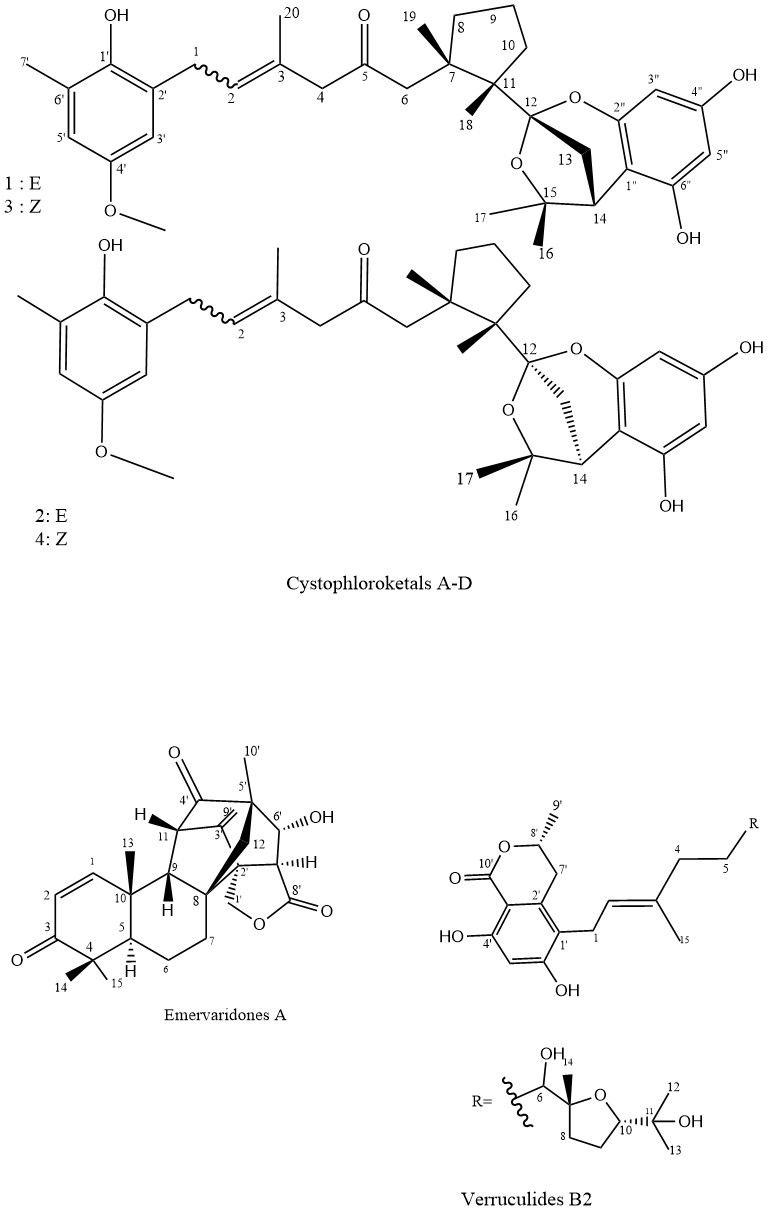

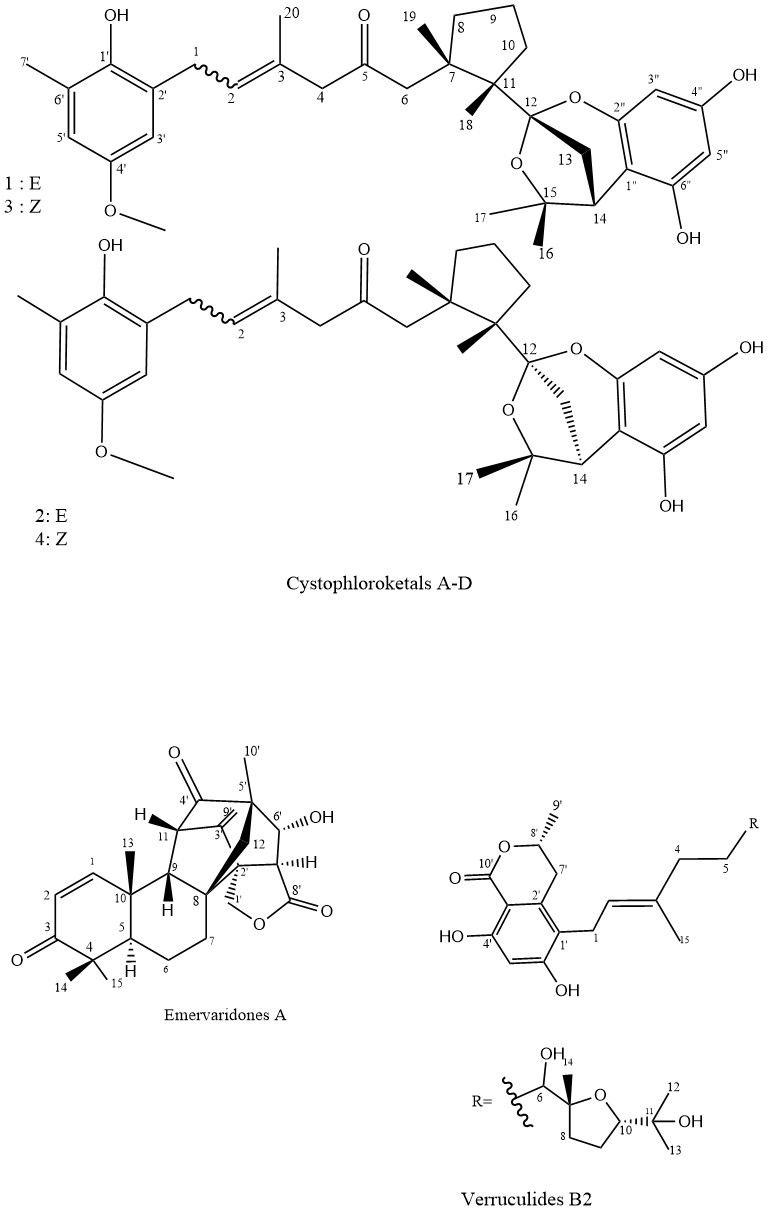


**Fig No. 27: Meroterpenoids showing antibacterial activity**


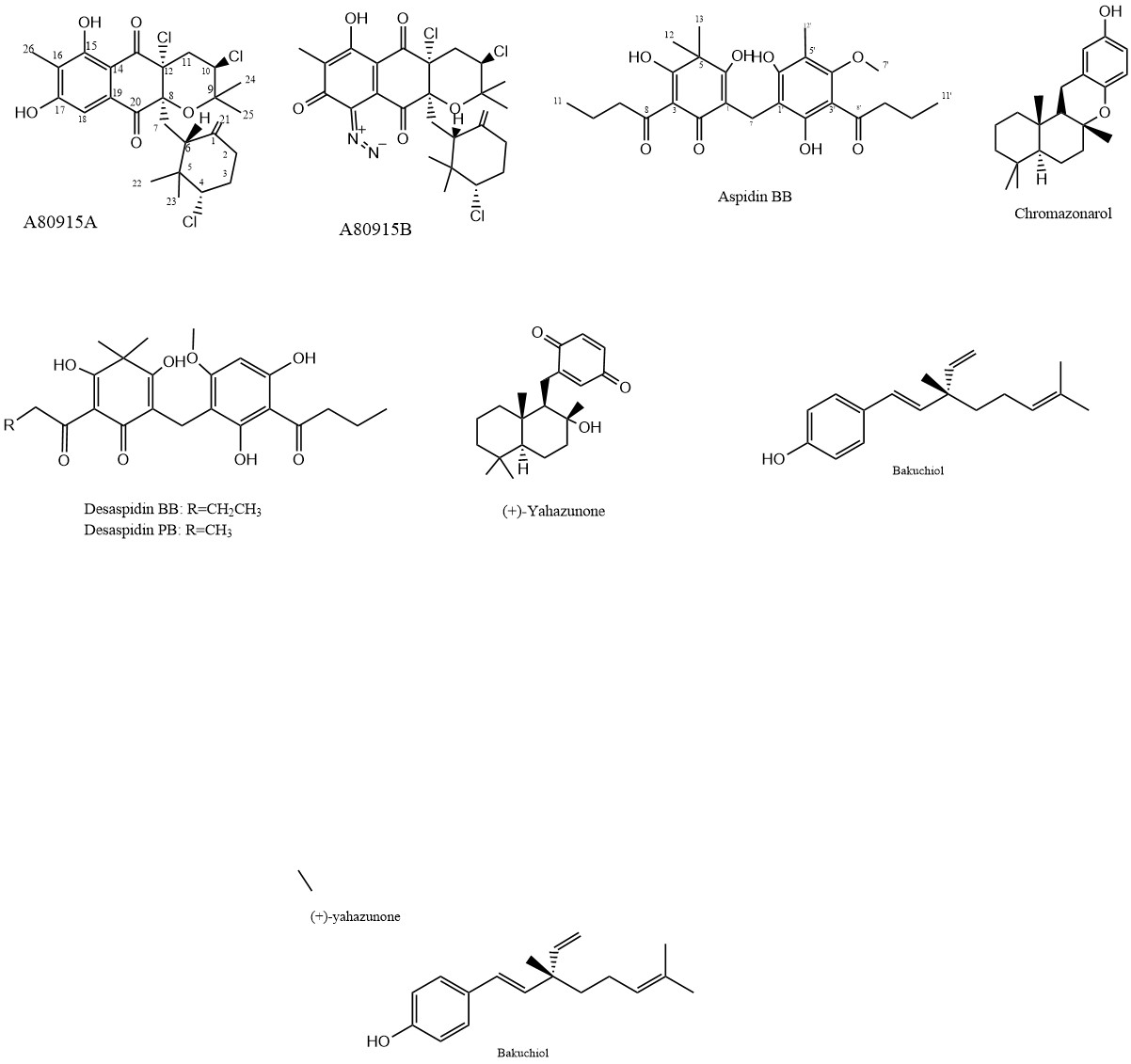

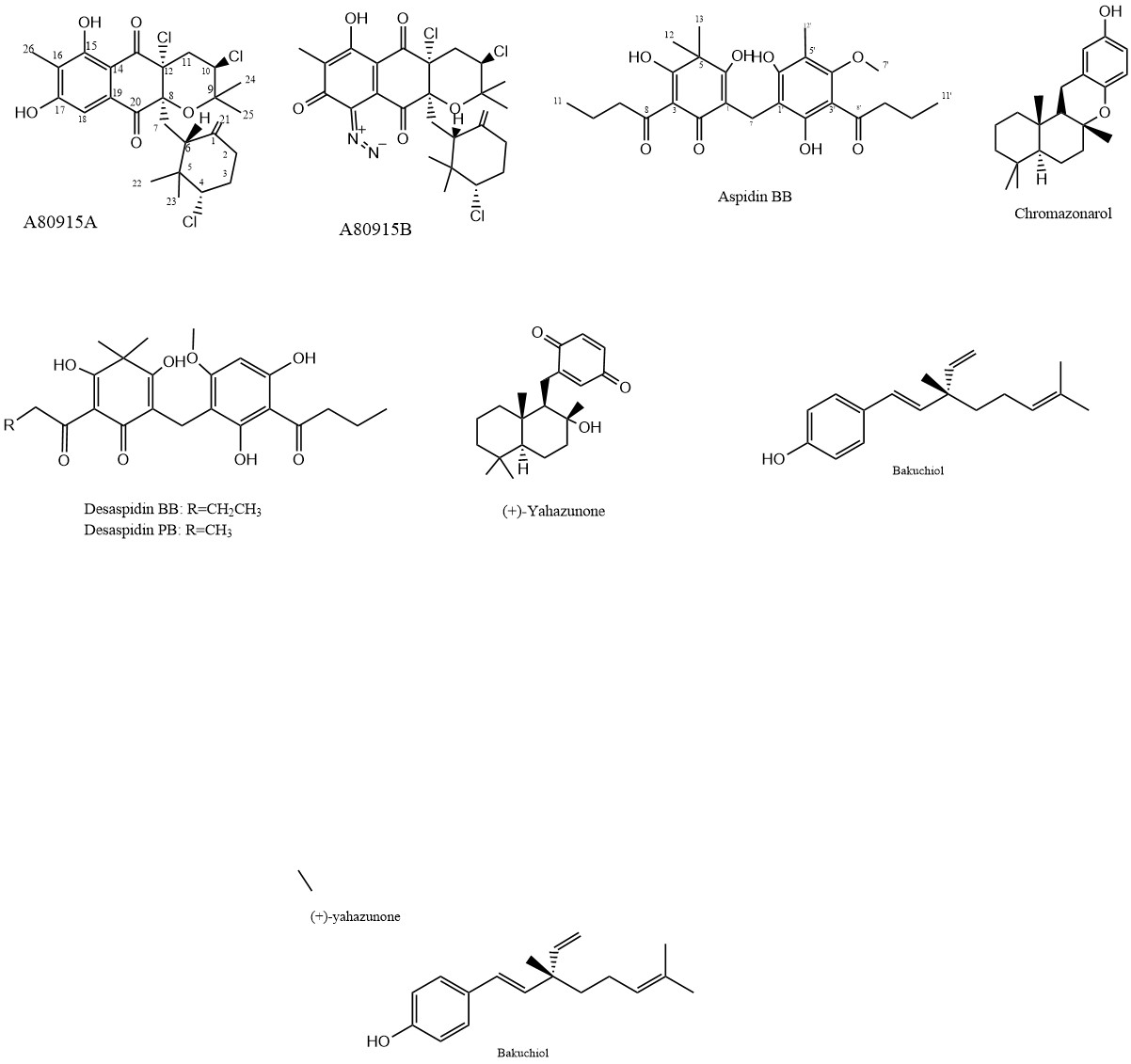

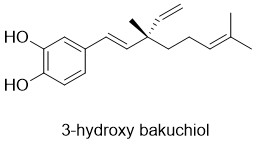


**Fig. No. 28: Meroterpenoids showing antifungal activity**


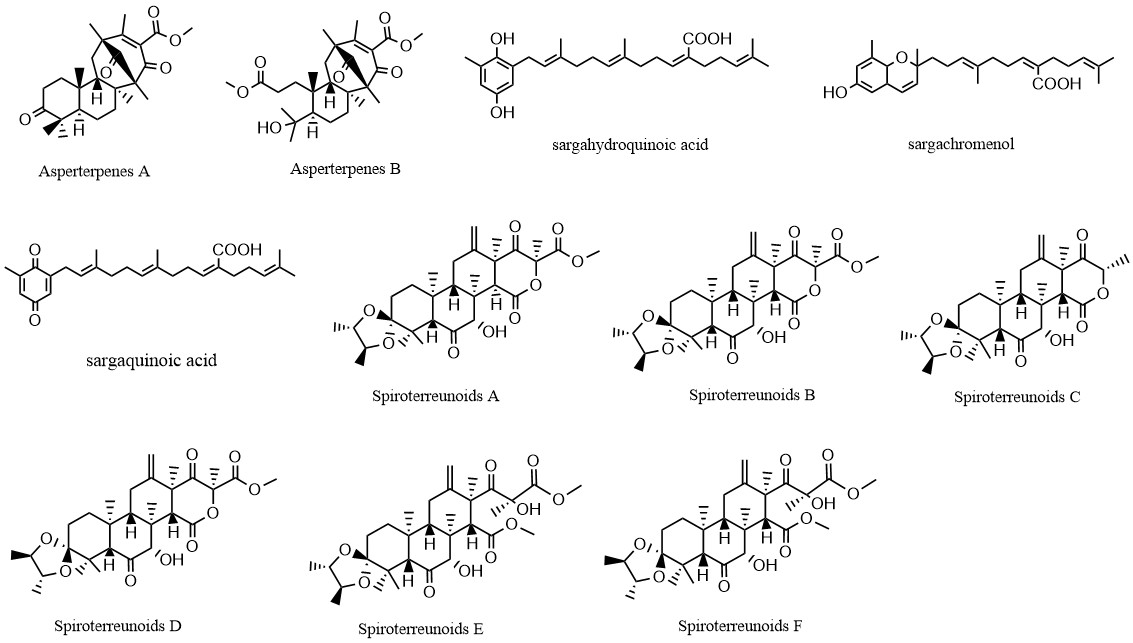

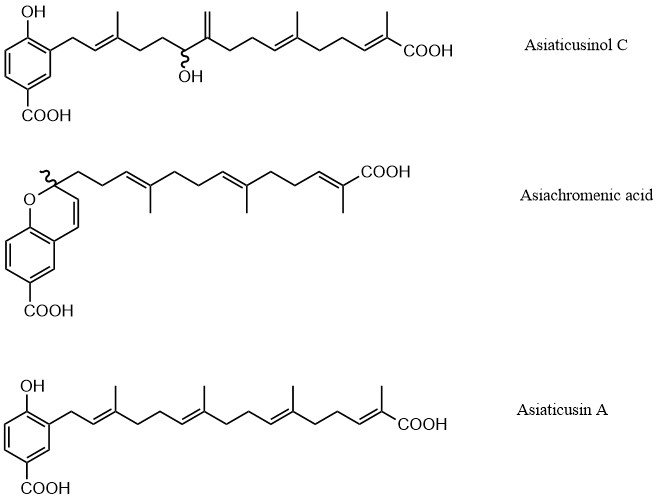


**Fig. No. 29: Meroterpenoids showing BACE1 inhibitory activity**


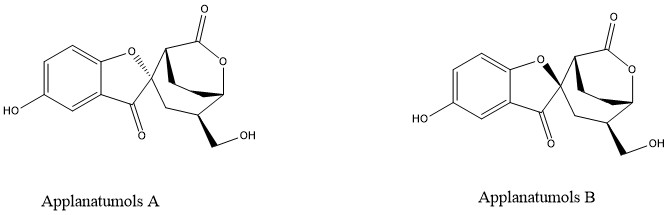


**Fig. No. 30: Meroterpenoids showing renal protective activity**

**
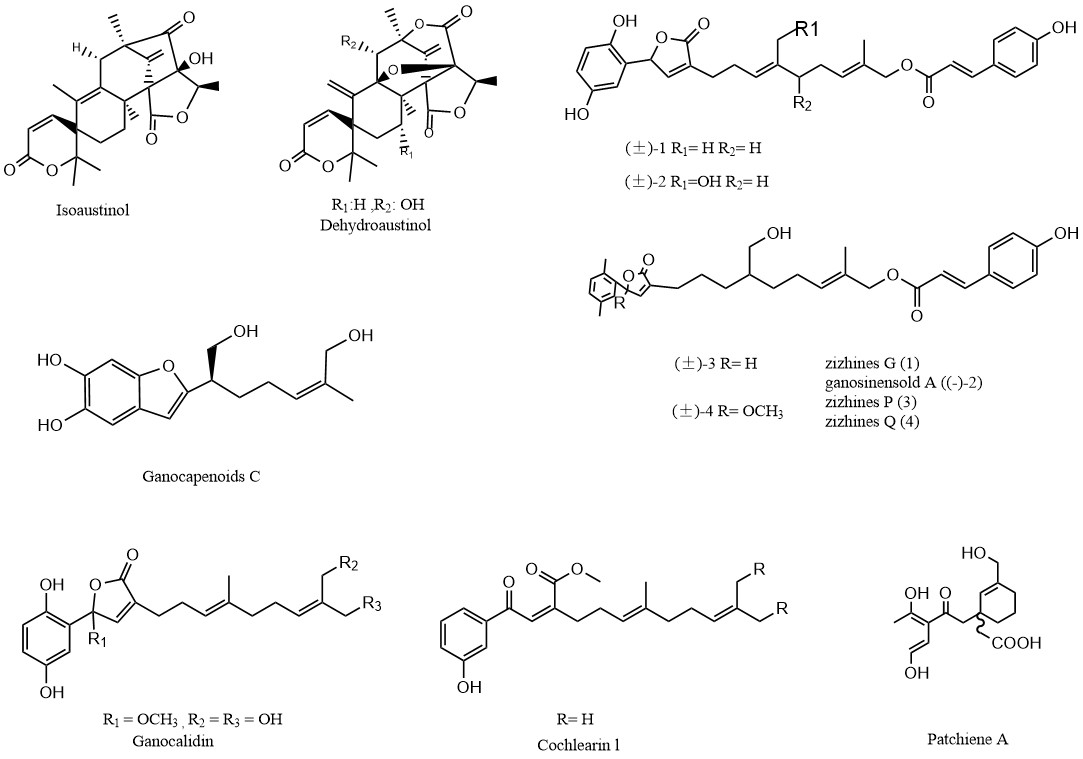
**

**Fig. No. 31: AchE inhibitory activity of Meroterpenoids**


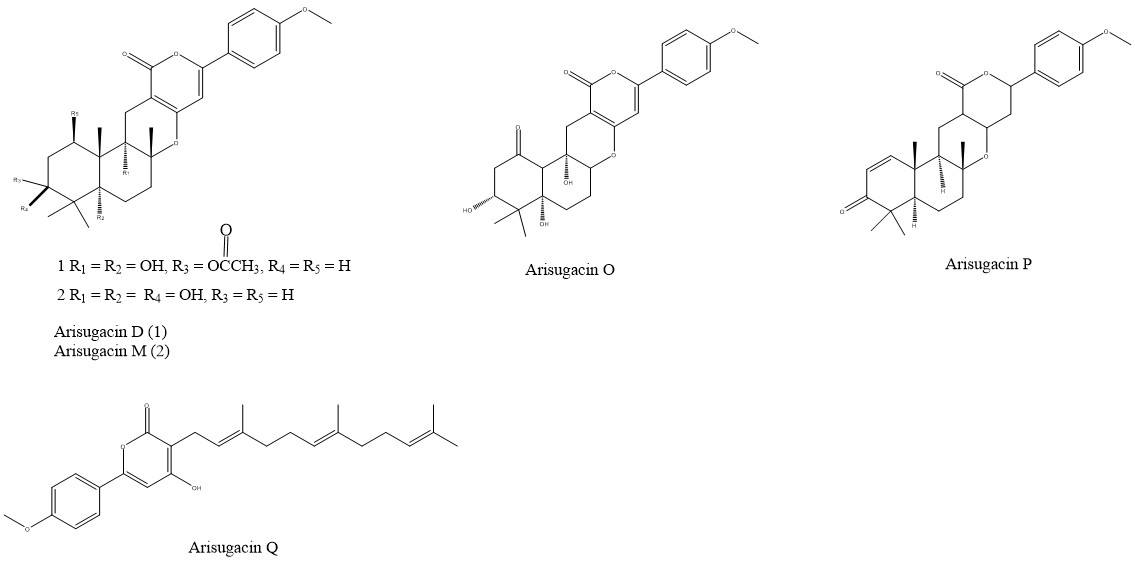

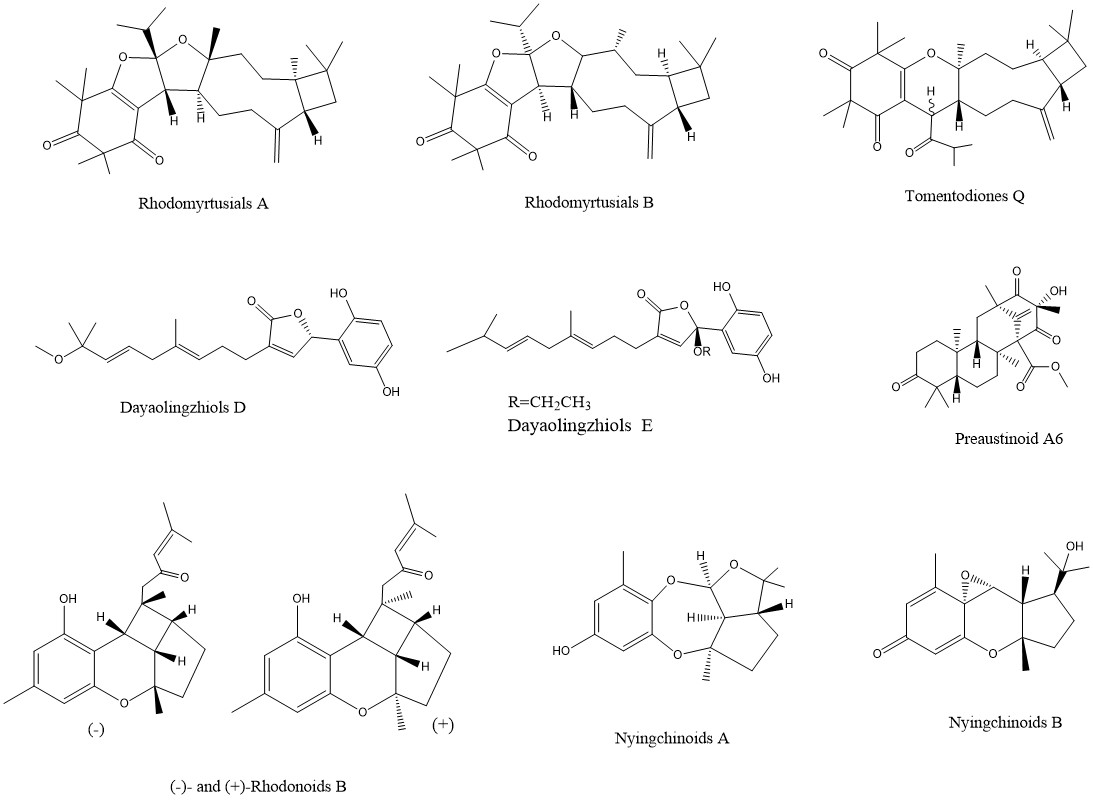

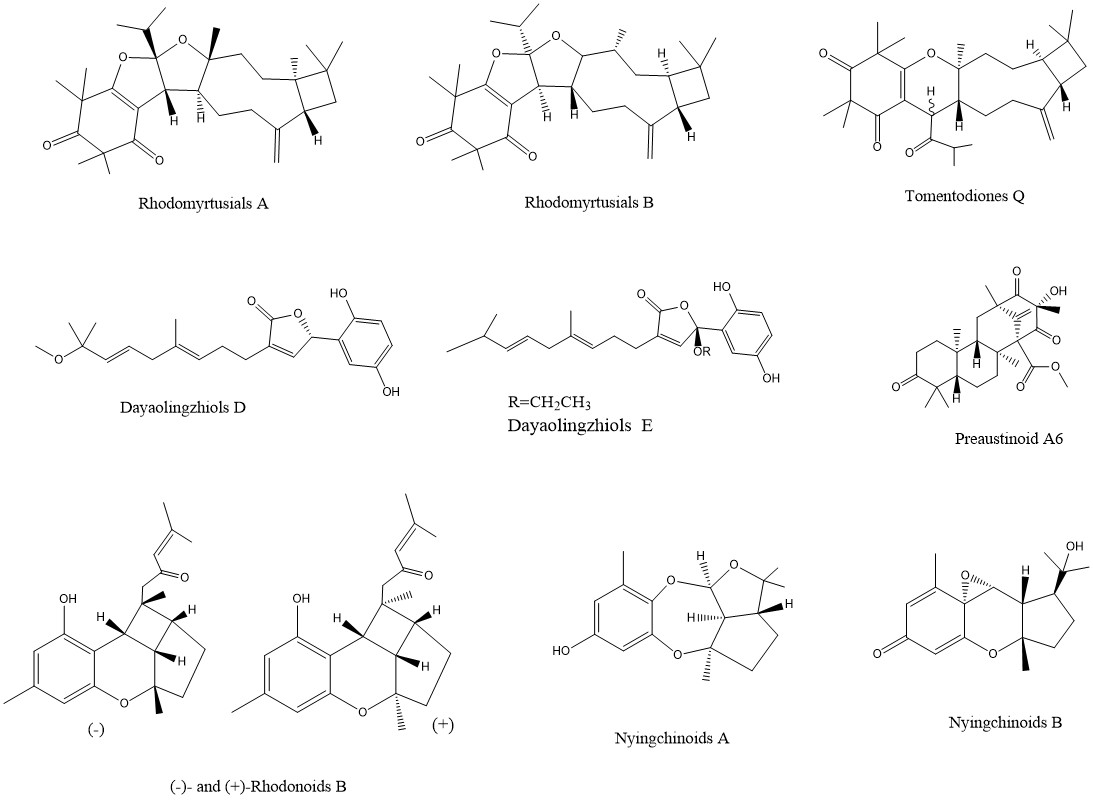


**Fig. No. 32: AchE inhibitory activity of Meroterpenoids**


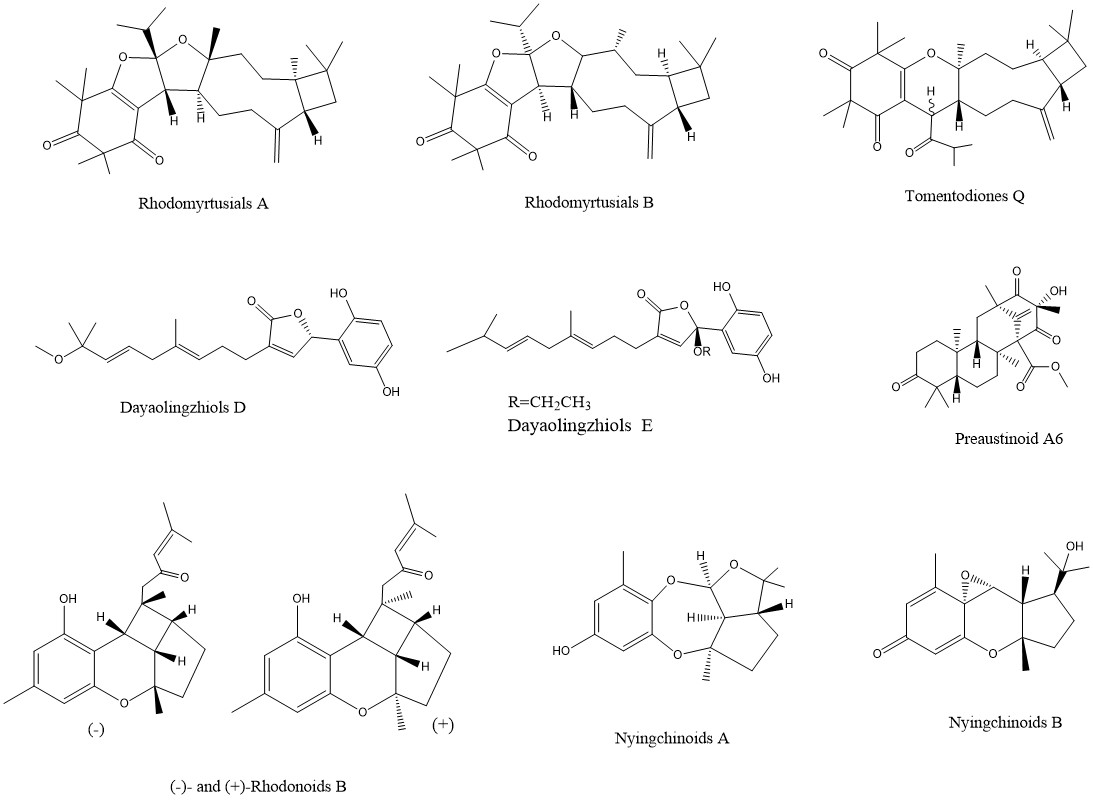

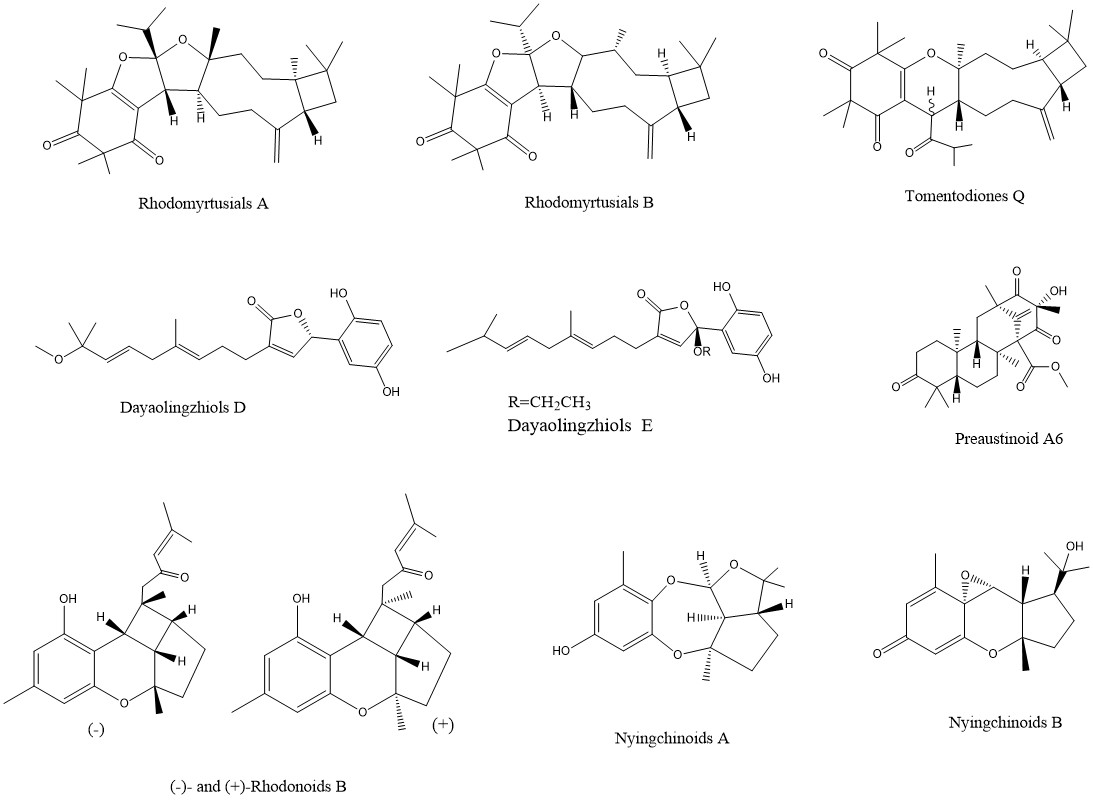

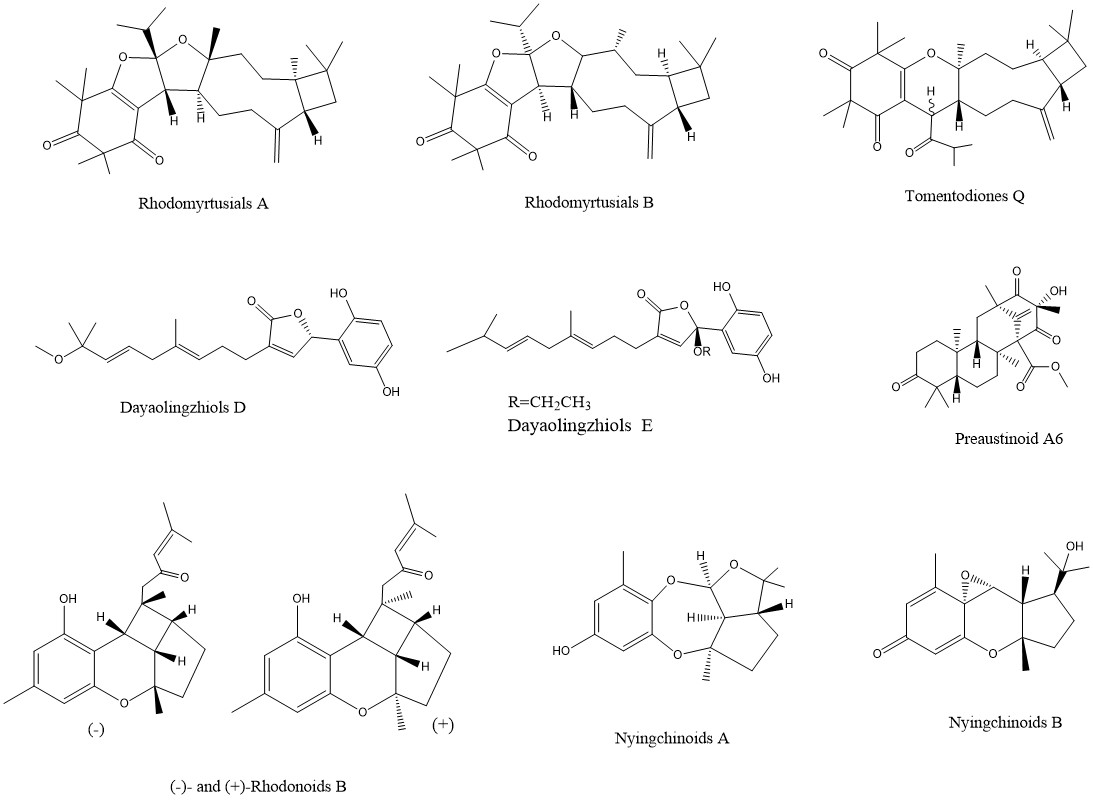

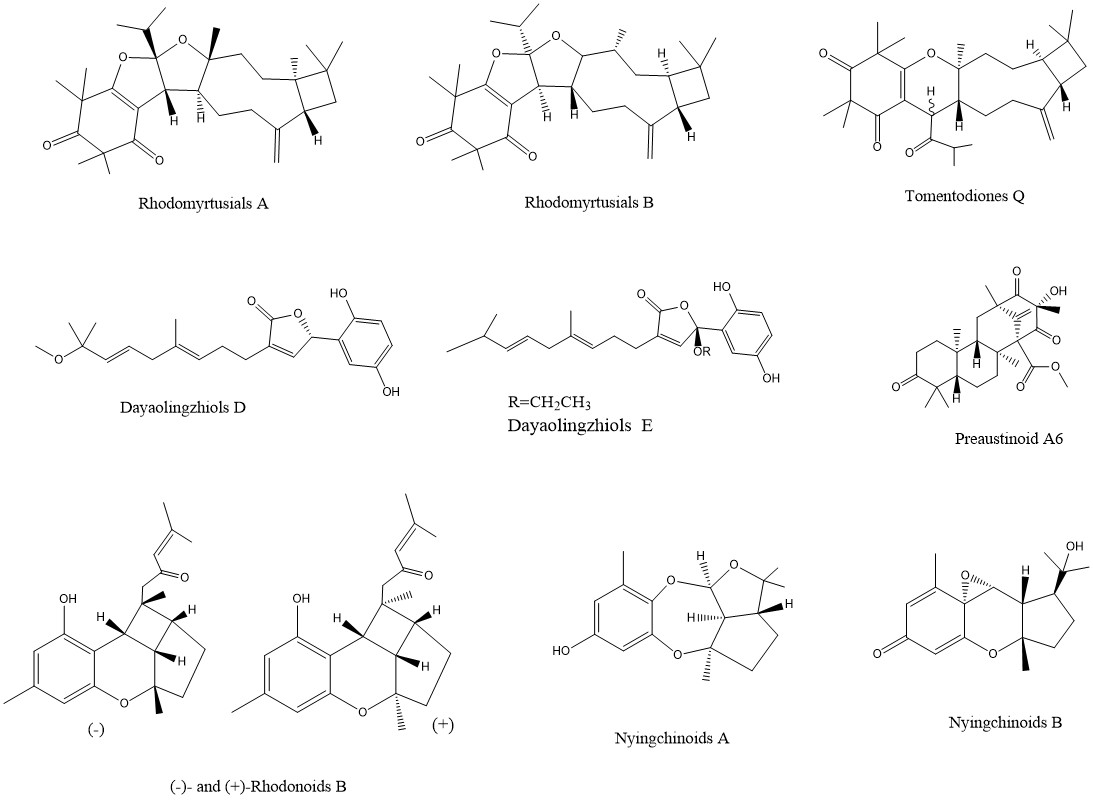

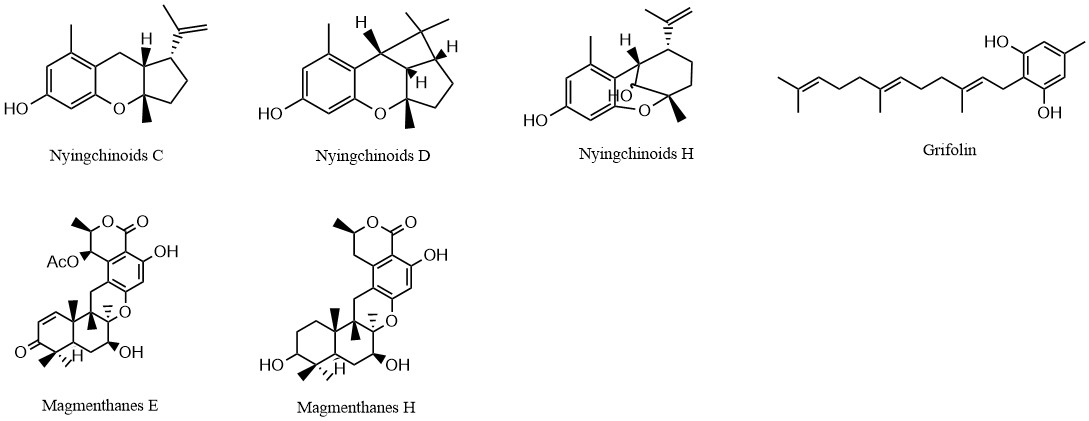

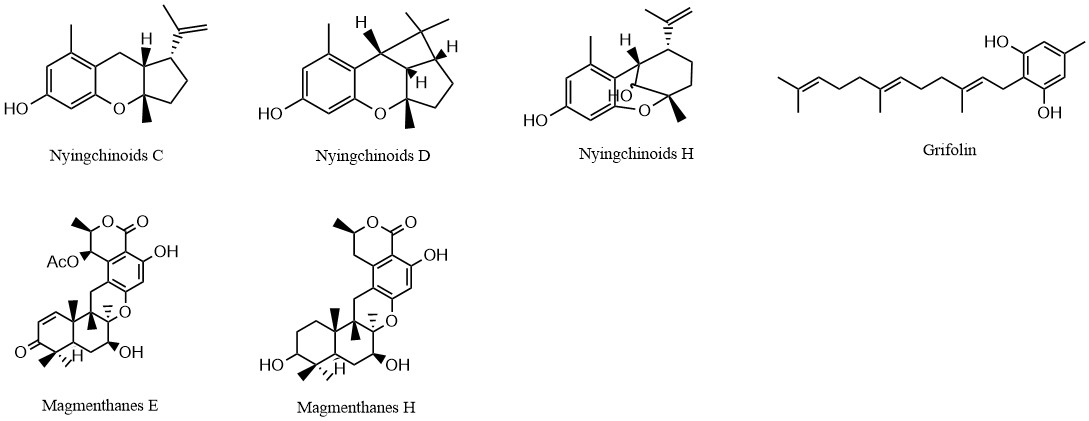


**Fig. No. 33: PTP1B activity of meroterpenoids**


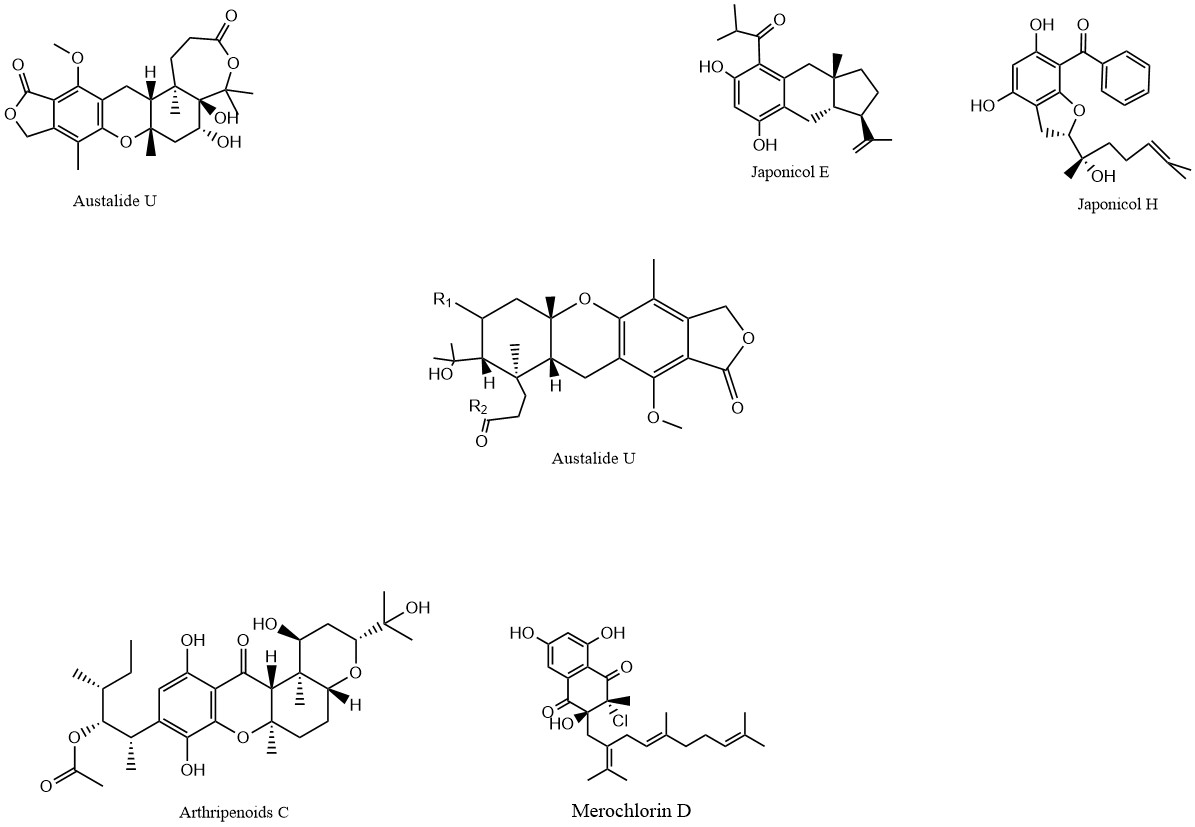


**Fig. No. 34: Meroterpenoids showing Anti-KSHV activity**


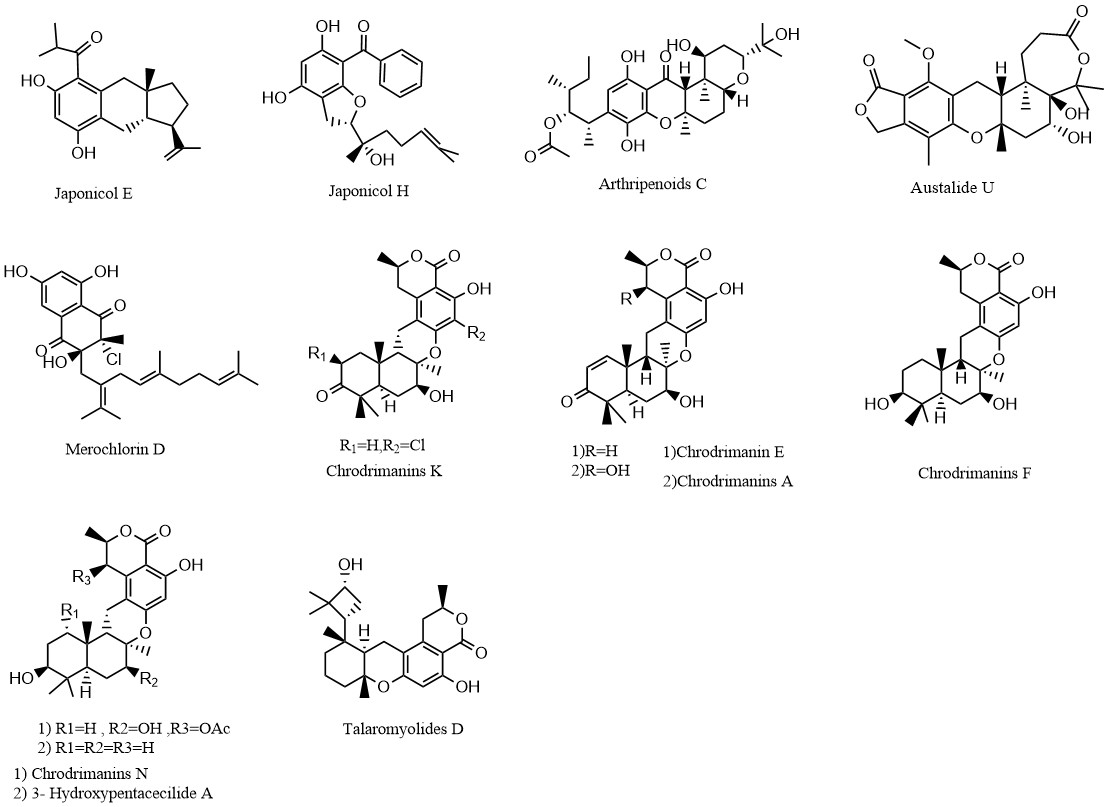

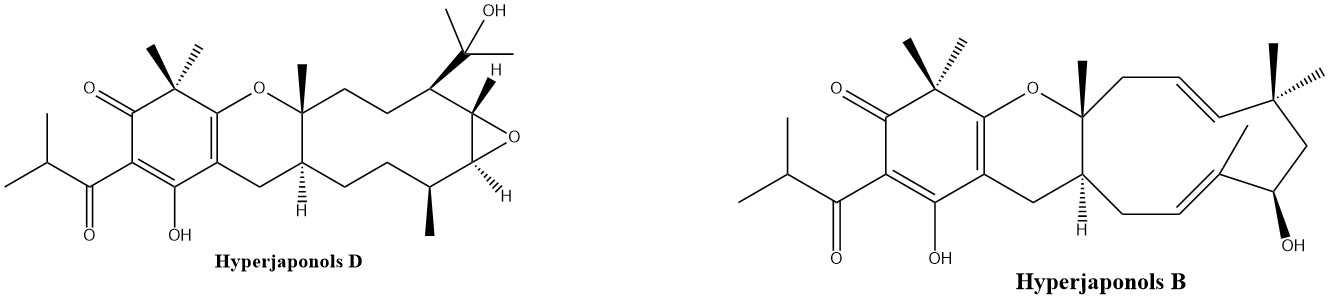


**Fig. No. 35: Meroterpenoids showing antiviral activity**


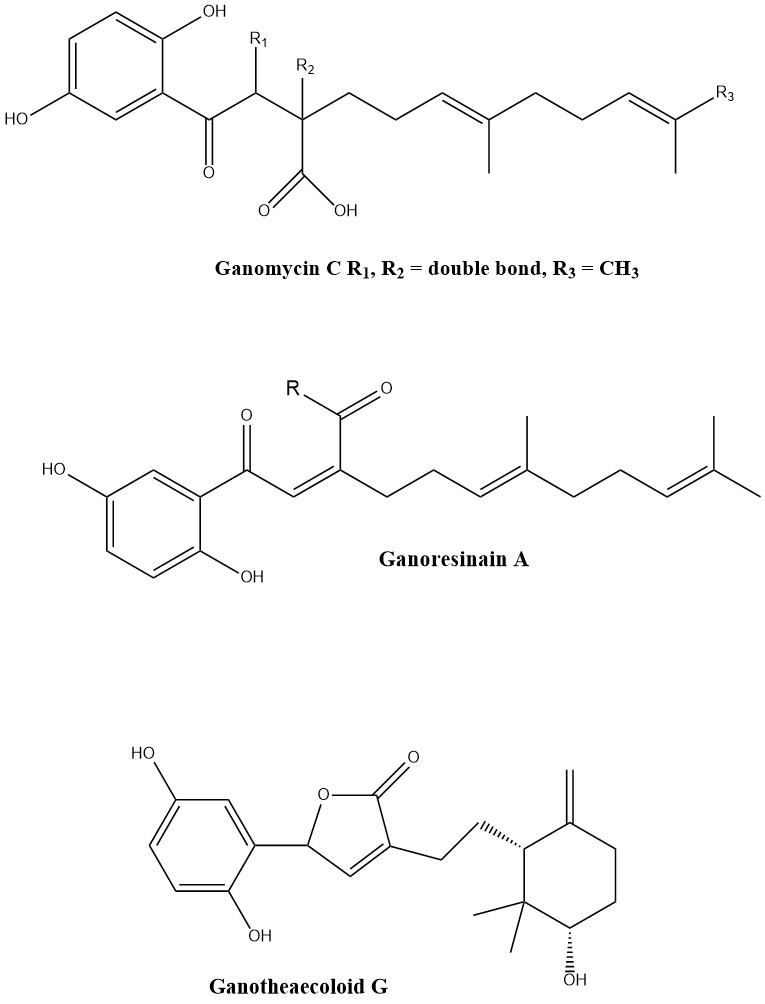


**Fig. No.36: Meroterpenoids showing neuroprotective activity**


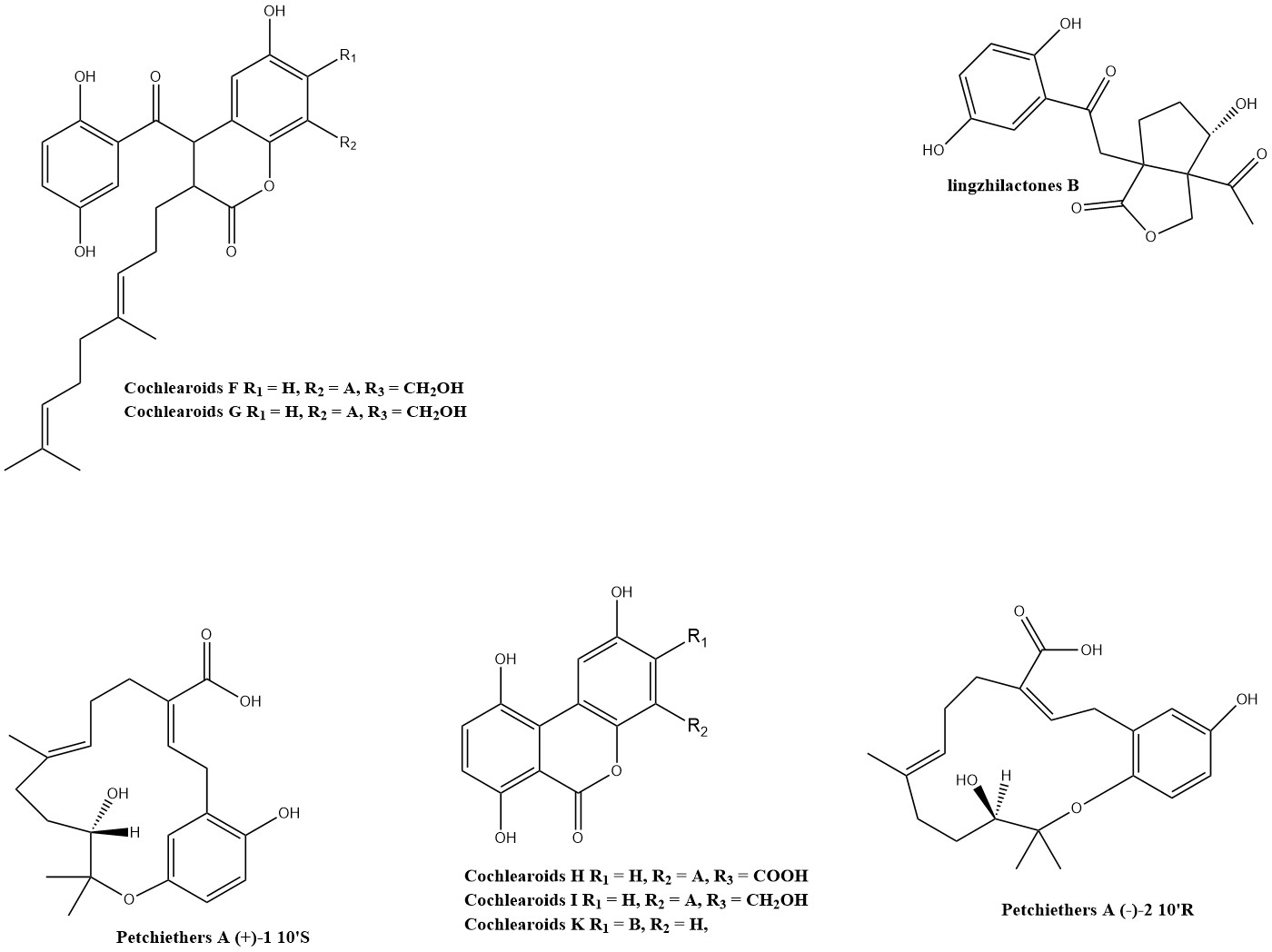

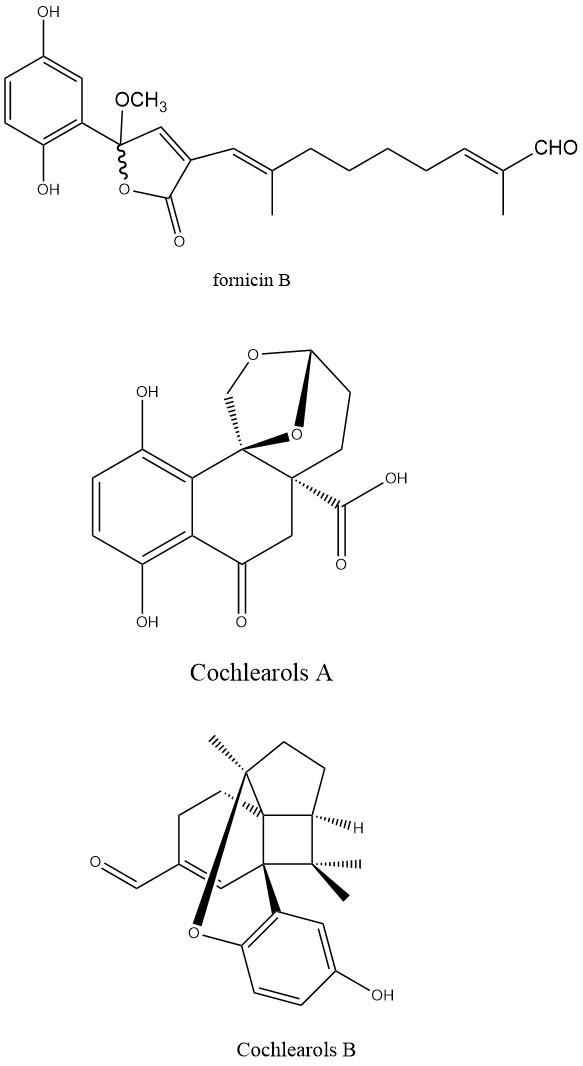


**Fig. No.37: Meroterpenoids showing renal protective activity**


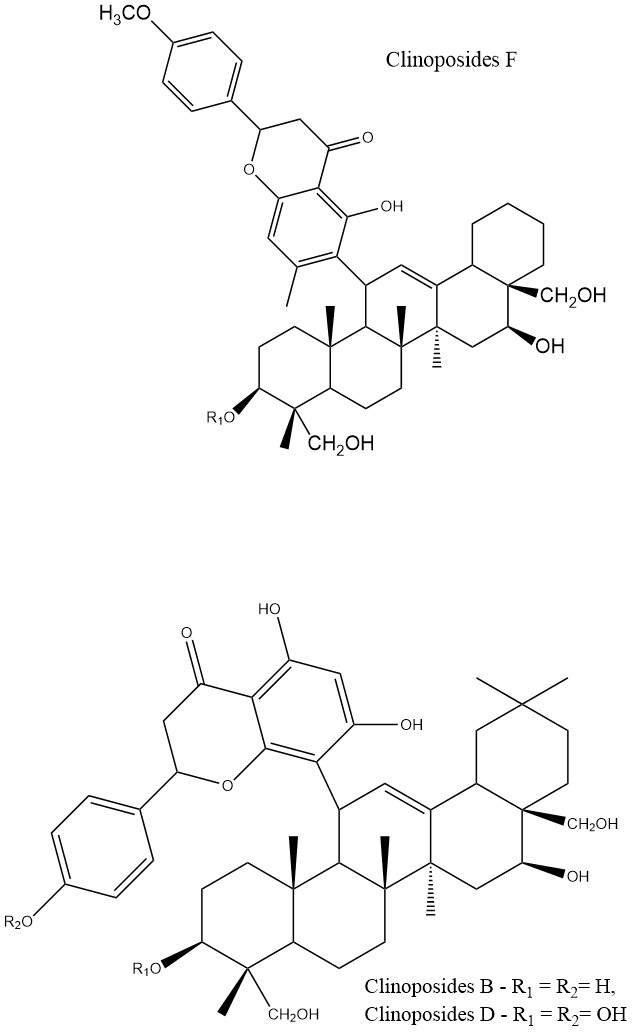


**Fig. No.38: Meroterpenoids showing cardioprotective activity**


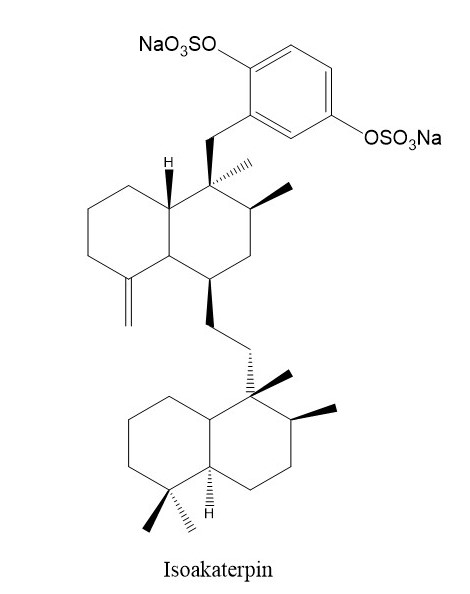


**Fig. No.39: Meroterpenoids showing anti-leishmanial activity**


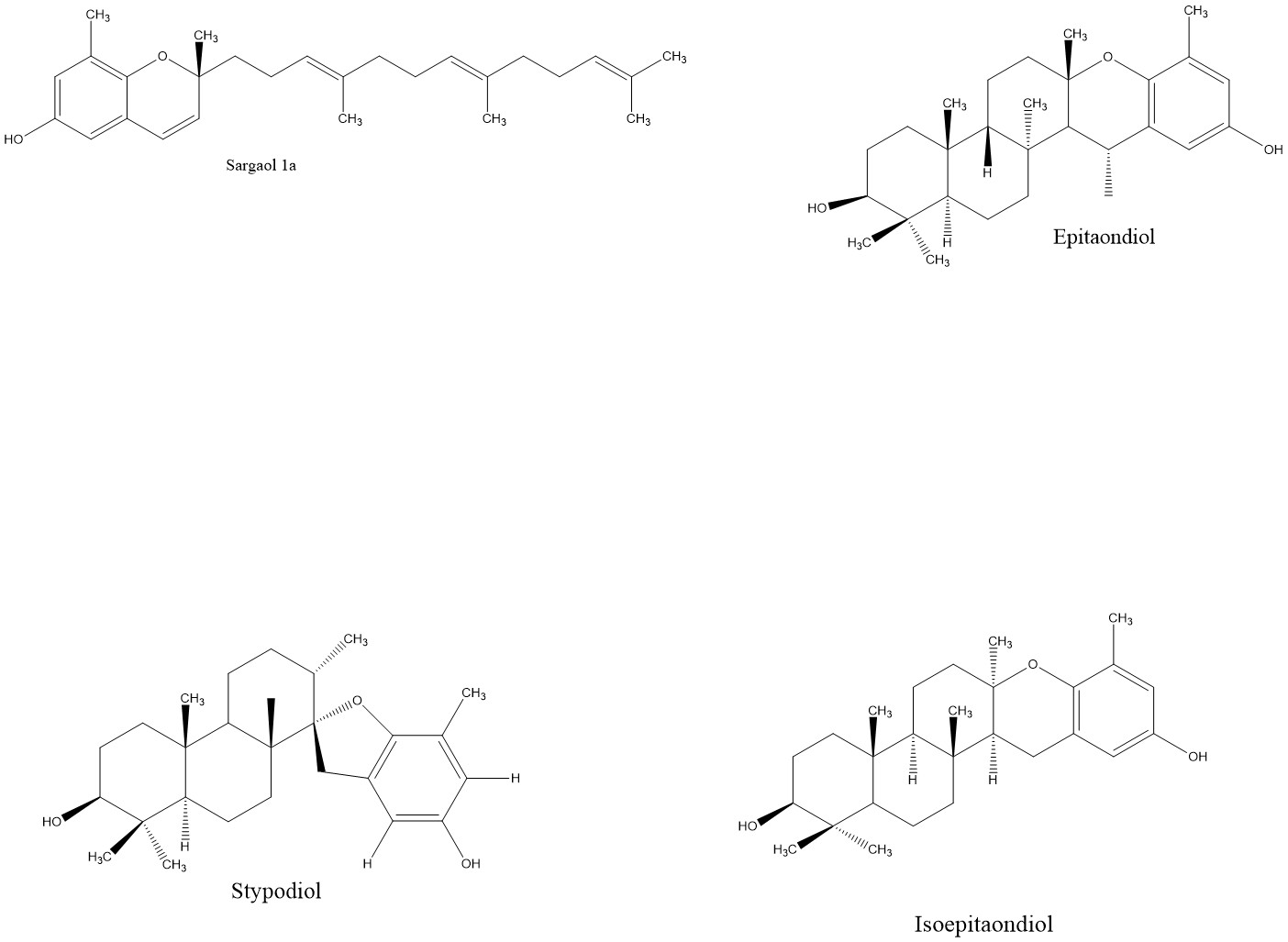


**Fig. No.40: Meroterpenoids showing gastroprotective activity**


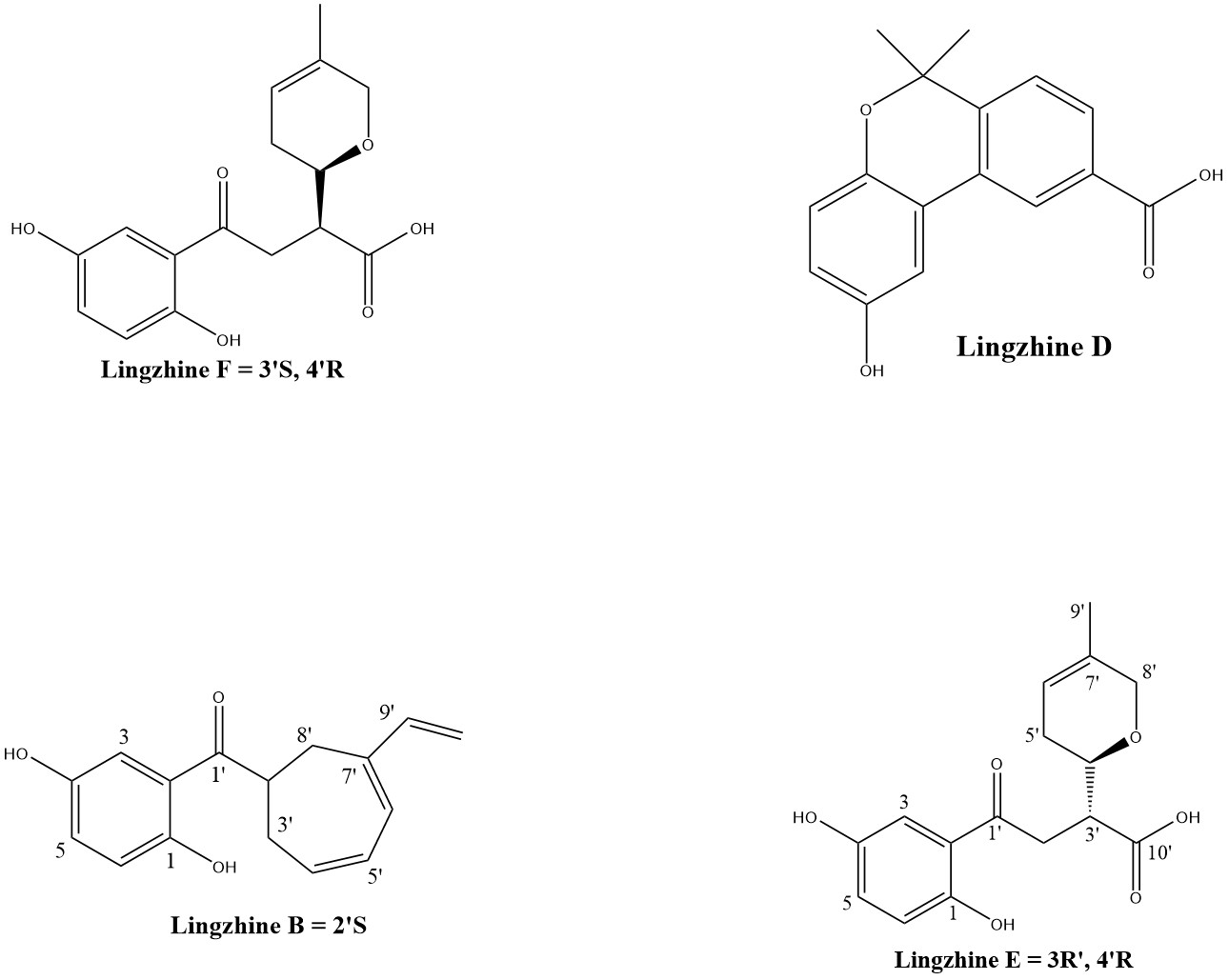

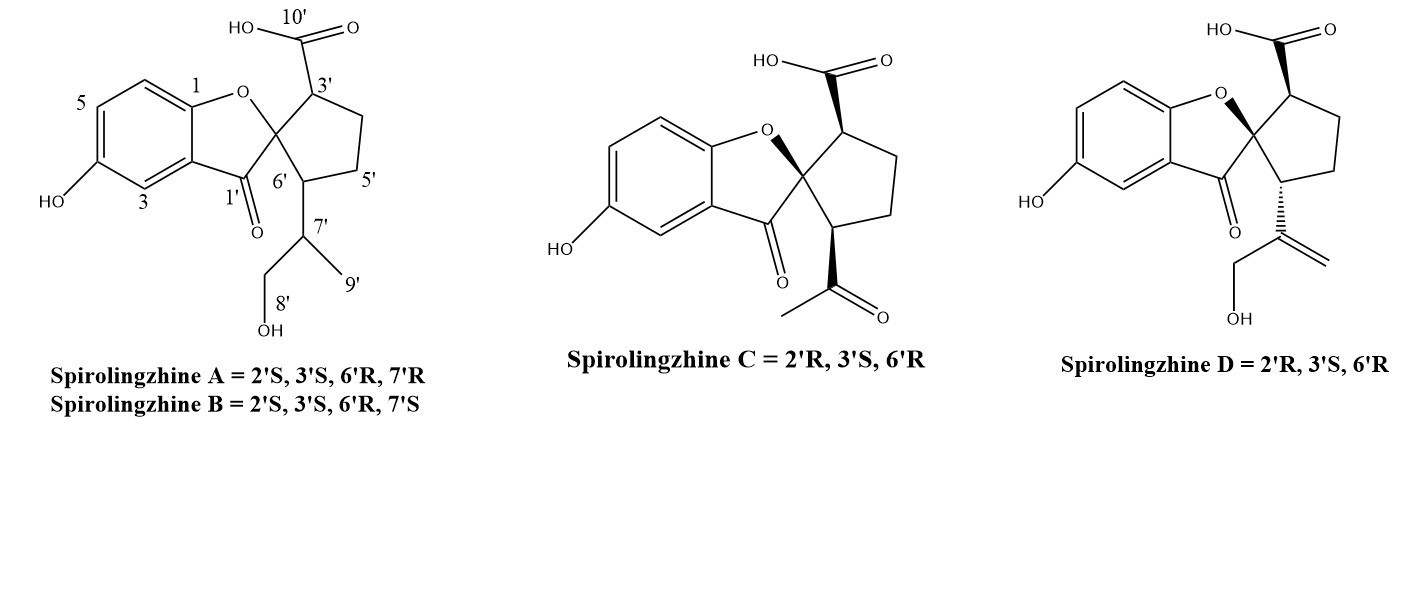


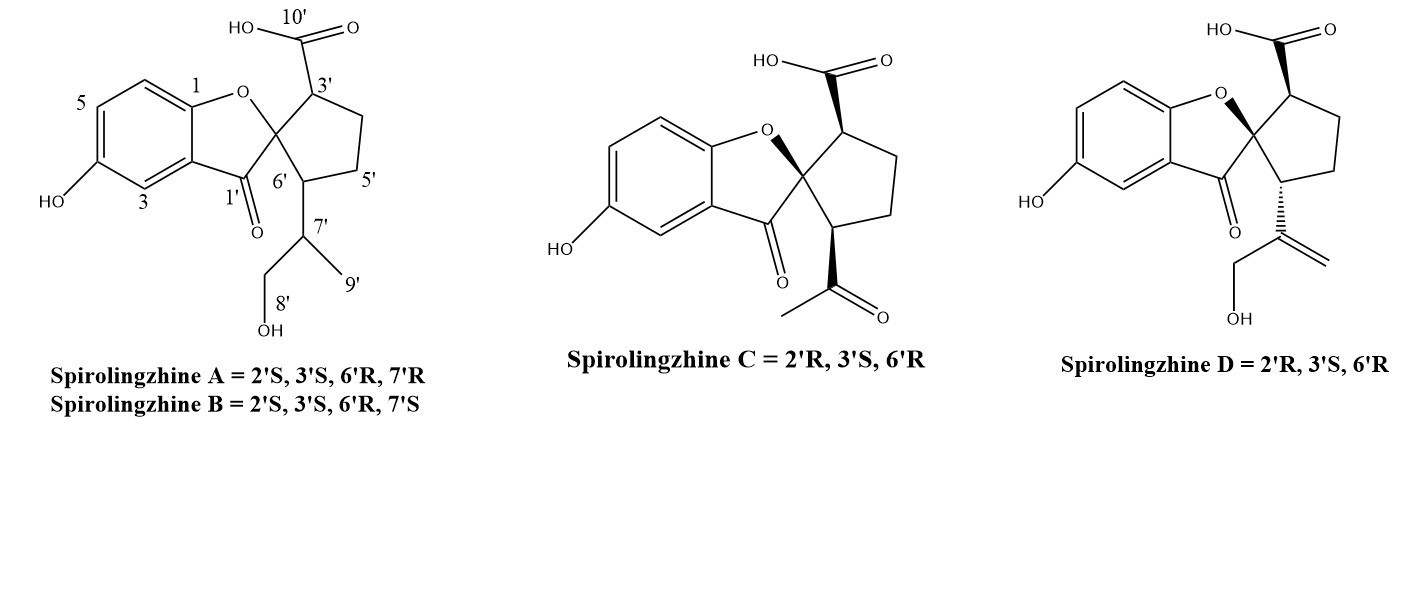

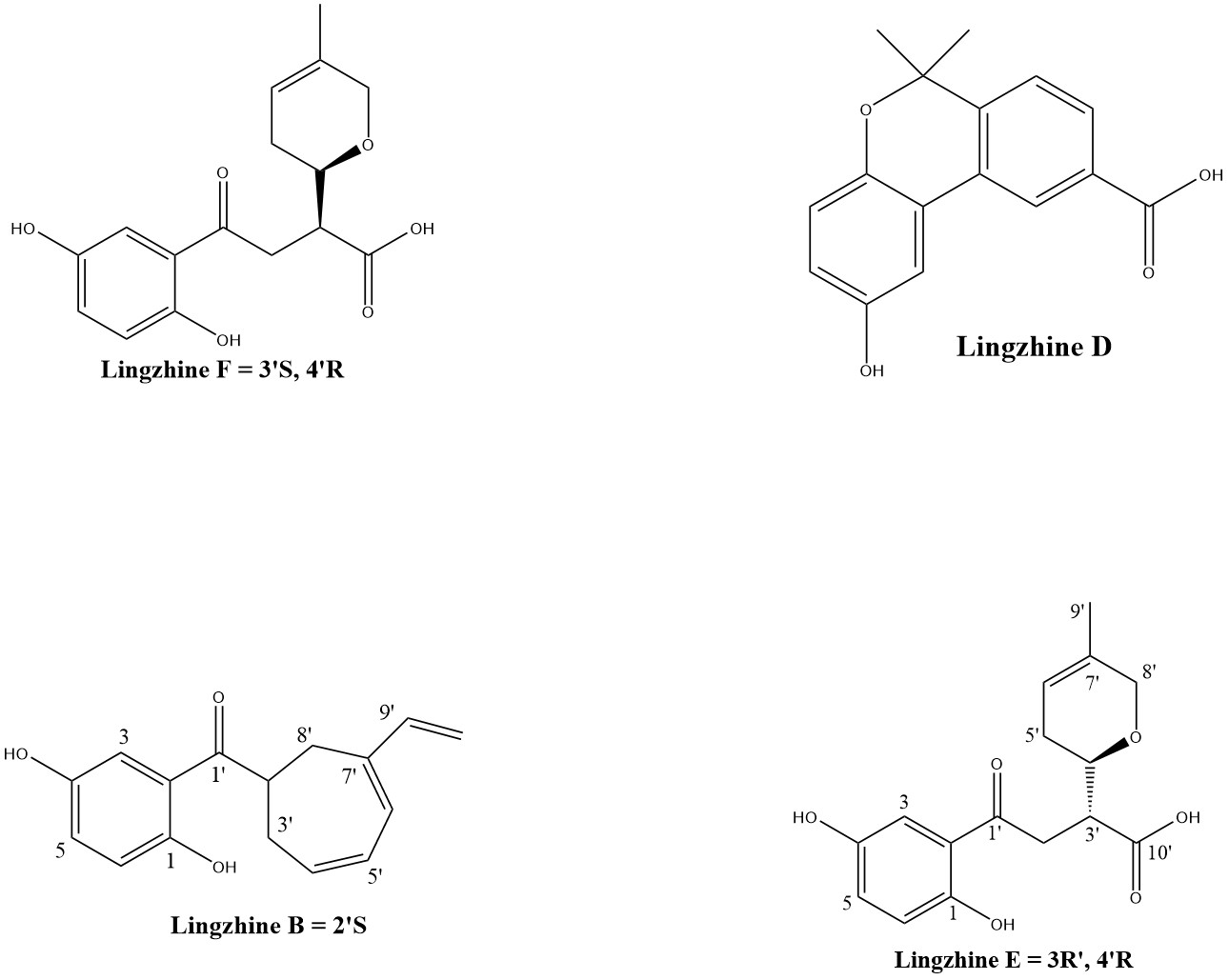

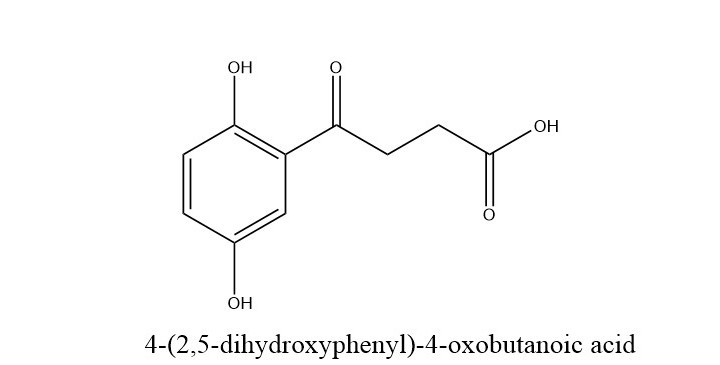

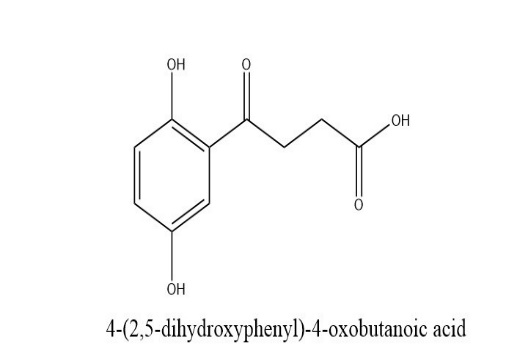


**Fig. No.41 : Meroterpenoids showing neural stem cell proliferation activity**


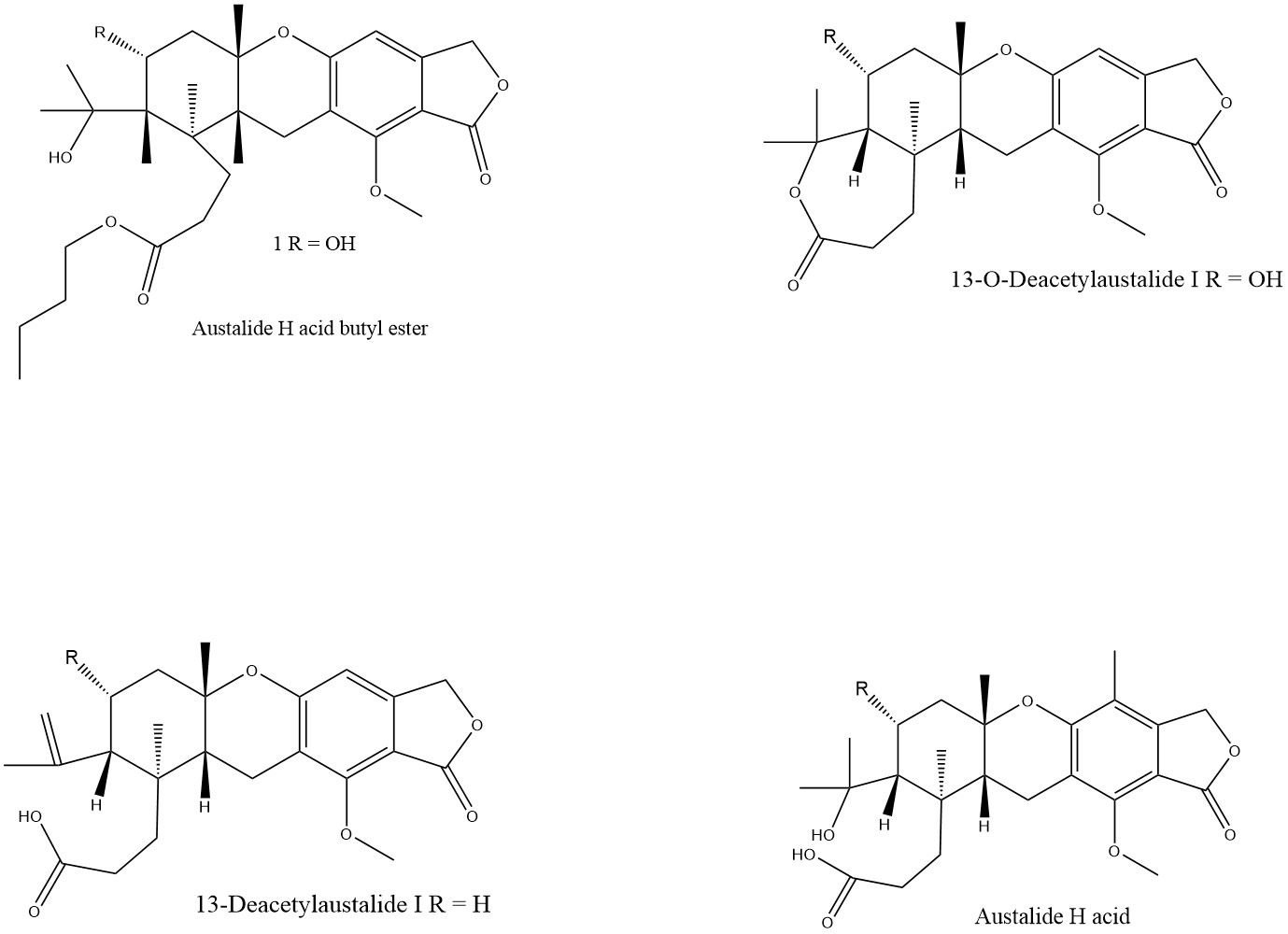


**Fig. No.42: Meroterpenoids showing AP-1 inhibition activity**


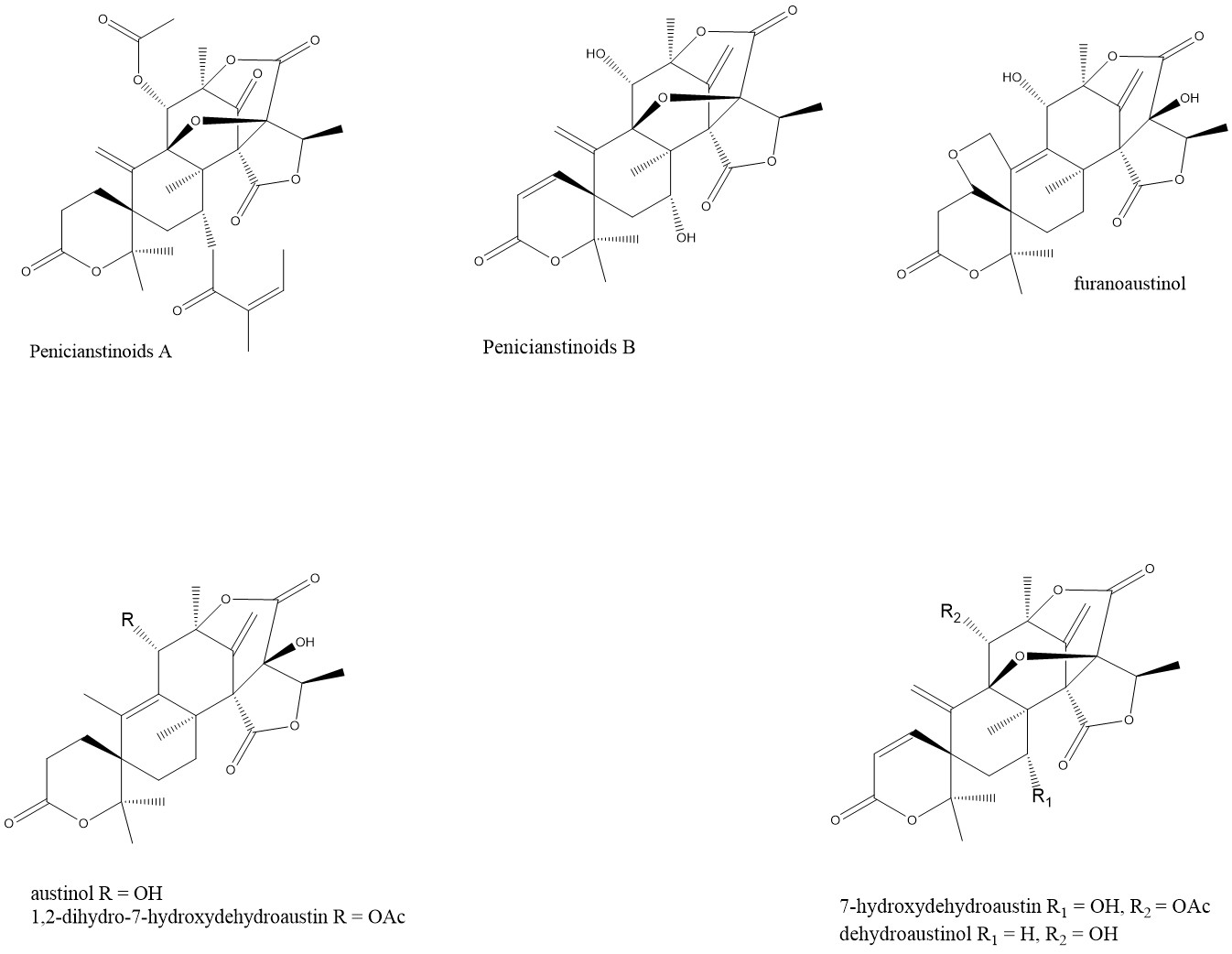


**Fig. No.43: Meroterpenoids showing insecticidal activity**


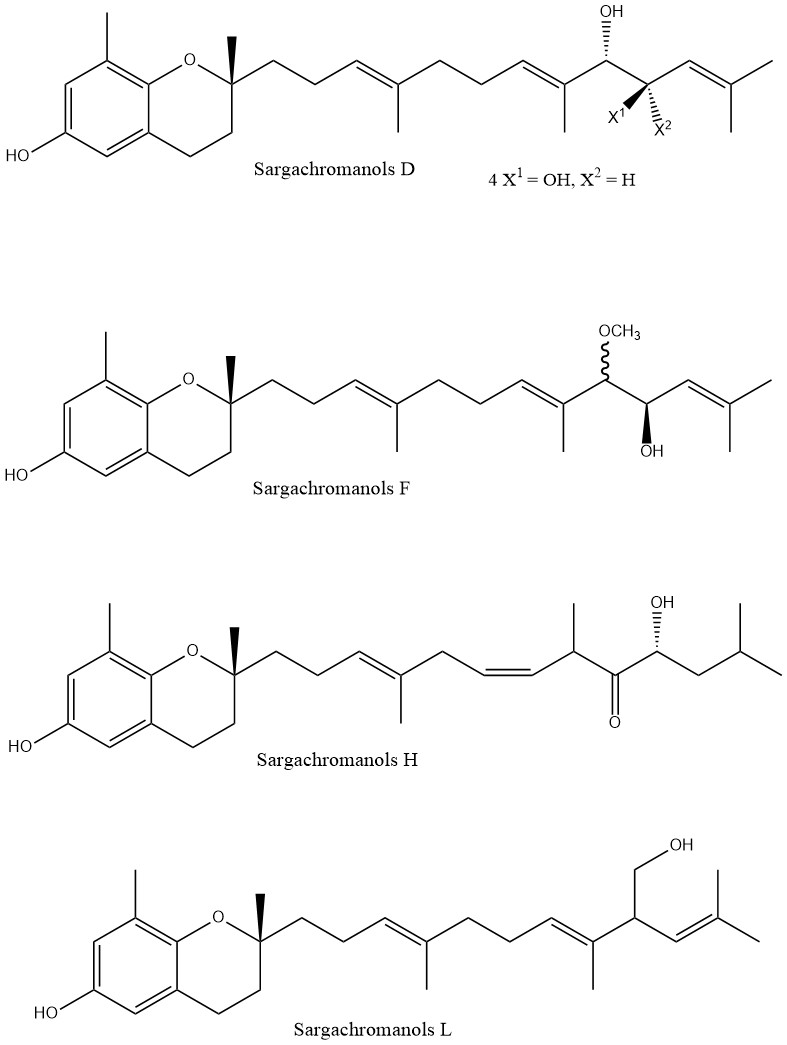


**Fig. No.44: Meroterpenoids showing Na+/K+ ATPase inhibitory activity**


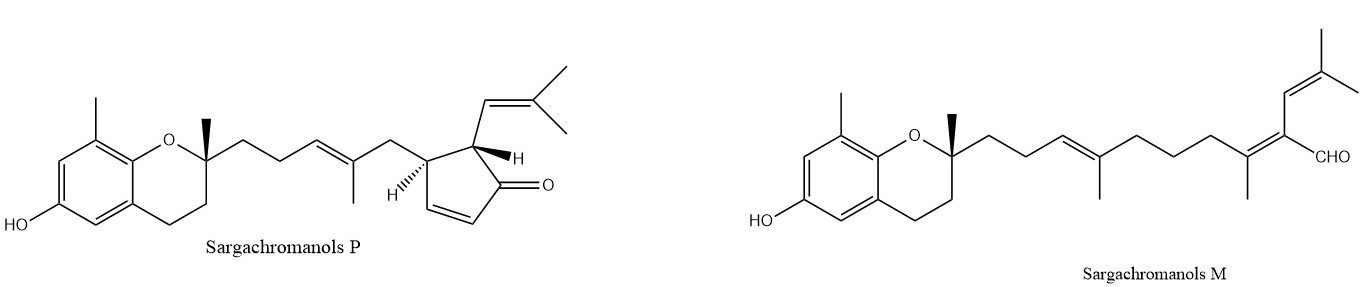


**Fig No.45: Meroterpenoids showing Isocitrate lyase inhibitory activity**


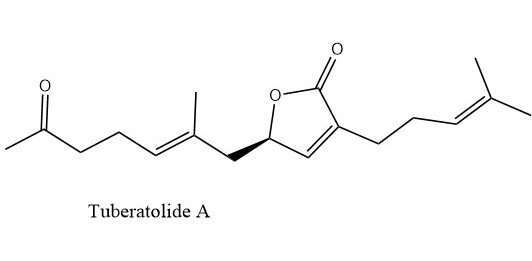


**Fig. No.46: Meroterpenoids showing activity on chenodeoxycholic acid activated human farnesoid X receptor**


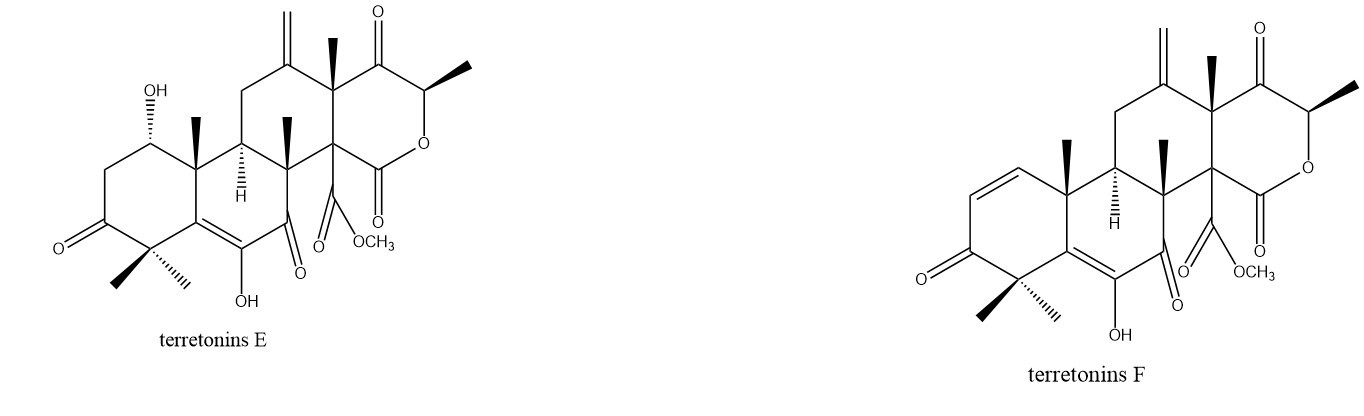


**Fig. No.47: Meroterpenoids showing mitochondrial respiratory chain inhibitory activity**


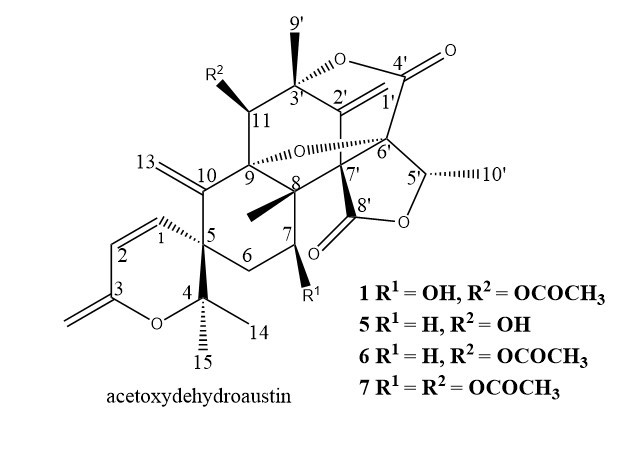


**Fig. No.48: Meroterpenoids showing larvicidal activity**

**Fig. No.49: Meroterpenoids showing anti-invasion activity**

**Fig. No.50: Meroterpenoids showing protein kinase MK2 inhibitory activity**

**Fig. No.51: Meroterpenoids showing phosphodiesterase-4 inhibitory activity**

**Fig. No.52: Meroterpenoids showing phosphodiesterase-4 inhibitory activity**

**Fig. No.53: Meroterpenoids showing phosphodiesterase-4 inhibitory activity**

**Fig. No.54: Meroterpenoids showing growth inhibition activity against newly hatched larvae of *Helicoverpa armigera***
